# Neuroprotection by Heat Shock Factor-1 (HSF1) and Trimerization-Deficient Mutant Identifies Novel Alterations in Gene Expression

**DOI:** 10.1038/s41598-018-35610-1

**Published:** 2018-11-22

**Authors:** Zhe Qu, Anto Sam Crosslee Louis Sam Titus, Zhenyu Xuan, Santosh R. D’Mello

**Affiliations:** 1 Department of Biological Sciences, Southern Methodist University, Dallas, TX 75275 USA; 2 Department of Biological Sciences, Center for Systems Biology, University of Texas at Dallas, Richardson, TX 75080 USA

## Abstract

Heat shock factor-1 (HSF1) protects neurons from death caused by the accumulation of misfolded proteins by stimulating the transcription of genes encoding heat shock proteins (HSPs). This stimulatory action depends on the association of trimeric HSF1 to sequences within HSP gene promoters. However, we recently described that HSF-AB, a mutant form of HSF1 that is incapable of either homo-trimerization, association with HSP gene promoters, or stimulation of HSP expression, protects neurons just as efficiently as wild-type HSF1 suggesting an alternative neuroprotective mechanism that is activated by HSF1. To gain insight into the mechanism by which HSF1 and HSF1-AB protect neurons, we used RNA-Seq technology to identify transcriptional alterations induced by these proteins in either healthy cerebellar granule neurons (CGNs) or neurons primed to die. When HSF1 was ectopically-expressed in healthy neurons, 1,211 differentially expressed genes (DEGs) were identified with 1,075 being upregulated. When HSF1 was expressed in neurons primed to die, 393 genes were upregulated and 32 genes were downregulated. In sharp contrast, HSF1-AB altered expression of 13 genes in healthy neurons and only 6 genes in neurons under apoptotic conditions, suggesting that the neuroprotective effect of HSF1-AB may be mediated by a non-transcriptional mechanism. We validated the altered expression of 15 genes by QPCR. Although other studies have conducted RNA-Seq analyses to identify HSF1 targets, our study performed using primary neurons has identified a number of novel targets that may play a special role in brain maintenance and function.

## Introduction

Neurons are particularly sensitive to the accumulation of misfolded proteins. Under normal circumstances misfolded proteins are either refolded or degraded by chaperones, the best studied and most effective of which are the heat shock proteins (HSPs). It is widely believed that the late onset of neuronal loss in neurodegenerative diseases is the result of an age-related decline in the functioning of HSPs and protein degradation machinery leading to an increasing inability to refold or clear misfolded proteins. Consistent with this idea, several studies have described that elevating HSP activity either by overexpression or pharmacologically is protective in *in vivo* models of various neurodegenerative disorders^1–5^. The increased synthesis of HSPs in response to protein misfolding is driven by the DNA-binding transcription factor, heat shock factor-1 (HSF1). Although always nuclear in some cells, in many cell types HSF1 is retained in the cytoplasm in a monomeric form within a protein complex containing certain HSPs. Upon exposure to heat or protein-damaging stress, the HSPs are diverted to the newly misfolded proteins freeing HSF1 to translocate to the nucleus where it trimerizes. Trimeric HSF1 binds to heat shock elements (HSEs) within the promoters of genes encoding HSPs to induce their transcription^6–8^. Since monomeric HSF1 cannot bind to the HSE, HSF1 trimerization is an obligatory step in the transcriptional activation of HSP genes. As observed with HSPs, the direct overexpression of HSF1 is also protective in several models of neurodegenerative disease^9–14^. Because HSF1 can stimulate HSP production it is widely assumed that the neuroprotective effect of HSF1 is mediated by HSPs. Indeed, whether HSF1 can protect against neurodegeneration by mechanisms independent of HSP stimulation has not been seriously investigated. We recently conducted a study aimed at examining the role of HSF1, both in healthy neurons as well as in neurons induced to die by proteotoxic and non-proteotoxic stimuli. We found that HSF1 was necessary for the survival of neurons normally and that elevating HSF1 expression protected neurons not just from non-proteotoxic death, but also in models where there was no protein misfolding or aggregation^15^. Most interestingly, we found that the neuroprotective effect of HSF1 did not require its trimerization or its binding to an HSE, and was mediated by an HSP-independent mechanism^15^. Indeed, a mutant form of HSF1 lacking the trimerization domain and that was unable to bind to HSEs was just as neuroprotective as normal HSF1. One possibility to explain neuroprotection by trimerization-deficient HSF1 is that HSF1 can stimulate expression of non-HSP neuroprotective genes by binding as a monomer to sequences different from HSEs in the promoters of these genes. Results from other studies are consistent with the idea that non-HSP mechanisms regulate HSF1-mediated neuroprotection. For example, HSF3 protects mouse embryonic fibroblast cells from heat shock-induced death just as efficiently as HSF1 although it is incapable of activating HSPs^16^. Also, protection by HSF1 against polyQ toxicity has been reported to be mediated by stimulating expression of NFATc2^17^.

As a means of investigating the possibility that HSF1 may mediate neuroprotection by regulating gene transcription as a monomer, we conducted RNA-Seq analysis aimed at identifying genes that were regulated by trimerization-deficient HSF1 (HSF1-AB). We report that contrary to our expectations, we found only a total of 19 genes that were regulated by HSF1-AB (13 in healthy neurons and 6 under apoptotic conditions). This is in contrast with HSF1, which regulated the expression of a total of 1,266 genes under these conditions (1,211 in healthy neurons and 425 in apoptotic neurons). The modest alteration in gene expression by HSF1-AB suggests that, in contrast to wild-type HSF1, monomeric HSF1 may not protect neurons by acting as a transcription factor. Consistent with this conclusion is the observation that whereas wild-type HSF1 localizes primarily to the nucleus, HSF1-AB distributes in both the cytoplasm and the nucleus. It is likely that neuroprotection by HSF1-AB involves protein-protein interactions or some other non-transcriptional mechanisms.

## Results

### Overexpression of HSF1 and HSF1-AB in cerebellar granule neurons (CGNs)

**Figure 1:**
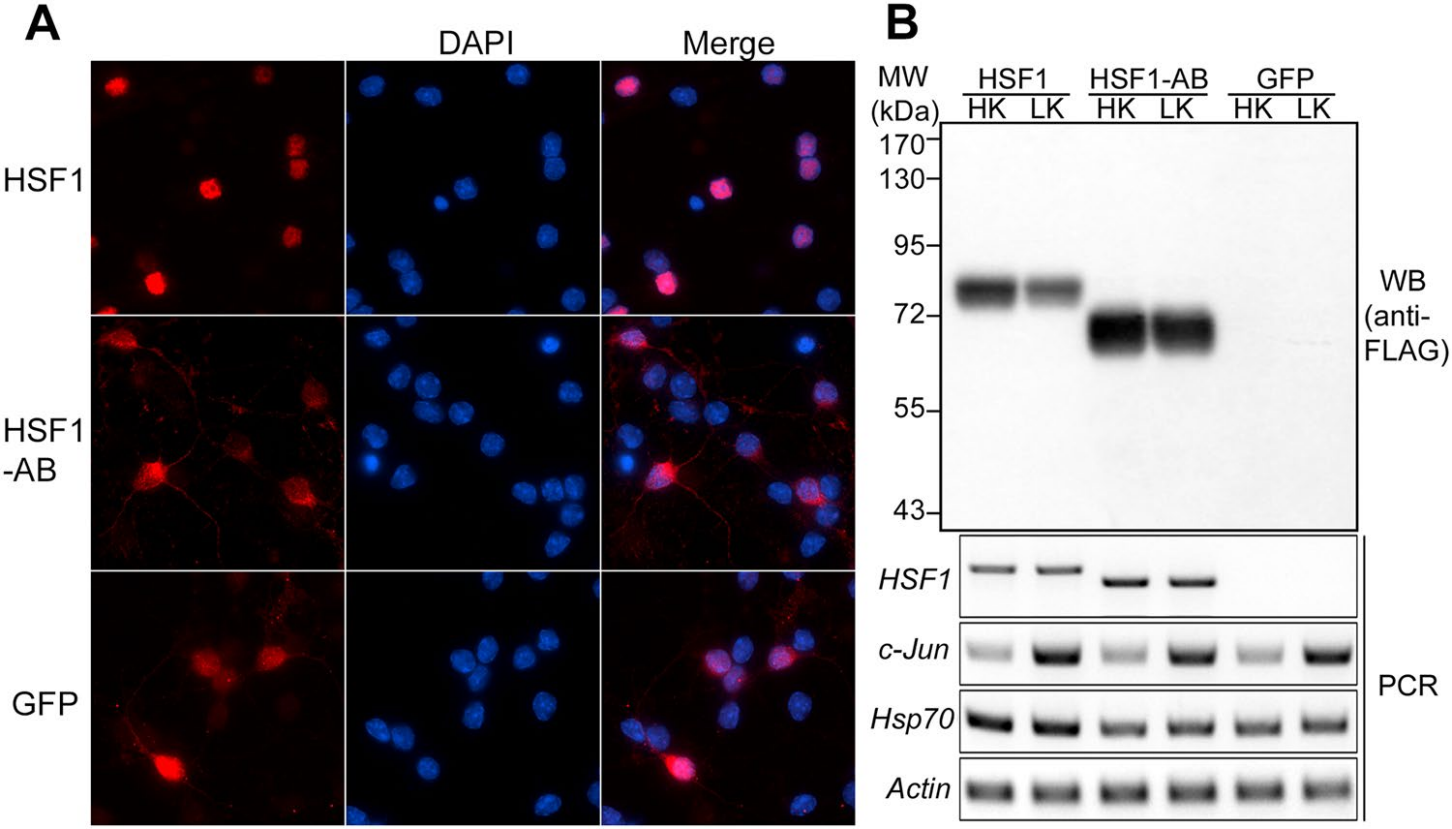
Overexpression of HSF1 and HSF1-AB in CGNs. (**A**) CGNs were infected with Ad-HSF1, Ad-HSF1-AB, or Ad-GFP and treated with HK or LK for 8 hours. Immunostaining using FLAG or GFP antibody showed that HSF1 was mainly localized in the nucleus, while HSF1-AB and GFP were distributed in both the cytoplasm and the nucleus. (**B**) HSF1 and HSF1-AB were robustly expressed in the CGNs as shown by Western blot (WB) and RT-PCR analyses. Expression level of *Hsp70*, a known target gene of trimeric HSF1, was upregulated by wild-type HSF1 but not by HSF1-AB, which lacks the trimerizaiton domain. Level of an apoptotic marker, *c-Jun*, was dramatically increased by LK treatment. The darker intensity of signal for HSF1-AB relative to HSF1 is because of its higher stability (Qu and D’Mello, manuscript in preparation).

HSF1 and HSF1-AB, a deletion mutant of HSF1 lacking the entire trimerization domain (156–226 aa), were overexpressed in CGNs for 40 hours via adenovirus infection followed by HK (high potassium) or LK (low potassium) treatment for 8 hours. Removing potassium from the culture medium (LK) can stimulate apoptosis of CGNs, and has therefore been used widely as a cellular cell death model. As described previously, immunocytochemistry analysis showed that HSF1 was localized to the nucleus^15^. In contrast, HSF1-AB was distributed in the cytoplasm and the nucleus (Fig. 1A). Using Western-blotting and RT-PCR, we confirmed that both HSF1 and HSF1-AB were robustly overexpressed in the CGNs (Fig. 1B). As expected, expression of *Hsp70*, which requires trimeric HSF1 to bind to its promoter, was elevated by HSF1 overexpression, but not by HSF1-AB (Fig. 1B). The level of *c-Jun*, a biomarker for apoptosis, was upregulated in LK-treated CGNs.

### Gene expression profiles in CGNs overexpressing HSF1 or HSF1-AB in HK/LK conditions

**Figure 2:**
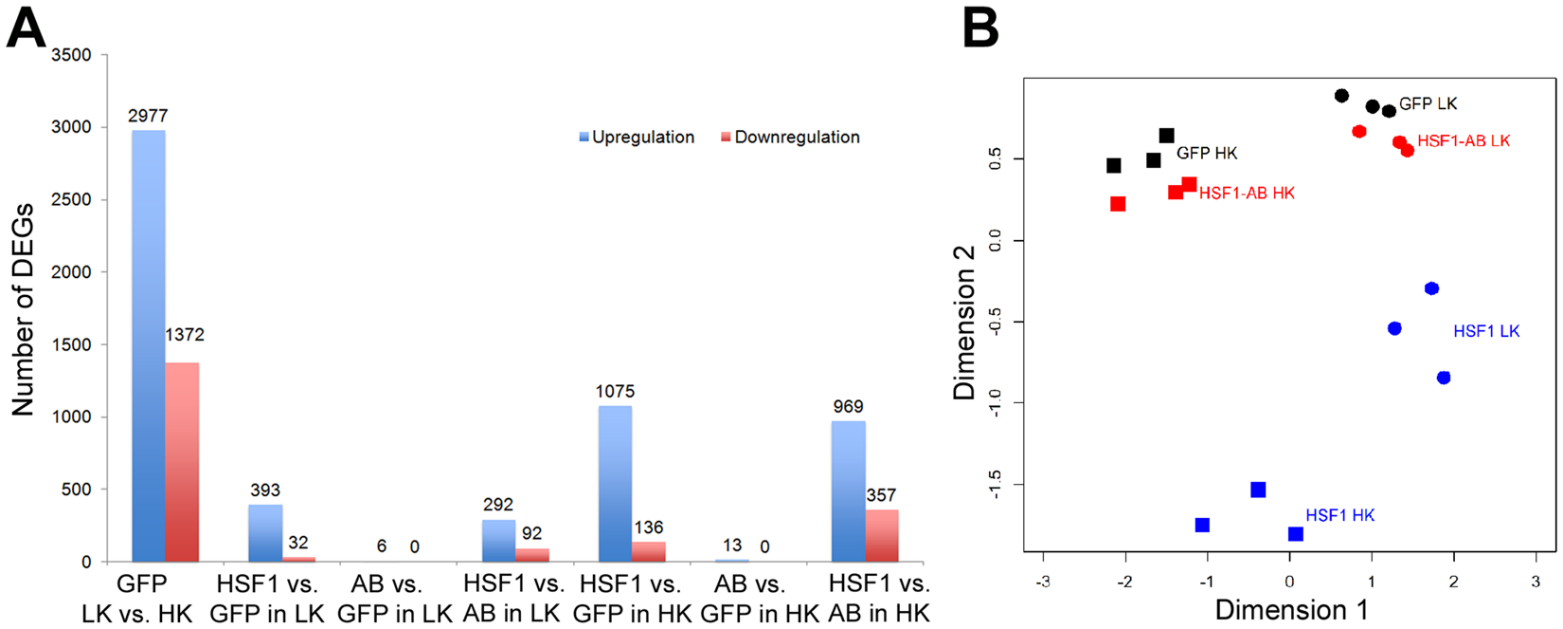
RNA-Seq analysis to profile gene expression changes in CGNs overexpressing HSF1 or HSF-AB under HK/LK condition. The six samples (HSF1 HK, HSF1 LK, HSF1-AB HK, HSF1-AB LK, GFP HK, and GFP LK) were analyzed by RNA-Seq. (**A**) The numbers of DEGs obtained in different comparisons were summarized in the bar graph. (**B**) The similarity of the six samples in biological triplicates was visualized by a MDS plot.

In order to understand the underlying mechanisms of the protective effects of HSF1 and HSF1-AB in neurons primed to die, we performed RNA-Seq analysis using samples from three separate experiments with the same conditions as in Fig. 1. The gene expression profiles in the six samples, including HSF1 HK, HSF1 LK, HSF1-AB HK, HSF1-AB LK, GFP HK, and GFP LK are shown in Fig. 2A (false discovery rate (FDR) < 0.05, fold change > 1.5). Compared to GFP HK, a total of 2,977 and 1,372 genes were up- and down-regulated in GFP LK (Fig. 2A). A total of 425 differentially expressed genes (DEGs; 393 upregulation and 32 downregulation) were identified by ectopic expression of HSF1 compared to GFP in LK, while HSF1-AB only altered 6 genes. There were 384 genes (292 upregulation and 92 downregulation) differentially expressed between HSF1 LK and HSF1-AB LK. In HK, 1,211 genes were changed by HSF1 compared to GFP. However, only 13 genes were changed by HSF1-AB compared to GFP in HK. Multi-Dimensional Scaling (MDS) plot of samples generated with gene expression profiles showed that the HSF1-AB samples are more similar to the GFP control, while HSF1 samples are much further apart, which indicates that HSF1 has a much stronger impact on gene regulation than HSF1-AB does (Fig. 2B).

### Genes regulated by HSF1 in CGNs undergoing apoptosis

**Figure 3:**
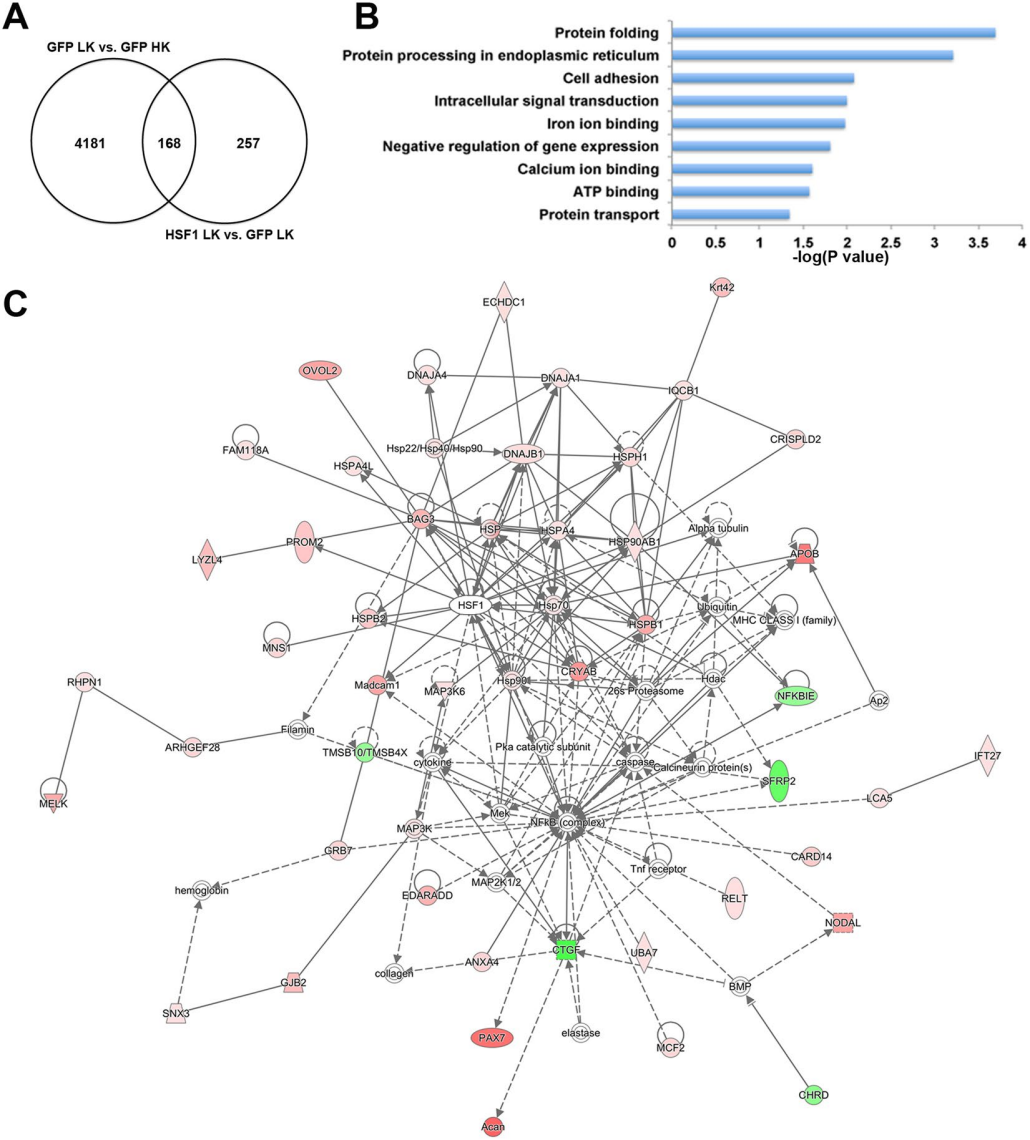
Genes regulated by HSF1 in LK-treated CGNs. (**A**) A total of 425 genes were regulated by HSF1 in LK compared to the control, GFP LK. As shown in the Venn diagram, 168 of these DEGs were also found altered by LK treatment in GFP control sample (vs. GFP HK). (**B**) Gene Ontology analysis showed that the 425 HSF1 target genes were involved in various biological processes, including protein folding, protein processing in endoplasmic reticulum, and intracellular signal transduction. The top protein network that these DEGs participate in functions in cellular assembly and organization, cellular compromise, and post-translational modification (**C**). Red color represents upregulation by HSF1 while green color indicates downregulation. The color intensity shows the degree of level change. Proteins in white shapes do not belong to our data set but have relationships with our proteins in the network.

The genes that were regulated by HSF1 compared to GFP control in HK and LK are shown in supplemental Table S1 (1,211 DEGs) and Table S2 (425 DEGs), respectively. Since there are 370 DEGs shared by these two data sets, HSF1 regulates a total of 1,266 genes in either HK or LK. The top DEGs for each condition according to fold change are listed in Tables 1 and 2. Among the 425 HSF1-regulated genes in apoptotic CGNs, 168 were also altered by LK alone (Fig. 3A). HSF1 enhances the expression of some of these 168 genes beyond what is observed with LK alone. Although this seems counterintuitive, it is likely that these are protective genes upregulated by neurons that are making an effort to stay alive or by the subpopulation of neurons that are more resistant and dying at a slower rate. It may be noted that in this model of neuronal death about 50% of the neurons die within 24 hours with most of the remaining neurons dying over the next 24 to 48 hours^18^.

**Table 1:** Top HSF1-regulated genes in healthy CGNs.

Genes	Fold change	Protein description
Slc34a2*	616.946	Sodium-dependent phosphate transport protein 2B
Crybb1	308.205	Beta-crystallin B1
Krt42	195.526	Keratin, type I cytoskeletal 42
Crabp1	122.264	Cellular retinoic acid-binding protein 1
Apob*	112.260	Apolipoprotein B
Ovol2	95.548	Ovo-like zinc finger 2, C2H2 zinc finger transcription factor of the Ovo-like family
Angptl1	94.663	Angiopoietin-like 1, ligand for endothelial tyrosine-kinase receptor Tek
Nmb*	88.659	Neuromedin B, bombesin-related peptide
Plac9*	88.374	Placenta-specific 9
Kank4	79.895	KN motif and ankyrin-repeat domain-containing protein 4
Myo1h	79.506	Myosin IH
Rfx6	76.643	Regulatory factor X6, transcription factor of the RFX family
Pitx2	71.177	Paired-like homeodomain transcription factor 2
Sox15*	66.873	SRY box 15, transcription factor of the SOX family
Pax7*	59.913	Paired box 7, transcription factor of the paired box family
Igfbpl1*	59.809	Insulin-like growth factor binding protein-like 1
Nodal	55.864	Nodal growth differentiation factor, morphogen, ligand of the TGFβ signaling pathway
Tchh	54.351	Trichohyalin
Mcoln2	51.025	Mucolipin-2, cation channel protein
Gipc3	50.950	GIPC PDZ domain-containing family member 3

Note: Uncharacterized genes are not listed. *Common genes between HK and LK.

**Table 2:** Top HSF1-regulated genes in apoptotic CGNs.

Genes	Fold change	Protein description
Rbm12	107.500	RNA-binding motif protein 12
Dmrt1	63.926	Doublesex and mab-3 related transcription factor 1
Acan	34.704	Aggrecan, extracellular matrix protein
Slc34a2*	33.350	Sodium-dependent phosphate transport protein 2B
Zfp93	26.539	Zinc finger protein 93
Alx4	26.088	ALX homeobox 4, paired-like homeodomain transcription factor
Pax7*	25.004	Paired box 7, transcription factor of the paired box family
Apob*	20.516	Apolipoprotein B
Fbxw10	16.614	F-box and WD repeat domain-containing 10, targeting proteins for ubiquitination
Nmb*	16.136	Neuromedin B, bombesin-related peptide
Igfbpl1*	15.883	Insulin-like growth factor binding protein-like 1
Plac9*	14.963	Placenta-specific 9
Batf	12.539	Basic leucine zipper ATF-like transcription factor
Crhr2	11.869	Corticotropin-releasing hormone receptor 2
Cryab	11.292	Crystallin alpha B, heat shock protein of the crystallin family
Sctr	10.478	Secretin receptor
Spetex-2F	10.413	Spetex-2F protein
Sox15*	10.363	SRY box 15, transcription factor of the SOX family
Ccdc180	10.078	Coiled-coil domain containing 180
Amz1	10.049	Archaelysin family metallopeptidase 1

Note: Uncharacterized genes are not listed. *Common genes between HK and LK.

In order to understand the molecular mechanisms underlying the neuroprotective effect of HSF1, we conducted bioinformatics analysis using the DEGs identified from HSF1-expressing CGNs treated with LK. Gene Ontology analysis showed, not surprisingly, that protein folding and protein processing in endoplasmic reticulum are the top two pathways regulated by HSF1 (Fig. 3B and Table S3). Some of the DEGs upregulated by HSF1 in these two pathways, including *Hsp90ab*1, *Hsph1*, *Cryab*, *Hspa4l*, and *Dnaja1*, are well known targets of HSF1. Additionally, 28 DEGs are linked to the regulation of apoptosis. Interestingly, only 7 of these 28 genes (*Hsp90ab1*, *Cryab*, *Bag3*, *Dnaja1*, *Hspb1*, *Hspa4*, *Bcap31*) encode HSPs (Table S3). Gene ontology analyses of our data also suggested that besides its well-studied role as in maintaining proteostasis, HSF1 may also regulate intracellular signal transduction, ATP binding, protein transport, and other biological processes (Fig. 3B and Table S3). As for functions in diseases, Ingenuity Pathway Analysis (IPA) indicated that 320 of the DEGs are involved in organismal injury and abnormalities, while 33 play roles in neurological diseases (Table S4). IPA analysis of the DEGs showed that the top protein network that HSF1 is associated with is cellular assembly and organization, cellular compromise and post-translational modification (Fig. 3C).

### Genes regulated by HSF1-AB in CGNs undergoing apoptosis

**Figure 4:**
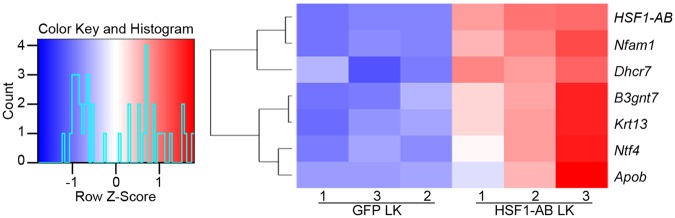
Genes regulated by HSF1-AB in LK-treated CGNs. Six genes that were regulated by HSF1-AB in LK compared to GFP LK are shown in a heat map.

In healthy neurons, expression of 13 genes was changed by HSF1-AB (Table 3). However, only 6 genes (*Nfam1*, *Dhcr7, B3gnt7*, *Krt13*, *Ntf4,* and *Apob*) were altered by HSF1-AB compared to control sample in LK (Fig. 4 and Table 4). *Nfam1* encodes a protein that functions as an immunoreceptor, which activates the NFAT-signaling pathway and cytokine production^19^. Both *Dhcr7* and *Apob* are associated with cholesterol metabolism. DHCR7 functions in conversion of 7-dehydrocholesterol to cholesterol, while ApoB is the primary apolipoprotein of chylomicrons and low density lipoproteins with cholesterol transporter activity^20–25^. Whether altered expression of *Nfam1*, *Dhcr7, Apob, B3gnt7*, and *Krt13* contributes to the regulation of neuronal survival remains to be determined. On the other hand, NTF4 is a member of the neurotrophin family of neurotrophic factors that is known to promote neuronal survival by activating the TrkB receptor tyrosine kinase^26–28^.

**Table 3:** Genes regulated by HSF1-AB in healthy CGNs.

Genes	Fold change	Protein description
Angptl1	105.013	Angiopoietin-like 1
Pik3ap1	8.274	Phosphoinositide-3-kinase adaptor protein 1, signaling adapter
Vdr	7.891	Vitamin D receptor
Trpv4	6.592	Transient receptor potential cation channel subfamily V member 4
Slc44a4	6.081	Solute carrier family 44 member 4, sodium-dependent transmembrane transport protein
Kcp	5.487	Kielin/chordin-like protein
Llgl2	5.427	Lethal Giant Larvae Homolog 2, Scribble Cell Polarity Complex Component
Klc3	5.109	Kinesin light chain 3
Cyp2d4	4.814	Cytochrome P450 family 2 subfamily d polypeptide 4
Slc16a3	4.343	Monocarboxylate transporter 4
Hck	3.640	Tyrosine-protein kinase HCK, member of the Src family of tyrosine kinases
Oas1a	2.072	2′-5′ Oligoadenylate synthetase 1 A
Uba7	1.994	Ubiquitin-like modifier-activating enzyme 7

**Table 4:** Genes regulated by HSF1-AB in apoptotic CGNs.

Genes	Fold change	Protein description
Apob	95.474	Apolipoprotein B
Nfam1	93.506	NFAT-activating protein with ITAM motif 1, receptor in immune system
Ntf4	18.585	Neurotrophin-4, member of a family of neurotrophic factors
Krt13	13.674	Keratin, type I cytoskeletal 13
Dhcr7	3.388	7-Dehydrocholesterol reductase
B3gnt7	2.714	UDP-GlcNAc:betaGal beta-1,3-N-acetylglucosaminyltransferase 7

Although the statistical criteria we used are standard for analysis of RNA-Seq data, we extended our analyses to use more relaxed statistical criteria to examine whether DEGs that could represent valid targets were missed due the previously-employed statistical cutoff (fold change >1.5, FDR < 0.05). Modest increase in DEG numbers was observed with less stringent criteria (Table S5). However, none of these DEGs provided sufficient information to identify additional enriched functional categories comparing to current criteria. With FDR < 0.05, lowering fold change cutoff from 1.5 to 1 did not identify any additional HSF1-AB targets (Table S6). With fold change >1, FDR < 0.01, we identified additional 15 HSF1-AB targets (10 in HK and 5 in LK; Table S6). The relatively modest number of genes regulated by HSF1-AB, even when very relaxed criteria are used, suggests that it is unlikely to act as a transcriptional regulator.

### Validation of RNA-Seq results using quantitative RT-PCR (QPCR)

**Figure 5:**
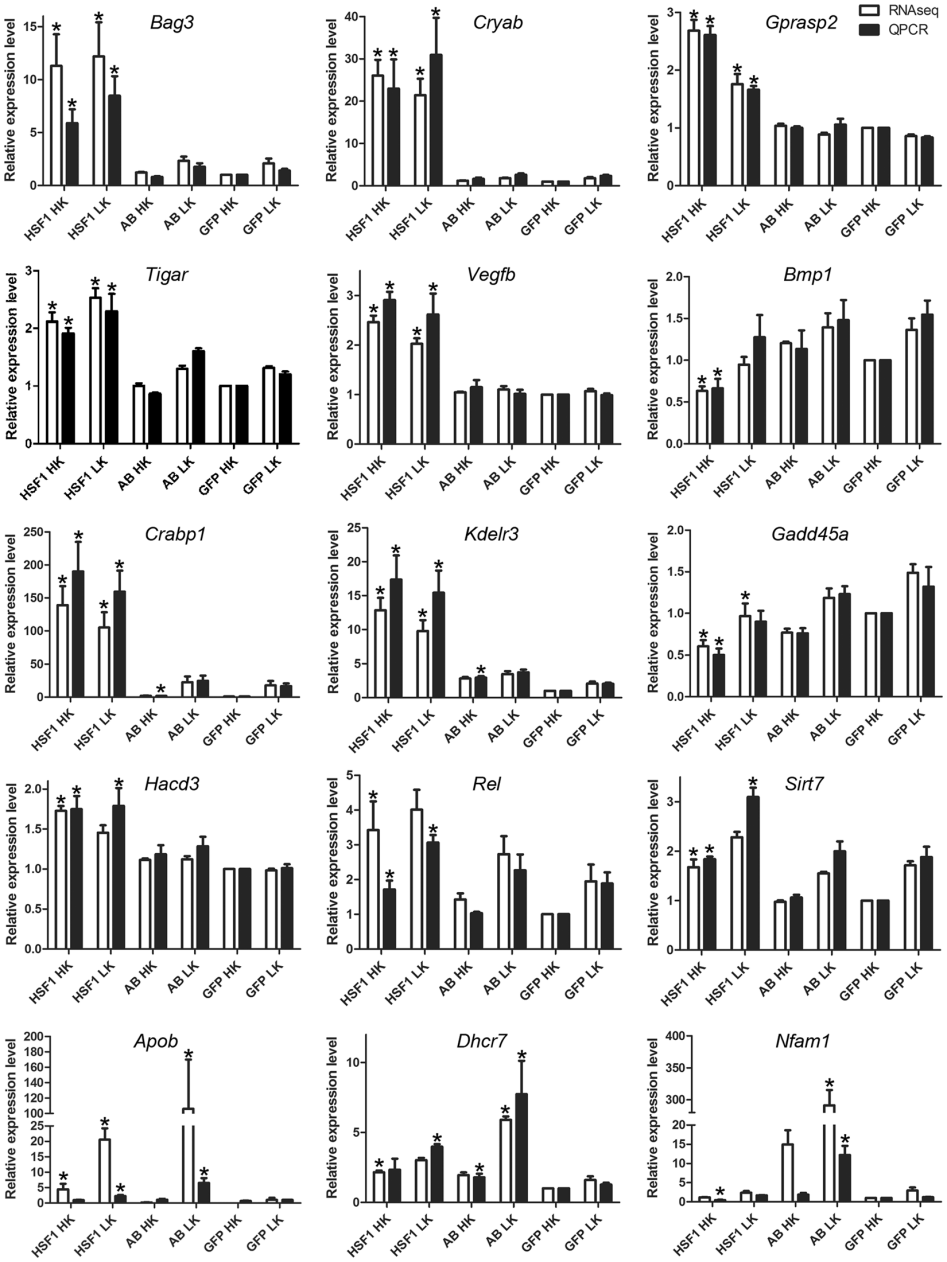
Validation of RNA-Seq results using QPCR. Fifteen DEGs, including *Bag3*, *Cryab*, *Gprasp2*, *Tigar*, *Vegfb*, *Bmp1*, *Crabp1*, *Kdelr3*, *Gadd45a*, *Hacd3*, *Rel*, *Sirt7*, *Apob*, *Dhcr7*, and *Nfam1* were selected for result validation using QPCR. General consistency in results was found between the two methods. *P < 0.05, fold change > 1.5. Analyses of at least 3 independent culture sets was performed for each gene and results displayed as mean ± SE. *P* < 0.05 is considered as statistically significant.

We selected 15 DEGs identified by RNA-Seq analysis in this study for result validation using QPCR. These DEGs were chosen because their expression levels were altered dramatically and they exert important functions in the cells. The results from the two approaches generally agreed with each other (Fig. 5). Specifically, our QPCR data confirmed that *Bag3*, *Cryab*, *Gprasp2*, *Tigar*, *Vegfb*, and *Bmp1* were regulated by HSF1, but not by HSF1-AB. RNA-Seq analysis showed that *Crabp1* and *Kdelr3* were only altered by HSF1. However, the QPCR results indicated that there were significant changes in HSF1-AB HK as well. Both RNA-Seq and QPCR indicate that *Gadd45a*, *Hacd3*, *Rel*, and *Sirt7* are DEGs altered by HSF1 in HK but their level change in HSF1 LK did not meet our statistical standards in one of the two analyses. Dramatic regulation of *Apob*, *Dhcr7*, and *Nfam1* by HSF1-AB in LK were observed using both of the technologies, and these genes were also regulated by HSF1 in HK or LK but less robustly.

### NTF4 protects CGNs from LK-induced apoptosis

One of the genes that displayed increased expression with HSF1-AB was NTF4 (neurotrophin-4), a protein with known neurotrophic effect (Table 4)^26–28^. It was possible that HSF1-AB protected CGNs by stimulating NTF4 production and release. To test this possibility we supplemented LK-medium with NTF4 and evaluated rescue of CGNs after 24 hours. NTF4 was able to reduce cell death to about 20% (Supplemental Figure S2). NTF4 expression is also stimulated by HSF1 suggesting that its protective effect could also involve a stimulation of NTF4 production (Supplemental Table S2).

## Discussion

It is well established that elevating or activating HSF1 has strong neuroprotective effects^9,29,30^. Most of these studies have been conducted using models of proteotoxic stress. In such cases it is generally believed that HSF1 stimulates production of HSPs, which then refold or degrade the abnormal protein aggregates thus alleviating proteotoxic stress^9,29,30^. We recently described that HSF1 could also protect neurons in models of neuronal death in which there is no proteotoxic stress^15^. We found that HSF1 expression was reduced in dying neurons. Moreover, forced knockdown of HSF1 expression in otherwise healthy neurons causes their demise, whereas restoring elevated HSF1 levels can protect neurons that would otherwise die^15^. We also described that HSF1-AB, a mutant form of HSF1 that cannot trimerize, bind to HSEs, or stimulate HSP expression, is just as neuroprotective as wild-type HSF1 even in models in which death involves protein aggregation^15^. This raised the possibility that like HSF1-AB, wild-type HSF1 may protect neurons through a non-canonical mechanism that does not require increased HSP expression and chaperone activity. In support of a distinct neuroprotective mechanism is the finding by the Kopito lab that while protecting against toxicity, overexpression of HSF1 or treatment with HSF1 activators does not mitigate aggregation of mutant huntingtin in a cell culture model of Huntington’s disease^31^. Other studies using methods such as ChIP-Seq and RNA-Seq have also identified non-HSP targets of HSF1 as well as genes that are transcriptionally regulated by HSF1 but lacking a HSE consensus sequence in their upstream regulatory regions^32^. Based on these findings we considered the possibility that HSF1 may regulate transcription by binding to non-HSE sequences as a monomer, perhaps in association with other transcriptional regulators. As a step towards testing this possibility we conducted transcriptome analysis of neurons overexpressing HSF1 and HSF-AB. Our analysis revealed that while HSF1 regulates the expression of a large number of genes both in healthy neurons (1,211 genes) and neurons primed to die (425 genes), only 13 genes displayed altered expression when HSF1-AB was overexpressed in healthy neurons (HK-treated). Likewise, the overexpression of HSF1-AB only altered the expression of 6 genes in dying neurons (LK-treated). Except for *Apob*, none of the other 5 genes has previously been reported to be regulated by HSF1. The modest effect of HSF1-AB on gene expression suggests that HSF-AB and HSF1 protect neurons by distinct mechanisms, and that neuroprotection by HSF1-AB is unlikely to be mediated through transcriptional regulation. In contrast to HSF1, which is exclusively nuclear in neurons, HSF1-AB also localizes to the cytoplasm. In view of the lack of significant effect on gene expression, it is possible that neuroprotection by HSF1-AB involves action in the cytoplasm. It may be noted that recent studies describe non-transcriptional effects of HSF1 involving protein-protein interaction and other mechanisms^33–36^. HSF1 has been shown to regulate TORC1 activity through sequestration of c-Jun N-terminal Kinase (JNK) by direct interaction^33,36,37^. Several studies have shown that activity of JNK, a cytosolic kinase, is necessary for neuronal death^38–42^. It is possible that HSF1-AB protects neurons through sequestration of JNK or another pro-apoptotic protein.

We performed QPCR to validate the expression of several genes. In most cases the QPCR results well matched those of RNA-Seq, although in a few cases (e.g. *Apob* and *Nfam1*) the fold-change showed differences. One gene that was found to be upregulated by HSF1 using both RNA-Seq and QPCR approaches is *Tigar* (TP53-induced glycolysis and apoptosis regulator). First identified as a p53-responsive gene with anti-apoptotic and anti-oxidative stress effects, some studies have described that TIGAR protects different types of neurons both in culture and *in vivo*^43–45^. HSF1, but not HSF1-AB, also upregulated *Vegfb* (vascular endothelial growth factor-B) in both HK and LK-treated neurons. *Vegfb* plays important roles in brain development regulating processes such as neurogenesis, neuronal migration, and axon guidance^46^. More importantly, VEGFB has strong neuroprotective effects in both cell culture and mouse models of Parkinson’s disease (PD) and amyotrophic lateral sclerosis (ALS)^46–52^. Although *Vegfb* expression was not increased by HK or LK alone, its expression was increased in both HK and LK conditions. It is possible that while not providing additional survival benefit in HK, its upregulation could contribute to neuroprotection in LK. Further investigation is required to understand if this is the case and what the underlying mechanisms might be.

In contrast to these genes, expression of another gene, *Gadd45a* (growth arrest and DNA-damage-inducible protein-a), is suppressed by HSF1 both in HK and LK. In a cell culture model of PD and excitotoxic death GADD45a promotes neuronal death, suggesting that its downregulation might contribute to HSF1-mediated neuroprotection^53,54^. *Gprasp2* (G protein-coupled receptor associated sorting protein-2), another gene upregulated by HSF1, is expressed predominantly in the central nervous system and involved in receptor trafficking and degradation^55^. Interestingly, GPRASP2 has been found to interact with huntingtin (*Htt*) and this association is enhanced by polyQ-expansion^56^. One could speculate that GPRASP2 promotes neuronal survival and that this function is impaired through interaction with polyQ-expanded *Htt*. Little is known about the function of *Sirt7* in neurons. Overexpression of SIRT7 has no effect on LK-mediated death of CGNs^57^. However, another study found that SIRT7 protects neurons against oxygen-glucose deprivation and reoxygenation-induced by suppressing p53, PUMA and Bax expression^58^. Little is known about *Kdelr3*, which is expressed in the endoplasmic reticulum, or *Hacd3*, an enzyme highly expressed in the brain and adrenal gland, both of which were also stimulated by HSF1^59–61^. In contrast to most genes that were validated by QPCR, the change in expression of a few genes, including *Shh*, *Gli1* and *Ntf4*, could not be validated by QPCR (data not shown). However, given that Ntf4 (neurotrophin-4) is a well-established neurotrophic factor^26–28^, we examined whether it could contribute to the protection of CGNs. When we supplemented LK-medium with NTF4 and evaluated rescue of CGNs after 24 hours, NTF4 was able to reduce cell death to about 21% (Figure S2). It is possible that HSF1-AB protects CGNs by directly or indirectly stimulating NTF4 production and release. NTF4 expression is also stimulated by HSF1 suggesting that its protective effect could also involve a stimulation of NTF4 production (Table S2).

Bioinformatic analysis of the HSF1-regulated genes identified 28 that are linked to the regulation of apoptosis (Table S3). Which of these DEGs are involved in the neuroprotective action of HSF1, if any, needs to be experimentally investigated. Interestingly, only 7 of these genes encode HSPs or proteins with documented chaperone activity. One of these genes is *Bag3*, an HSP70 co-chaperone, which we find to be robustly upregulated by HSF1 (Tables S1 and S2). A role for BAG3 in protecting neurons against proteotoxic stress is well-established^62–65^. *Rbm12*, the top one gene that HSF1 regulated in LK with a 107.5-fold change (Table 2), encodes a protein named RNA-binding Motif Protein 12. This gene has been linked to psychosis in recent report^66^. Some RNA-binding motif-containing genes have been suggested to play roles in the modulation of apoptosis however, *Rbm12* shows substantial sequence difference with these genes^66–68^. Whether the robust upregulation of *Rbm12* and other DEGs identified in our study contribute to the neuroprotective effect of HSF1 remains to be determined.

In this study, RNA-Seq analysis was performed using CGNs overexpressing HSF1 or HSF1-AB for 40 hours followed by LK treatment. In a limited study we extended our result validation to different protein expression time points using QPCR (Figure S3). Our results showed that, consistent with what we found at 40 hours, all of the genes examined (*Bag3*, *Tigar*, *Vegfb*, *Crabp1*, and *Rel*) could be stimulated by HSF1 but not by HSF1-AB at 32 hours and/or 48 hours. Furthermore, we determined the expression of 8 DEGs in another neuronal cell type, primary cortical neurons (Figure S4). In CGNs, 5 of these genes (*Bag3*, *Tigar*, *Vegfb*, *Crabp1*, and *Sirt7*) could be stimulated by HSF1 and the other three genes (*Apob*, *Dhcr7*, and *Nfam1*) were targets of both HSF1 and HSF1-AB. The same gene regulation pattern but with different extent of change was observed in cortical neurons-overexpressing HSF1 or HSF1-AB. Further investigation is needed to determine the common and distinct regulatory mechanisms of HSF1 in cortical neurons and CGNs.

We also compared the DEGs identified by HSF1 expression with transcriptome analysis conducted by four other labs using different tissue and non-neuronal cell types. Although a number of DEGs in our study were previously identified in these other studies, 967 out of the 1,266 HSF1-reglated genes identified in either HK or LK in our study are novel (921 novel genes in HK and 320 novel genes in LK). Solis *et al*. identified 9 genes (*Hspe*, *Hsp90ab1*, *Dnaja1*, *Dnajb1*, *Hspa1a*, *Hspa1b*, *Hspa8*, *Dedd2*, and *Hsph*) as the core mammalian *Hsf1*-dependent genes in yeast^69^. This set of 9 genes were also upregulated by heat shock in wild-type mouse embryonic fibroblasts and embryonic stem cells but suppressed in both heat-shocked *hsf1*^−/−^ cell types^69^. Among these 9 genes, *Hsp90ab1*, *Dnaja1*, *Dnajb1*, *Hspa1a*, *Hspa1b*, and *Dedd2* were found altered by HSF1 in our study. Another study identified over 1,000 genes that displayed altered expression (FDR < 0.05) upon shRNA-mediated knockdown of HSF1 compared with non-transformed control cells^32^. Only 61 of these genes were shared with our data set. Although not expansive, a much greater level of overlap was found between our list of DEGs and those found in a study by Ryno *et al*. conducted using HEK293 cells^70^. The comparison revealed 271 common DEGs, which account for 21% of the genes we identified from CGNs. The higher level of overlap with our DEGs may be because both studies employed ectopic HSF1 expression in the absence of cellular stress while the other two studies identified DEGs in cells after HSF1 depletion and/or heat shock. Using Hela cells and microarray analysis followed by RT-PCR validation, Hayashida *et al*. identified 29 novel human HSF1-regulated genes. Four genes, *Cryab*, *Nfatc2*, *Sertad4*, and *Prom2*, are also found in our data set^17^. Overall therefore, the large number of novel DEGs identified in our study (total of 967) suggests that HSF1 activates distinct pathways in postmitotic neurons than in other healthy and transformed cell types.

As a control in our RNA-Seq analysis we also included CGN cultures transduced with GFP and treated with HK and LK. In these GFP-overexpressing cultures we found 4,349 DEGs in LK compared with HK, with 2,977 genes upregulated and 1,372 downregulated. In a previous study we conducted in 2014 we described similar amount of DEGs (3,292 genes; fold change > 1.5, FDR < 0.05) in LK versus HK treated CGNs in cultures that were not manipulated to overexpress any protein^71^. A comparison of DEGs in the two studies shows a 52% overlap (1,710 DEGs in common). It deserves mention that while the previous study by Sharma *et al*. was conducted at 6 hours after HK or LK treatment^71^, in the current study RNA was isolated at 8 hours after treatment.

In conclusion, our RNA-Seq analysis conducted using primary neurons has identified a large number of novel HSF1 targets a majority of which are unlikely to be HSPs. It remains to be determined which of these DEGs contribute to the novel non-chaperone-dependent mechanism by which HSF1 protects neurons. Using Hela cells, 7 non-chaperone targets of HSF1 were identified which could inhibit polyQ aggregation^17^. This and other studies have provided strong evidence that HSF1 can act against cellular stress through mechanisms distinct from HSP stimulation^17,32^. A surprising finding of our study based on the utilization of the HSF1-AB mutant is that HSF1 can also protect neurons without trimerization but this is likely mediated through a non-transcriptional mechanism. Understanding of these novel mechanisms by which HSF1 protects neurons could lead to the development of therapeutic approaches for neurodegenerative diseases.

## Methods

### Materials

Unless otherwise specified, all chemicals and reagents were purchased from Sigma-Aldrich (St. Louis, MO). Cell culture media were purchased from Thermo Fisher Scientific (Waltham, MA).

### Generation of adenovirus

The generation of GFP-encoding adenovirus (Ad-GFP) was described previously^72^. HSF1-FLAG adenovirus (Ad-HSF1) and HSF1-AB-FLAG adenovirus (Ad-HSF1-AB) were made using ViraPower adenoviral expression system (Thermo Fisher Scientific) following manufacturer’s instruction. HSF1-Flag was cloned from a pCMV-HSF1-Flag plasmid (Addgene, Cambridge, MA; plasmid number 1932), and HSF1-AB-Flag was amplified from pCMV-AB-Flag^15^. The primers used for both constructs are as follows: Ad-HSF1 Forward, 5-GGGGACAAGTTTGTACAAAAAAGCAGGCTTCACCATGGA TCTGCCCGTGGGCCC-3, and Ad-HSF1 Reverse, 5 -GGGGACCACTTTGTACAAGAAAGCTGGGTGTTATTACTTATCGTCGTCATCCTTGTAATC-3. The PCR products were cloned into a shuttle vector pDONR221 and then recombined with a destination vector pAd/CMV/V5-DEST. The adenovirus was amplified in the HEK293A cells (ATCC, Manassas, VA) and subsequently purified using CsCl density gradient centrifugation followed by dialysis with Phosphate-Buffered Saline (PBS). The multiplicity of infection for neuronal cultures was approximately 10.

### Culturing of CGNs and adenovirus infection

CGNs were prepared as previously described^18^. Briefly, CGNs were prepared from 7-day-old Wistar rats and plated in Basal Minimal Eagle medium (BME) containing 10% fetal bovine serum (FBS), 25 mM KCl, 2 mM glutamine, and 0.2% gentamycin, in 24-well plates (1 × 10^6^ cells/well) or 60 mm dishes (1.2 × 10^7^ cells/dish). Five days later, the adenovirus was added to the CGN cultures for 2 hours and then removed. The CGNs were further cultured for 40 hours followed by high potassium (HK; BME supplemented with 25 mM KCl) or low potassium (LK; BME only) treatment for 8 hours. We chose the 40 hours expression time based on control experiments in which infection efficiency and expression of HSF1 and HSF1-AB was evaluated at various time-points after adenoviral infection. The infection rate for Ad-HSF1, Ad-HSF1-AB, and Ad-GFP in CGNs was approximately 50–60%. Cortical neurons were obtained from E17 – 19 rat embryos as previously described and plated in Neural Basal medium with B27 supplement^73,74^ Five days after plating, the adenovirus was administrated to the cultures for 2 hours and then removed. The cortical neurons were further cultured for 40 hours allowing ectopic proteins to express.

For immunocytochemistry analysis, the cells were fixed using 4% paraformaldehyde in PBS and immunocytochemistry was performed. GFP antibody (Santa Cruz Biotechnology, Dallas, TX; catalog # sc-9996) and FLAG antibody (Sigma, catalog # F1804) were utilized to detect GFP and HSF1, respectively. The secondary antibody Dylight 594 (catalog # 115-585-146) was purchased from Jackson ImmunoResearch Laboratories (West Grove, PA).

### RNA-Seq analysis

Total RNA from three independently prepared and transduced neuronal cultures was extracted using TRIzol reagent (Thermo Fisher Scientific) by standard protocol. RNA-Seq library was prepared using RNA sample preparation kit (Illumina, Cambridge, UK) following the manufacturer’s protocol. Strand-specific sequencing was performed by using a NextSeq 550 system at the Genomics and Microarray Core Facility at the University of Texas Southwestern Medical Center using default parameters.

### Differential gene expression analysis

All the RNA-Seq fastq raw reads were processed by kallisto (version 0.43.1)^75^ for quantification at both gene and isoform levels. The rat reference genome RGSC 6.0/rn6 was used together with the Ensemble Gene annotation from UCSC genome browser (http://genome.ucsc.edu/) as reference transcriptome. We also add human HSF1 Ensemble Gene annotation into the rat reference transcriptome to quantify the transgenic expression of human HSF1 gene. The estimated read counts were used in detecting DEGs by using EdgeR^76^. At least 1.5-fold change with FDR less than 0.05 were used as statistical cutoff for detecting DEGs. The MDS plot and the heatmap plots were generated by R.

### Bioinformatics

DAVID (version 6.8)^77^ was used for gene function enrichment analysis of the DEGs. The protein interaction network was generated by Ingenuity Pathway Analysis (IPA; http://www.ingenuity.com) based on experimental observations.

### PCR

cDNA was synthesized from 3 μg of RNA using the Verso cDNA Synthesis Kit (Thermo Fisher Scientific). QPCR was performed with CFX96 Real-Time System using iQ SYBR Green Supermix (BioRad, Hercules, CA) according to manufacturer’s instructions. Primers used for QPCR are as follows: *Cryab* Forward: TGGACGTGAAGCACTTCTCTC; *Cryab* Reverse: ATGAAGCCATGTTCGTCCTG; *Bag3* Forward: AAGAATGTGGCTGCAGAACC; *Bag3* Reverse: ATGGCTTCCACTTTCAGCAC; *Apob* Forward: TTGCCACAGCTGATCGAAGT; *Apob* Reverse: GTATAGCACTCCGGCTGTCC; *Crabp1* Forward: ATCAACTTCAAGGTCGGAGAGG; *Crabp1* Reverse: TCTGCGTGCAGTGAATCTTG; *Nfam1* Forward: AAGCTCGGAGAAGCCAATTG; *Nfam1* Reverse: TTTGGCAGGCTGAGTTGAAC; *Dhcr7* Forward: TTCAAAGTCCCAGCACAACG; *Dhcr7* Reverse: ACACAATGAACGGTGCGAAG; *Tigar* Forward: CCACGGGGCTTACATGAGAA; *Tigar* Reverse: ACTGATGCCAGTGTTGGGAG; *Gadd45a* Forward: GGAGTCAGCGCACCATAACT; *Gadd45a* Reverse: GGTCGTCATCTTCATCCGCA; *Kdelr3* Forward: GAACGTGTTCCGAATCCTCG;*Kdelr3* Reverse: GGTACCTGGTGGTGAAGACC; *Vegfb* Forward: GTGGTCAAACAACTCGTGCC; *Vegfb* Reverse: CATTCGGACTTGGTGTTGCC; *Bmp1* Forward: TTGGCCGACTACACCTACGA; *Bmp1* Reverse: GCAATGTCCCCAAGAAAGGC; *Gprasp2* Forward: ATTTAGCGTCCAGGCCCAAA; *Gprasp2* Reverse: AAGCTCTGCACTCTTGGCTT; *Sirt7* Forward: ACATTGTGAACCTGCAGTGGA; *Sirt7* Reverse: CTGCCACCGGTTGTAGACAG; *Rel* Forward: TTGCCATTGTTTTCAGGACGC; *Rel* Reverse: TGCGCAGGTATCTTGAAGTCCA; *Hacd3* Forward: AGTGGAAACCCTCAATGCCG; *Hacd3* Reverse: TTCCATGGTGCCGAAAACGA. Regular RT-PCR was performed with GoTaq Green Master Mix (Promega, Madison, WI). Primers used are as follows: *c-Jun* Forward: GATGGAAACGACCTTCTACG; *c-Jun* Reverse: GTTGAAGTTGCTGAGGTTGG; *HSF1* Forward: GGAAAGTGGTCCACATCGAG; *HSF1* Reverse: TTCACTCTCCCGCAGGATGG; *Actin* Forward: GAGAGGGAAATCGTGCGTGAC; *Actin* Reverse: CATCTGCTGGAAGGTGGACA.

### Western blot analysis

Cells were lysed with cell lysis buffer (20 mM Tris-HCl, pH 7.5, 150 mM NaCl, 1 mM sodium EDTA, 1 mM EGTA, 1% Triton X-100, 2.5 mM sodium pyrophosphate, 1 mM β-glycerophosphate, 1 mM Na_3_VO_4_, 1 μg/mL leupeptin, and one protease inhibitor tablet) and run on 10% SDS-PAGE gels. The samples were then transferred to PVDF membrane for Western blotting. The membrane was incubated with primary antibody (1:1000 dilution) at 4 °C overnight followed by horseradish peroxidase-conjugated secondary antibody (1:10, 000 dilution) for 1 hour at room temperature. The Goat anti-Mouse IgG, IgM secondary antibody (catalog # 31444) was purchased from Thermo Fisher Scientific. The membrane was developed with Clarity Western ECL substrate (BioRad) and imaged with ChemiDoc Touch Imaging System (BioRad).

### Statistical analysis

All experiments were independently repeated for three times. All the bar graphs were generated using GraphPad Prism 5 software (GraphPad Software, La Jolla, CA) and the results were shown as mean ± SE. *P* < 0.05 was considered as statistically significant.

## Electronic supplementary material

Supplementary Information
