# Supplementary material for: Neuroprotection by Heat Shock Factor-1 (HSF1) and Trimerization-Deficient Mutant Identifies Novel Alterations in Gene Expression
Source: Sci Rep. 2018 Nov 22;8:17255. doi: 10.1038/s41598-018-35610-1 (PMC6250741; doi:10.1038/s41598-018-35610-1)
Supplement: Supplementary file 1 — Supplementary Information [file 41598_2018_35610_MOESM1_ESM.pdf]

Neuroprotection by Heat Shock Factor-1 (HSF1) and TrimerizationDeficient Mutant Identifies Novel Alterations in Gene Expression

Zhe Qu[1], Anton Sam Crosslee Louis Sam Titus[1], Zhenyu Xuan[2], Santosh R. D’Mello[1]

Department of Biological Sciences, Southern Methodist University, Dallas, TX 75275[1] and Department of Biological Sciences, University of Texas at Dallas, Richardson, TX 75080[2]

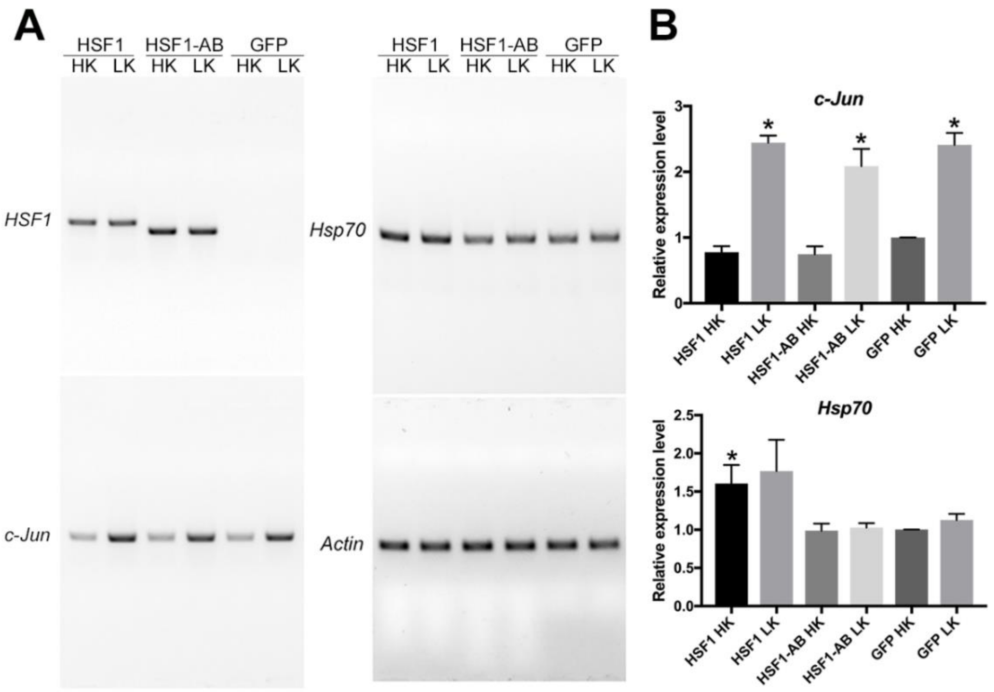

Figure S1. Marker gene expression in CGNs overexpressing HSF1 or HSF1- AB. HSF1 and HSF1-AB were overexpressed in CGNs for 4o hours followed by HK or LK treatment for 8 hours. As a step of quality control before we ran RNA-Seq analysis with the samples, we examined the expression level of two marker genes, *c-Jun* and *Hsp70*, using RT-PCR. Panel (**A**) contains uncropped DNA gel images from which RTPCR result in Figure 1B is derived. (**B**) Densitometric analysis with the images showed that expression of *c-Jun* was robustly elevated by LK treatment compared to HK (*, $P < 0.05$), while level of *Hsp70* was upregulated by wild type HSF1 in HK compared to GFP control (*, $P < 0.05$) but not by HSF1-AB. *Hsp70* level in HSF1 LK displayed increase but it was not significant. *Actin* served as an internal control.

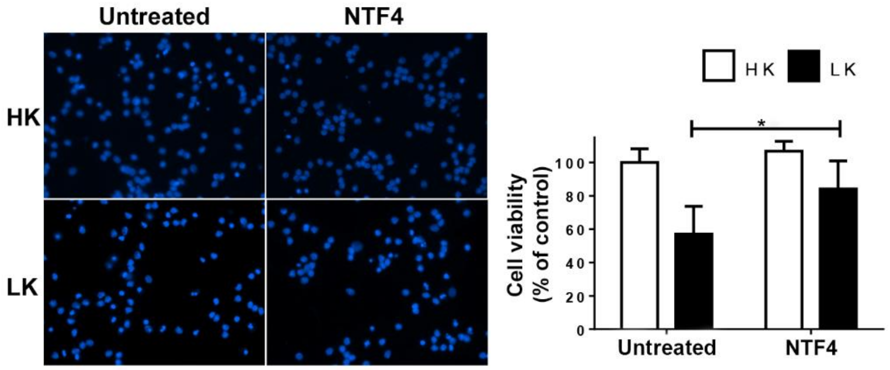

Figure S2. Effect of HSF1-AB-regulated genes in neuroprotection. CGNs were subjected to HK or LK treatment for 24 hours in the present or absent of 100 ng/mL of human NTF4 protein. Cell viability analysis by DAPI staining showed that LK treatment induced approximately 43% cell death, but NTF4 was able to restore the cell death level to about 21%. *, $P < 0.05$.

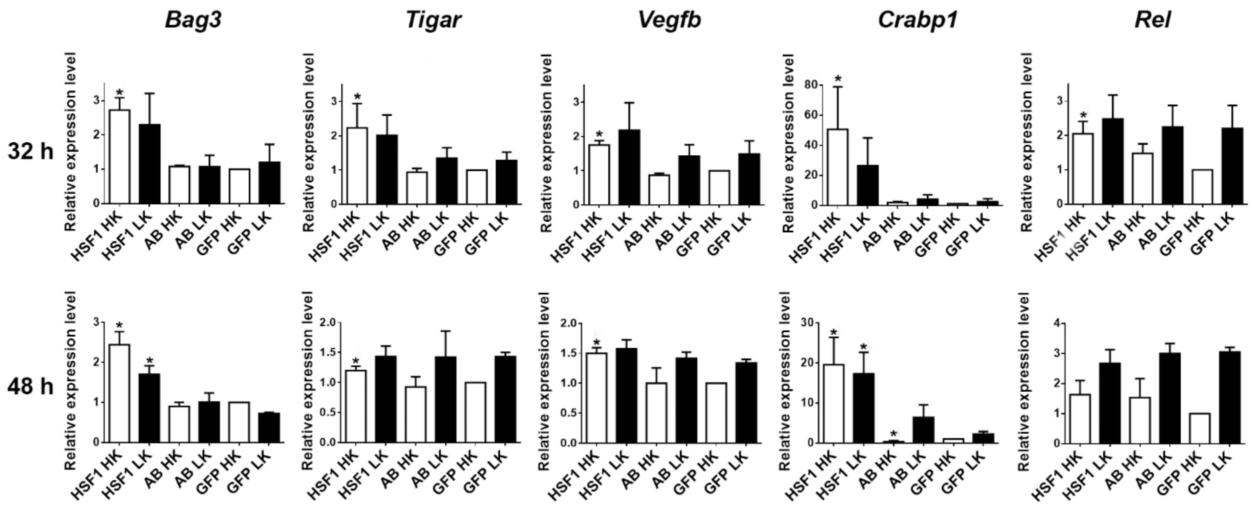

Figure S3. Level of HSF1-regulated genes at different time points. HSF1 and HSF1-AB were overexpressed in CGNs for 32 hours or 48 hours followed by LK treatment before RNA isolation. Expression level of 5 genes (*Bag3*, *Tigar*, *Vegfb*, *Crabp1*, and *Rel*) was then examined by QPCR. Statistical analysis was performed comparing HSF1/HSF1-AB HK with GFP HK and HSF1/HSF1-AB LK with GFP LK. *, $P < 0.05$. The results showed that these mRNAs were upregulated by HSF1 but not by HAF1-AB, which is in line with our findings at 40 hours. Expression of these mRNAs in HSF1 LK at 32 hours exhibited certain increase but it was not significant. In HSF1-AB HK at 48 hours, level of Crabp1 decreased compared to GFP HK, which is consistent with what we observed at 40 hours as well.

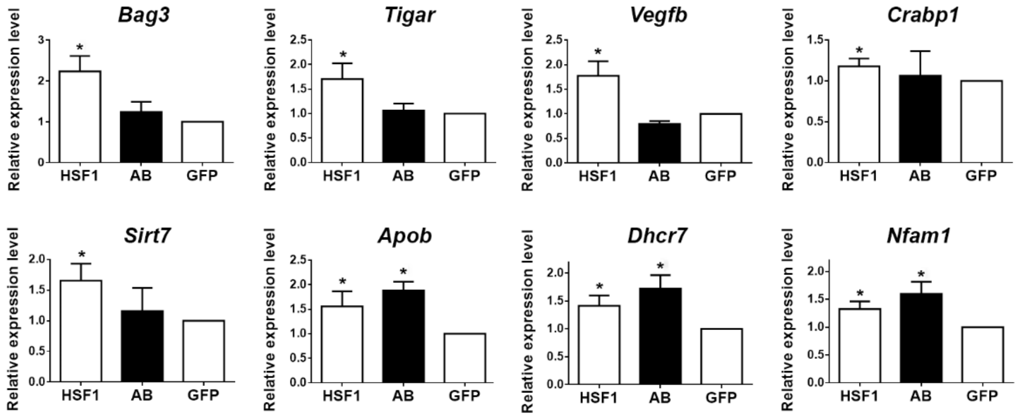

Figure S4. Expression level of HSF1/HSF1-AB-regulated genes in cortical neurons. HSF1 and HSF1-AB were overexpressed in cortical neurons for 48 hours, and then level of 8 DEGs identified in CGNs was examined by QPCR. Consistent with what we observed in CGNs, our results showed that 5 genes (*Bag3*, *Tigar*, *Vegfb*, *Crabp1*, and *Sirt7*) were stimulated by HSF1 and the other 3 genes (*Apob*, *Dhcr7*, and *Nfam1*) were targets of both HSF1 and HSF1-AB in cortical neurons. *, $P < 0.05$.

Table S1. HSF1-regulated genes in healthy CGNs

The RNA-Seq analysis was conducted using three sets of samples prepared from independent neuronal cultures infected either with Ad-HSF1, Ad-HSF1-AB, or Ad-GFP. In this table, expression level (log2-transormed) of the HSF1 targets in HK in HSF1-expressing and GFP-expressing samples is shown along with log2 [fold change] and FDR.

Table S1. HSF1-regulated genes in healthy CGNs

Genes	HSF1 HK-1	HSF1 HK-2	HSF1 HK-3	GFP HK-1	GFP HK-2	GFP HK-3	Log2 [fold change]	FDR
Slc34a2	1.974	2.510	3.026	0.000	0.000	0.024	9.269	4.42E-06
Crybb1	1.397	1.663	2.572	0.000	0.000	0.000	8.268	1.08E-03
AABR07069913.1	1.722	1.925	2.824	0.000	0.000	0.000	8.085	9.59E-04
LOC102546376	0.705	3.632	3.350	0.000	0.000	0.000	7.628	1.12E-02
Krt42	0.739	1.134	1.637	0.000	0.000	0.000	7.611	2.14E-04
AABR07016950.1	2.543	3.257	4.071	0.000	0.000	0.000	7.535	3.51E-03
AABR07016919.1	1.655	2.284	2.831	0.000	0.000	0.000	7.411	5.14E-04
NEWGENE_156225	1.597	0.000	1.530	0.000	0.000	0.000	7.290	1.38E-02
Crabp1	5.389	5.619	6.007	0.317	0.666	0.459	6.934	3.85E-07
Apob	0.050	0.101	0.212	0.000	0.000	0.000	6.811	2.93E-02
Ovol2	1.851	1.833	2.736	0.107	0.000	0.000	6.578	4.03E-04
Angptl1	0.588	0.703	0.588	0.000	0.000	0.000	6.565	1.04E-04
Nmb	6.083	6.277	6.785	0.850	1.057	0.940	6.470	2.79E-09
Plac9	2.835	3.481	4.126	0.000	0.168	0.271	6.466	1.46E-05
Kank4	3.611	3.467	4.387	0.163	0.172	0.329	6.320	1.39E-07
Myo1h	1.237	1.226	1.423	0.000	0.073	0.000	6.313	2.18E-05
Rfx6	1.685	1.693	2.798	0.041	0.050	0.082	6.260	6.24E-05
Pitx2	2.015	2.583	2.654	0.074	0.089	0.074	6.153	9.05E-07
AABR07069821.1	2.081	2.906	2.471	0.000	0.240	0.000	6.100	1.28E-04
Sox15	2.717	2.668	3.540	0.000	0.383	0.000	6.063	3.42E-05
LOC102550797	0.632	1.907	1.475	0.000	0.000	0.000	6.056	1.86E-02
Pax7	2.538	2.839	3.304	0.069	0.160	0.196	5.905	2.18E-05
Igfbpl1	4.802	5.111	6.192	0.536	0.747	1.017	5.902	9.05E-07
Nodal	0.511	0.790	1.416	0.048	0.000	0.000	5.804	2.82E-03
Tchh	3.645	4.216	4.509	0.293	0.431	0.448	5.764	2.93E-07
Mcoln2	0.128	0.211	0.460	0.000	0.000	0.000	5.673	1.03E-02
Gipc3	0.400	0.484	0.897	0.000	0.000	0.000	5.671	1.96E-03

Nipal1	0.318	0.522	0.990	0.000	0.000	0.091	5.662	2.30E-03
Zfp93	2.091	2.355	3.199	0.143	0.116	0.144	5.631	4.42E-06
Pde6b	0.682	0.638	0.959	0.000	0.000	0.043	5.608	5.34E-04
Madcam1	3.025	3.947	3.974	0.224	0.341	0.357	5.605	1.39E-07
Drd5	0.363	0.909	1.785	0.070	0.000	0.000	5.588	2.80E-03
LOC102546495	0.329	1.239	1.822	0.000	0.000	0.000	5.574	2.45E-02
Dmrt1	0.576	1.252	1.449	0.000	0.000	0.099	5.515	3.75E-03
Utf1	0.179	0.355	0.873	0.000	0.000	0.000	5.510	1.60E-02
Tmem102	1.169	1.169	1.607	0.060	0.000	0.060	5.408	1.41E-05
Kcnj15	0.509	0.982	1.618	0.000	0.000	0.079	5.407	2.01E-03
Sctr	2.080	1.920	2.627	0.054	0.242	0.054	5.402	1.27E-06
Esr2	1.579	1.889	2.709	0.047	0.111	0.227	5.311	1.13E-04
Ntf4	0.710	0.717	1.418	0.077	0.000	0.000	5.184	2.47E-03
Esr1	3.652	3.127	4.536	0.529	0.567	0.380	5.124	4.56E-05
LOC100360908	0.978	1.492	1.931	0.089	0.000	0.090	5.111	7.72E-03
Spetex-2F	1.685	2.955	3.680	0.117	0.177	0.120	5.098	1.36E-04
Pde4c	1.229	1.583	1.851	0.000	0.104	0.130	5.048	1.71E-04
Adamts19	0.683	0.954	1.391	0.000	0.057	0.071	5.047	5.39E-05
Adgrg2	3.774	3.442	4.334	0.426	0.679	0.431	5.024	1.01E-07
Cyp2s1	2.833	3.161	4.089	0.278	0.363	0.486	5.020	3.11E-06
AABR07016976.1	2.063	4.174	4.472	0.634	0.533	0.246	5.003	3.67E-04
Irx4	0.107	0.147	0.174	0.000	0.000	0.000	4.970	3.56E-02
Mfsd2b	0.062	0.344	0.281	0.000	0.000	0.000	4.965	9.15E-03
RT1-M5	1.452	2.234	2.275	0.000	0.417	0.000	4.893	2.69E-04
Vwde	0.806	1.126	1.808	0.000	0.056	0.137	4.856	1.63E-04
Anxa3	2.224	2.529	3.052	0.036	0.321	0.364	4.829	1.41E-05
Plxdc1	3.128	3.087	3.779	0.330	0.560	0.335	4.798	2.15E-07
Crhr2	1.571	1.982	3.268	0.000	0.206	0.521	4.783	8.54E-04
Cilp	0.161	0.247	0.471	0.000	0.030	0.000	4.746	5.35E-03

Sypl2	5.060	4.943	5.675	1.212	1.459	1.099	4.716	6.87E-07
Ddx43	1.726	2.703	3.105	0.083	0.287	0.387	4.682	9.81E-05
Fbxw10	0.917	1.057	1.495	0.000	0.129	0.073	4.673	1.18E-04
Rufy4	0.508	0.499	1.015	0.000	0.055	0.047	4.650	3.04E-04
Prss56	2.265	2.168	2.355	0.141	0.270	0.188	4.648	9.05E-07
Cryab	7.471	7.805	7.499	2.670	3.600	3.023	4.629	9.67E-08
Amz1	3.436	3.512	4.028	0.373	0.775	0.618	4.553	3.85E-07
Clec14a	1.006	1.420	1.988	0.075	0.091	0.149	4.542	1.04E-04
Prss16	1.294	1.658	1.838	0.048	0.113	0.185	4.542	1.08E-04
Apoh	1.632	1.532	1.901	0.112	0.135	0.114	4.538	1.59E-04
Alx3	0.196	0.137	0.236	0.000	0.000	0.000	4.531	1.85E-02
Fezf2	0.605	0.879	1.445	0.000	0.063	0.103	4.522	2.34E-03
Vwf	1.903	2.037	2.593	0.220	0.163	0.233	4.516	1.12E-06
Comp	3.593	3.735	4.196	0.708	0.821	1.190	4.512	2.04E-06
Acvrl1	1.445	1.895	2.761	0.127	0.150	0.314	4.476	3.26E-05
Kremen2	0.213	0.369	0.698	0.000	0.000	0.048	4.461	3.55E-03
AABR07044980.1	0.948	0.848	1.644	0.053	0.123	0.053	4.447	1.06E-03
Endou	0.353	0.322	0.449	0.000	0.049	0.000	4.439	1.46E-02
LOC108351043	2.104	1.992	3.274	0.000	0.298	0.471	4.434	9.35E-04
Chrnd	0.876	0.717	0.782	0.000	0.074	0.063	4.345	1.39E-03
Lyzl4	1.456	2.054	2.445	0.000	0.515	0.000	4.335	1.70E-03
Fam110d	2.818	3.037	4.067	0.221	0.549	0.835	4.328	4.60E-05
Lad1	2.182	2.642	3.177	0.045	0.383	0.595	4.297	9.93E-05
Krt13	2.509	2.823	3.171	0.194	0.605	0.367	4.296	2.40E-04
Adcy7	3.373	3.577	4.403	0.561	0.785	0.976	4.206	3.11E-06
Krt1	0.757	1.615	2.180	0.138	0.057	0.230	4.200	6.51E-04
Tspear	0.726	1.324	1.292	0.000	0.072	0.176	4.188	3.64E-04
Acp5	3.628	3.650	4.464	0.694	0.489	1.319	4.178	6.63E-06
Dsg2	2.038	2.413	3.372	0.217	0.319	0.595	4.171	1.64E-05

Alx4	0.499	1.207	1.719	0.000	0.066	0.212	4.138	3.88E-03
Casq2	0.482	0.221	0.352	0.000	0.059	0.000	4.122	8.99E-03
Myh7	0.444	0.246	0.525	0.030	0.024	0.020	4.118	2.21E-04
Gjb2	1.105	1.525	2.204	0.096	0.169	0.232	4.106	1.64E-05
RGD1562660	3.214	3.481	4.163	1.343	0.000	0.000	4.095	1.74E-02
Matn4	3.241	3.607	4.163	0.232	0.670	1.262	4.086	9.30E-05
Ccdc180	0.444	0.675	0.803	0.072	0.059	0.000	4.085	2.12E-03
Papln	0.938	0.895	1.391	0.073	0.108	0.092	4.074	8.00E-05
Ggt5	4.096	4.643	5.420	0.294	1.622	0.747	4.069	1.50E-04
Ankrd34c	0.371	1.251	0.442	0.000	0.115	0.049	4.062	1.09E-02
Pcdh12	0.467	1.128	1.107	0.019	0.132	0.076	4.061	3.64E-04
AABR07069837.1	1.410	1.709	1.702	0.000	0.397	0.000	4.058	4.91E-03
Npas1	1.652	2.242	2.524	0.090	0.466	0.255	4.052	3.86E-05
Zfp185	3.568	3.766	4.843	1.067	1.011	0.932	4.037	3.11E-06
Zfp663	1.822	1.886	2.378	0.131	0.234	0.371	4.033	1.49E-04
Bmpr1b	2.130	2.223	3.090	0.124	0.630	0.352	4.008	5.46E-04
Dpy19l2	0.224	0.290	0.233	0.000	0.044	0.000	3.989	3.40E-02
Pik3ap1	1.289	1.610	1.808	0.118	0.150	0.265	3.973	8.61E-05
Egfem1	1.355	1.628	2.253	0.131	0.231	0.257	3.946	7.62E-05
Kcng2	1.971	1.844	2.815	0.188	0.457	0.313	3.907	4.42E-06
Npy1r	1.671	1.792	2.052	0.375	0.105	0.386	3.898	1.79E-05
Dmrt2	0.242	0.392	0.773	0.000	0.000	0.090	3.891	6.55E-03
Kcnk4	2.670	2.834	3.339	0.437	0.357	0.774	3.885	4.38E-05
Bank1	0.986	1.063	0.968	0.135	0.311	0.260	3.875	4.21E-03
Tmem106a	3.286	3.738	4.229	0.461	1.091	1.103	3.874	8.58E-05
Gli1	0.349	0.476	0.756	0.068	0.000	0.046	3.874	9.19E-04
Calca	4.787	5.074	5.927	1.175	2.282	2.196	3.865	4.68E-06
Gsdmd	5.336	5.493	6.127	1.416	2.374	2.569	3.844	1.62E-05
Klrg2	1.385	1.347	1.791	0.000	0.274	0.234	3.826	2.32E-03

Gng11	2.328	2.649	3.054	0.167	0.803	0.393	3.819	1.28E-04
Glt8d2	2.370	2.499	2.983	0.542	0.412	0.351	3.814	6.33E-05
Plbd1	0.951	1.119	1.256	0.000	0.075	0.239	3.810	8.77E-04
Capn8	1.792	2.560	2.188	0.099	0.462	0.423	3.803	5.34E-04
Tex33	0.473	1.032	1.175	0.000	0.000	0.185	3.801	9.90E-03
Flt4	0.701	1.330	1.424	0.017	0.098	0.250	3.792	4.26E-04
Popdc3	2.473	2.706	3.306	0.486	0.635	0.381	3.788	1.90E-05
Atp2b4	2.020	2.363	3.138	0.195	0.586	0.530	3.785	5.85E-05
Rspo2	2.126	2.346	3.134	0.384	0.399	0.545	3.775	2.79E-05
Cdh3	1.411	1.382	2.357	0.027	0.214	0.449	3.744	4.10E-04
Baiap2l1	2.858	2.898	3.589	0.683	0.841	0.478	3.729	8.66E-06
Mcf2	2.127	2.594	3.158	0.446	0.615	0.420	3.718	8.66E-06
Btn1a1	0.643	0.759	1.022	0.071	0.126	0.037	3.708	2.14E-04
LOC100912034	0.409	0.873	0.470	0.000	0.000	0.134	3.704	4.93E-02
Grb7	1.294	1.822	1.784	0.155	0.376	0.127	3.671	6.34E-06
Kcns3	1.203	1.647	2.547	0.143	0.322	0.358	3.659	2.59E-04
Fgfr4	1.139	1.164	2.589	0.219	0.285	0.243	3.657	1.07E-03
Hspb1	7.275	7.587	8.238	3.341	4.315	4.638	3.631	1.79E-05
Wdfy4	0.183	0.437	0.267	0.018	0.000	0.055	3.621	1.63E-03
LOC102554838	2.099	2.174	2.959	0.404	0.488	0.414	3.603	3.67E-04
Gpr179	0.258	0.205	0.620	0.000	0.032	0.110	3.602	1.91E-02
Tgm7l1	0.467	0.958	1.075	0.050	0.118	0.101	3.539	2.15E-03
Melk	3.264	3.665	4.229	0.651	1.329	1.160	3.532	1.51E-05
Kdelr3	5.999	5.882	7.036	2.305	2.815	3.464	3.529	8.66E-06
Cstl1	1.330	1.985	2.304	0.264	0.582	0.000	3.529	5.36E-03
Sash3	0.481	0.714	0.924	0.042	0.000	0.163	3.523	6.66E-03
RGD1559714	0.403	0.627	0.829	0.000	0.181	0.000	3.523	3.88E-03
AABR07068094.2	0.850	1.595	1.797	0.000	0.474	0.119	3.512	1.89E-02
Foxa2	0.248	0.252	0.652	0.000	0.055	0.047	3.509	1.44E-02

Lamc2	3.867	4.091	4.162	0.966	1.275	1.409	3.493	9.05E-07
Upk1b	0.736	1.614	1.836	0.119	0.074	0.387	3.475	5.40E-03
Tex40	2.404	2.722	3.250	0.000	0.744	0.981	3.469	2.22E-03
Tmc4	1.259	1.303	1.855	0.124	0.238	0.276	3.468	4.91E-04
Trim16	2.708	2.841	3.530	0.714	0.771	0.705	3.468	1.83E-06
Tacr2	0.676	0.835	1.367	0.068	0.121	0.166	3.453	1.09E-03
Vav1	1.258	1.342	1.693	0.088	0.106	0.401	3.448	2.35E-03
Cdh23	1.059	0.957	1.467	0.109	0.190	0.173	3.440	9.29E-05
Scnn1b	0.370	0.626	0.797	0.000	0.125	0.072	3.425	7.72E-03
Muc5b	0.112	0.229	0.334	0.008	0.036	0.023	3.407	1.65E-03
Calr4	2.917	3.140	4.087	0.519	1.320	1.056	3.405	2.39E-04
Slco1c1	1.513	1.641	1.585	0.162	0.147	0.412	3.397	2.59E-04
Klc3	2.626	2.746	3.246	0.630	0.808	0.595	3.393	5.08E-06
Znrf4	0.278	1.163	1.560	0.077	0.092	0.224	3.389	2.57E-03
Acan	0.164	0.225	0.655	0.015	0.070	0.028	3.382	1.55E-03
Pzp	0.092	0.524	0.605	0.025	0.000	0.097	3.372	1.56E-02
Shh	0.297	0.589	0.805	0.056	0.000	0.111	3.366	3.02E-03
Smpdl3b	3.367	3.508	4.275	0.522	1.425	1.397	3.356	2.19E-05
Pgr	1.008	1.159	1.658	0.031	0.303	0.232	3.355	3.37E-04
Card14	1.538	1.451	1.980	0.225	0.402	0.199	3.355	6.69E-05
AC139642.1	2.360	2.248	3.218	0.715	0.132	0.805	3.347	1.32E-03
LOC689757	0.716	1.248	1.656	0.130	0.229	0.068	3.327	1.60E-02
Ptgis	1.001	1.368	1.799	0.070	0.163	0.433	3.322	8.64E-04
Otop3	1.815	2.349	2.465	0.573	0.432	0.299	3.321	6.50E-05
Wdr86	1.762	1.412	2.306	0.314	0.370	0.320	3.306	9.27E-05
C1ql2	2.300	2.661	3.673	0.602	0.896	0.754	3.304	1.35E-04
Bag3	5.458	5.637	6.017	1.803	2.843	3.053	3.303	8.74E-06
Bik	3.065	2.825	3.846	0.578	1.184	0.945	3.302	9.04E-05
B4galnt3	0.572	0.631	0.868	0.000	0.160	0.102	3.276	5.17E-04

Foxg1	0.170	0.236	0.414	0.000	0.044	0.037	3.273	9.62E-03
Fam83a	1.672	1.957	2.782	0.344	0.423	0.580	3.271	1.59E-04
Sh3bgr	5.781	5.506	5.668	2.744	3.051	1.786	3.262	5.33E-04
Edaradd	2.247	2.510	3.421	0.600	0.525	0.922	3.256	1.29E-03
Btla	0.819	1.348	1.633	0.193	0.268	0.069	3.255	5.01E-03
Scel	0.526	0.663	1.296	0.049	0.060	0.224	3.252	1.12E-02
Plod1	5.487	5.908	6.191	1.965	3.142	3.193	3.228	4.38E-05
Ly6l	2.316	2.674	3.329	0.271	0.640	0.951	3.223	3.69E-04
Tulp1	0.613	0.821	1.103	0.000	0.000	0.309	3.221	2.59E-02
Nlrp6	2.943	3.631	4.276	0.600	1.304	1.567	3.212	1.70E-03
Mypn	0.191	0.251	0.374	0.000	0.051	0.040	3.211	8.29E-03
Tmem37	1.218	1.676	2.047	0.216	0.398	0.282	3.210	2.18E-04
Mro	3.237	3.299	4.517	0.690	1.991	0.954	3.192	6.48E-04
Scd	3.752	4.047	4.739	1.434	1.685	1.570	3.173	7.08E-06
Slc9a3	0.501	0.496	0.865	0.057	0.134	0.058	3.172	4.51E-04
Cck	1.835	2.695	3.109	0.224	0.769	0.883	3.168	5.00E-03
Wnk4	1.020	1.363	1.939	0.216	0.388	0.221	3.132	6.31E-05
Tle6	1.699	1.719	2.439	0.100	0.283	0.792	3.125	1.54E-03
Thbs4	2.047	2.316	2.647	0.401	0.640	0.663	3.098	1.75E-05
Nlrp10	0.065	0.096	0.498	0.000	0.041	0.035	3.088	3.11E-02
Cldn10	3.442	3.410	4.365	1.162	1.397	1.449	3.083	4.67E-05
Dnai1	0.966	1.702	1.676	0.141	0.414	0.274	3.065	1.34E-03
Myo5c	1.988	2.633	2.506	0.299	0.946	0.528	3.043	4.76E-04
Esrp2	0.434	0.289	0.631	0.025	0.059	0.098	3.039	3.69E-04
Fhl3	3.234	3.697	4.493	1.039	1.514	1.641	3.036	3.74E-05
Ano5	1.137	1.159	1.252	0.188	0.173	0.277	3.034	1.22E-03
Tekt4	0.334	1.020	1.231	0.221	0.093	0.078	3.029	1.14E-02
Agmat	0.863	1.072	1.349	0.067	0.356	0.131	3.016	2.32E-03
Lrrc3c	2.033	2.231	2.792	0.268	0.573	0.876	3.010	1.91E-04

Cfap57	0.427	1.319	1.655	0.129	0.466	0.107	2.998	3.62E-03
Ptpro	6.099	6.021	6.480	3.273	2.925	3.739	2.987	1.18E-05
LOC100910996	6.359	6.243	6.756	3.712	3.478	3.538	2.984	1.83E-06
Chrna5	2.373	2.633	3.438	0.621	0.979	0.929	2.982	1.94E-03
Arhgef26	1.758	1.901	2.808	0.184	0.817	0.616	2.974	9.59E-04
Tox2	0.638	0.858	1.322	0.047	0.161	0.259	2.972	1.58E-03
Angpt4	0.813	0.947	0.794	0.172	0.000	0.214	2.972	5.00E-03
LOC679342	3.331	3.177	4.176	0.853	1.517	1.462	2.970	6.84E-05
Dmkn	1.519	2.121	2.688	0.463	0.863	0.750	2.964	8.00E-05
Gata6	0.501	0.739	0.885	0.051	0.176	0.102	2.963	3.66E-03
Mcam	6.448	6.335	7.044	3.221	3.678	4.239	2.962	3.85E-07
Hs3st2	2.547	2.574	3.388	0.479	0.901	1.142	2.959	1.18E-04
Procr	1.480	2.295	2.508	0.154	0.493	0.852	2.957	1.84E-03
Adgrd1	0.447	0.560	1.133	0.072	0.166	0.107	2.955	2.53E-02
Gdf6	0.968	1.036	1.777	0.031	0.450	0.260	2.951	6.09E-03
Il12rb2	2.007	2.027	2.291	0.581	0.465	0.481	2.940	1.60E-04
Pla2g3	0.473	0.619	0.968	0.077	0.092	0.150	2.939	2.94E-03
St6galnac2	0.297	0.865	1.234	0.111	0.000	0.265	2.923	1.57E-02
Cela1	0.640	0.704	1.232	0.100	0.000	0.287	2.920	7.13E-03
Ikzf3	0.478	0.491	0.751	0.000	0.116	0.143	2.919	4.86E-03
Mum1l1	2.664	2.677	3.212	0.685	0.856	1.086	2.919	4.74E-06
Stox1	0.703	0.798	1.317	0.143	0.172	0.180	2.917	2.34E-03
Phf11b	3.851	3.839	4.552	1.318	1.595	2.013	2.910	6.09E-05
Drd2	3.034	3.264	4.297	0.700	1.473	1.685	2.901	7.45E-04
Slc43a1	0.964	1.343	2.086	0.215	0.194	0.530	2.857	8.21E-04
AABR07050283.2	0.671	1.410	1.627	0.145	0.000	0.506	2.856	2.36E-02
Wisp2	1.579	1.734	2.307	0.380	0.396	0.582	2.853	2.05E-03
NEWGENE_2724	0.809	1.480	1.753	0.205	0.180	0.447	2.847	4.42E-04
Atp8b3	0.605	0.660	0.881	0.045	0.277	0.046	2.845	1.21E-03

RT1-DMa	5.335	5.508	6.095	2.818	3.207	2.880	2.843	6.63E-06
Glp1r	0.315	0.485	0.584	0.081	0.072	0.034	2.839	2.95E-02
Slc4a1	0.356	0.648	0.694	0.000	0.132	0.150	2.825	3.46E-02
AABR07043276.1	3.698	4.025	4.511	1.579	1.608	1.913	2.821	6.10E-04
Stab1	0.102	0.384	0.200	0.024	0.029	0.049	2.816	1.48E-02
Unc45b	0.514	0.280	0.300	0.031	0.145	0.000	2.805	2.24E-02
Cd40	0.741	1.368	1.391	0.000	0.188	0.501	2.764	2.17E-02
Trpv4	0.361	0.434	0.785	0.056	0.067	0.138	2.761	4.51E-04
Krt7	3.545	4.009	4.133	1.318	1.818	1.831	2.757	2.70E-05
Cox6b2	3.373	3.540	3.497	1.367	1.157	1.355	2.754	2.23E-03
Plekhg3	6.626	6.872	7.022	3.917	4.351	4.188	2.751	6.64E-08
Ankrd1	0.713	0.991	1.044	0.071	0.318	0.143	2.744	9.64E-03
Pmaip1	5.295	5.681	6.255	2.391	3.543	3.473	2.744	4.60E-05
Slc30a2	2.176	2.213	2.638	0.504	0.966	0.563	2.743	5.46E-06
RGD1311892	0.902	1.030	1.522	0.177	0.272	0.284	2.730	1.04E-03
Klhl4	1.772	1.727	1.679	0.318	0.657	0.330	2.718	1.97E-04
Fam20a	0.723	0.945	1.049	0.000	0.273	0.260	2.716	3.08E-03
Aldh1a2	2.323	2.366	2.602	0.593	0.478	1.074	2.711	8.87E-04
Arhgef16	0.100	0.160	0.355	0.000	0.075	0.022	2.709	3.73E-02
Lmod3	0.966	0.896	1.167	0.296	0.189	0.108	2.707	5.57E-03
Csf2rb	0.785	1.146	1.644	0.135	0.356	0.305	2.699	5.02E-03
Sbspon	1.329	1.212	2.382	0.100	0.592	0.575	2.696	5.48E-03
Aox1	4.295	4.600	4.540	1.297	2.249	2.263	2.687	6.87E-04
Atg9b	0.242	0.351	0.410	0.091	0.000	0.061	2.680	9.34E-03
Aim1l	0.103	0.228	0.107	0.031	0.037	0.000	2.672	2.78E-02
Phf11	2.518	2.494	2.556	0.709	0.870	0.848	2.659	4.00E-04
Rem1	0.919	0.967	1.393	0.172	0.071	0.425	2.658	8.87E-03
LOC685680	0.546	1.041	1.754	0.094	0.320	0.355	2.650	1.43E-02
Dennd2d	0.320	0.553	0.637	0.061	0.144	0.062	2.648	3.44E-03

Dgkb	2.793	2.628	3.598	0.994	1.106	1.281	2.638	1.25E-05
LOC102547811	3.082	3.365	3.720	1.204	1.215	1.578	2.620	1.01E-03
Lypd3	0.397	0.494	0.526	0.000	0.070	0.171	2.620	2.15E-02
Dgkk	0.492	1.107	1.711	0.146	0.306	0.022	2.615	9.67E-03
LOC102551901	4.907	5.096	4.621	2.675	2.800	1.791	2.612	3.36E-04
LOC497899	2.594	3.266	3.811	0.905	1.641	1.249	2.609	1.24E-04
Gsdma	3.687	3.698	3.959	1.440	1.780	1.660	2.603	9.05E-07
Dnajc22	2.482	2.822	3.205	0.598	1.157	1.268	2.586	1.19E-04
Adgrg6	3.029	3.253	4.013	0.834	1.677	1.707	2.581	3.66E-03
Tjp3	0.874	1.251	1.479	0.179	0.278	0.395	2.581	4.21E-04
Irf8	0.493	1.370	1.810	0.135	0.272	0.515	2.575	1.50E-02
Mc4r	0.495	0.415	0.761	0.060	0.073	0.176	2.570	1.83E-02
Cngb1	1.155	1.128	1.987	0.112	0.219	0.719	2.569	6.49E-03
Fam83g	1.662	2.260	2.544	0.189	0.919	0.856	2.567	1.71E-03
Pik3r6	0.569	1.038	0.845	0.178	0.308	0.032	2.559	4.15E-03
Fam126a	4.664	4.750	5.509	2.377	2.764	2.847	2.549	3.45E-04
Gpr149	2.124	2.274	2.954	0.473	1.052	0.970	2.542	1.65E-04
Map3k6	2.664	2.907	3.447	0.949	1.142	1.365	2.538	2.04E-06
Hmgn5b	4.262	4.910	5.732	1.971	2.241	3.611	2.528	4.13E-03
Tmem54	0.835	0.898	1.726	0.381	0.276	0.174	2.527	1.07E-03
Fosb	3.314	3.653	4.159	1.967	1.539	1.303	2.527	5.85E-05
Nckap1l	0.836	1.325	1.210	0.223	0.206	0.425	2.519	7.89E-04
Gipr	2.128	2.205	2.428	0.469	0.894	0.815	2.513	9.78E-06
Prss12	1.035	0.754	1.174	0.190	0.227	0.268	2.511	8.37E-04
AABR07060473.1	1.991	2.094	2.527	0.897	0.842	0.306	2.510	7.34E-03
Cyp2d4	0.953	1.453	1.440	0.261	0.249	0.440	2.508	1.91E-04
Igsf1	1.495	1.493	1.679	0.499	0.525	0.203	2.506	1.08E-03
Sec31b	1.246	1.559	1.396	0.228	0.461	0.413	2.505	2.93E-05
Asb2	0.532	1.036	1.538	0.137	0.399	0.225	2.502	2.32E-03

Asic2	4.138	4.323	4.669	1.976	2.398	2.154	2.487	1.69E-06
Prokr1	0.246	0.305	0.591	0.068	0.081	0.069	2.477	1.61E-02
Tnnt1	1.093	1.880	1.528	0.155	0.591	0.444	2.471	7.38E-03
Tcf7l1	2.576	3.117	3.769	0.837	1.518	1.533	2.466	7.94E-04
Trpc6	2.707	3.076	3.535	1.004	1.371	1.427	2.464	4.10E-04
Sult1a1	1.649	2.202	2.481	0.218	0.838	0.942	2.464	3.20E-03
Pmel	2.388	3.110	3.586	0.614	0.978	1.878	2.464	6.43E-03
Phldb3	0.931	0.696	1.155	0.104	0.442	0.106	2.447	7.96E-03
Fam46c	1.099	1.161	1.282	0.106	0.353	0.433	2.442	8.77E-03
Greb1	2.444	2.980	2.434	0.710	1.250	0.940	2.441	1.12E-04
AABR07073181.1	3.090	3.181	3.754	0.695	1.511	1.924	2.440	1.50E-02
Prokr2	1.456	1.817	1.103	0.318	0.666	0.244	2.433	5.90E-03
Hspa1a	5.023	5.090	5.837	2.362	1.459	4.137	2.429	3.11E-02
Thrb	3.593	3.570	4.583	1.842	2.033	1.743	2.425	2.57E-05
LOC103690141	2.009	2.044	3.083	0.698	1.151	0.687	2.419	4.33E-04
Faim2	5.566	5.504	5.172	3.219	3.421	2.673	2.413	7.35E-04
Cldn3	0.175	0.285	0.610	0.063	0.075	0.064	2.413	2.00E-02
Tmem173	3.322	3.841	4.351	1.251	2.305	1.811	2.412	4.49E-04
Gpr101	0.712	0.653	1.343	0.187	0.413	0.050	2.410	1.86E-02
Clec2d2	0.977	1.176	1.762	0.149	0.399	0.537	2.404	2.51E-03
Cmtm8	1.287	1.356	1.473	0.188	0.501	0.429	2.400	6.24E-04
Gpr160	1.018	1.107	1.468	0.079	0.565	0.296	2.396	3.26E-03
Upb1	1.263	1.986	1.725	0.102	0.774	0.604	2.378	3.44E-03
Batf	0.531	1.148	1.073	0.000	0.155	0.465	2.376	3.37E-02
Rp1l1	0.250	0.182	0.343	0.016	0.059	0.082	2.374	3.31E-02
Lyve1	0.893	1.613	1.373	0.322	0.545	0.215	2.370	6.60E-03
Il25	0.893	0.909	1.202	0.291	0.342	0.105	2.360	9.84E-03
Cntnap2	6.754	6.735	7.020	5.280	4.844	2.702	2.358	2.93E-03
Tmem255a	3.763	3.776	4.042	1.747	1.834	2.010	2.356	1.64E-05

Pitx3	1.129	1.745	2.330	0.395	0.456	0.792	2.355	8.17E-03
Fndc3c1	1.201	1.054	2.080	0.307	0.555	0.494	2.349	3.10E-02
Mb21d1	1.371	1.707	2.476	0.259	0.570	0.958	2.348	3.44E-03
Clnk	1.101	1.409	1.598	0.206	0.456	0.618	2.348	2.89E-03
Sytl3	0.569	0.736	0.703	0.147	0.120	0.197	2.324	4.10E-04
Traf3ip2	2.412	3.058	3.322	0.634	1.471	1.498	2.317	9.54E-04
Eya2	2.734	3.478	4.043	0.963	1.516	2.116	2.307	3.09E-03
Parp3	4.076	4.698	4.949	1.817	2.640	2.940	2.301	1.04E-04
Cacna1h	1.226	1.466	2.203	0.366	0.506	0.706	2.301	4.34E-04
Ccdc158	2.199	2.929	3.357	0.885	1.236	1.458	2.299	2.24E-04
Hyal1	3.341	3.996	3.971	1.323	2.228	1.935	2.290	7.85E-03
Pde11a	1.183	1.111	1.812	0.343	0.709	0.691	2.273	8.11E-03
Atoh1	1.085	1.731	2.301	0.455	0.578	0.707	2.262	1.08E-03
Igsf9	2.061	2.152	2.971	0.705	1.143	0.958	2.260	5.96E-04
Pram1	0.542	0.488	0.734	0.167	0.135	0.112	2.257	8.96E-03
Arhgef19	1.189	1.177	1.597	0.184	0.426	0.564	2.255	8.64E-04
LOC100910419	0.332	0.980	0.797	0.125	0.149	0.241	2.244	1.64E-02
Arhgap25	0.421	0.524	1.297	0.160	0.156	0.282	2.243	1.12E-02
Fam110c	0.239	0.432	0.429	0.113	0.093	0.039	2.239	1.34E-02
Cyp4v3	3.511	4.279	4.437	1.095	2.354	2.705	2.238	2.64E-03
AABR07043358.1	4.563	4.997	5.375	2.570	3.140	3.102	2.226	5.14E-04
AABR07015687.1	1.341	1.644	1.773	0.000	0.624	0.828	2.226	2.11E-02
Crispld2	4.576	5.064	5.286	2.264	3.261	3.523	2.224	1.05E-04
Grm2	2.272	2.146	3.310	0.839	1.122	1.293	2.222	3.24E-04
Mreg	3.769	3.907	4.109	1.677	2.220	1.948	2.216	1.13E-06
Gpr132	0.625	0.999	1.394	0.227	0.226	0.396	2.214	6.31E-03
Susd5	0.581	0.765	0.874	0.162	0.193	0.216	2.207	3.60E-02
Trpv1	1.070	1.023	1.565	0.309	0.342	0.474	2.206	8.64E-04
Rab26	2.510	2.739	3.369	0.914	1.390	1.472	2.202	8.33E-05

Lsmem2	2.188	2.662	2.447	0.827	1.160	0.907	2.194	1.93E-04
Rbpms	3.160	3.952	4.764	0.969	2.238	2.520	2.187	1.49E-02
Six3	0.533	0.956	1.646	0.213	0.138	0.479	2.172	1.89E-02
Ltbp4	1.682	1.830	2.350	0.482	0.785	0.896	2.171	1.91E-04
Tmem45b	0.621	0.568	0.599	0.069	0.202	0.201	2.158	2.14E-02
Ccdc148	1.393	1.269	1.781	0.377	0.692	0.728	2.152	4.90E-04
AABR07000740.1	7.960	8.121	8.497	6.017	5.869	6.321	2.141	1.09E-04
Nod2	0.144	0.681	0.611	0.054	0.107	0.143	2.140	4.56E-02
Epha10	2.609	2.820	3.470	1.172	1.506	1.259	2.139	1.93E-04
Aloxe3	5.280	5.324	6.059	3.555	3.803	3.195	2.130	8.66E-06
Wnt16	1.523	1.840	2.036	0.292	0.674	0.941	2.128	5.87E-03
Slc35g1	1.437	1.474	2.161	0.543	0.548	0.698	2.127	1.36E-02
Tnfsf9	0.722	1.334	1.600	0.391	0.289	0.459	2.126	1.55E-02
Mybpc2	1.553	1.969	1.996	0.601	0.952	0.410	2.124	8.21E-04
Ggh	6.571	6.561	7.070	4.528	4.917	4.561	2.121	3.85E-07
Hmgn5	5.739	4.998	5.791	3.626	3.868	2.992	2.117	1.07E-03
Prss35	1.676	1.670	2.247	0.492	0.754	0.844	2.112	8.00E-05
Morc4	4.014	4.291	4.690	2.365	2.640	2.619	2.107	1.05E-04
Slc9a2	1.714	1.986	2.064	0.769	0.913	0.415	2.107	9.19E-04
Hspb2	2.194	2.298	2.723	1.132	0.558	1.132	2.107	6.07E-03
Ush1g	1.576	2.003	2.145	0.928	0.681	0.440	2.106	1.53E-03
Alk	0.965	1.144	1.465	0.190	0.473	0.471	2.104	2.34E-03
Pnma1	1.586	1.975	2.345	0.424	0.912	0.910	2.101	1.91E-03
Cited1	5.636	5.416	6.284	3.703	3.466	4.368	2.090	1.01E-03
Arhgef38	0.539	0.421	0.891	0.061	0.276	0.178	2.083	4.46E-02
Stbd1	4.009	3.997	4.760	2.035	2.534	2.669	2.082	3.59E-05
Fam81a	3.224	3.301	4.292	1.743	1.893	2.108	2.071	3.24E-04
AABR07043453.1	4.541	4.502	4.953	2.982	2.142	2.943	2.060	7.21E-03
Sqrdl	3.452	3.426	3.859	1.085	2.012	2.329	2.059	3.12E-03

Shcbp1l	0.902	0.903	0.917	0.123	0.348	0.351	2.058	2.15E-03
Rn60_20_0054.5	3.451	3.806	4.359	1.791	2.183	2.362	2.052	2.89E-03
AABR07043389.1	3.872	3.277	4.667	2.145	1.288	2.832	2.041	1.05E-02
Btc	2.063	2.191	3.053	0.955	1.167	1.137	2.040	3.47E-04
LOC363337	5.451	5.759	6.348	4.099	3.694	3.905	2.037	1.12E-04
Prrt4	1.235	1.505	1.953	0.469	0.517	0.706	2.033	1.47E-03
Synpo2l	1.310	1.748	2.055	0.539	0.757	0.618	2.032	1.03E-03
Fam115c	1.543	1.538	1.952	0.514	0.536	0.802	2.029	1.89E-02
Ngfr	4.715	4.817	5.777	3.066	3.244	3.428	2.025	6.26E-04
Exnef	1.608	2.001	2.744	0.553	0.986	1.148	2.025	2.14E-03
Sntg1	1.068	1.457	1.387	0.391	0.457	0.475	2.025	3.65E-03
Ppp1r13l	2.086	2.759	3.249	0.937	1.515	1.307	2.023	3.00E-03
Plpp4	1.641	1.950	2.475	0.605	0.956	0.892	2.023	1.71E-03
Cdr2	5.589	5.916	6.424	3.484	3.788	4.688	2.022	1.56E-04
Agtr1a	1.701	1.964	2.762	0.597	0.933	1.183	2.011	9.63E-03
Eps8l1	0.644	1.059	1.297	0.293	0.272	0.395	2.009	2.13E-03
Lin7b	5.447	5.160	6.017	3.672	3.717	3.454	2.009	3.50E-04
Prom2	1.502	1.607	2.106	0.615	0.659	0.714	2.009	6.33E-05
Hsph1	8.078	7.998	8.404	5.940	5.893	6.612	2.007	1.73E-05
Robo3	0.789	1.408	1.121	0.162	0.634	0.299	1.998	9.63E-03
Htr2c	1.771	2.275	2.384	0.347	1.173	1.134	1.991	2.76E-03
Dll4	1.465	1.543	2.308	0.428	0.591	1.045	1.987	7.71E-04
Slit1	0.520	0.734	0.816	0.082	0.266	0.290	1.978	4.68E-02
Tap1	4.742	5.248	5.804	3.140	3.918	3.863	1.973	1.74E-05
Mmd2	3.202	3.300	4.010	1.720	2.096	1.830	1.966	1.10E-04
Slc26a9	0.237	0.549	0.570	0.027	0.183	0.180	1.956	2.81E-02
Scml4	1.450	1.709	2.295	0.502	0.826	0.897	1.949	3.42E-03
Arhgef15	1.504	1.775	2.040	0.640	0.949	0.453	1.947	1.12E-03
Pbx4	2.356	2.791	2.939	1.262	1.422	1.127	1.943	5.46E-04

Neto1	2.773	2.800	3.443	1.094	1.432	1.956	1.940	3.53E-04
Dusp13	1.210	1.415	0.929	0.303	0.623	0.134	1.936	1.23E-02
Creb3l1	4.709	5.195	5.820	2.660	3.578	3.918	1.932	3.36E-04
Pkd1l1	0.271	0.436	0.372	0.125	0.061	0.114	1.932	1.61E-02
Stx11	1.406	1.296	2.020	0.169	0.890	0.748	1.919	2.54E-02
Phactr2	3.964	4.176	4.431	2.052	2.480	2.865	1.916	1.09E-04
Slc16a14	5.009	5.179	5.841	3.141	3.597	3.795	1.916	1.02E-05
Mal	0.965	1.579	1.679	0.681	0.350	0.511	1.913	7.37E-03
Bend4	3.354	3.625	4.513	1.950	2.131	2.581	1.911	2.02E-02
Zap70	1.382	2.038	2.027	0.621	0.949	0.659	1.910	8.19E-03
Lmo2	5.328	5.430	6.093	3.468	3.706	4.205	1.906	2.59E-05
Cys1	2.258	2.400	2.878	1.031	1.173	1.317	1.902	2.18E-04
Alpk2	0.544	0.631	0.852	0.115	0.231	0.299	1.898	1.48E-03
Lck	3.508	3.765	4.353	1.655	2.359	2.622	1.898	1.36E-04
Nradd	3.212	3.957	4.267	1.801	2.346	2.431	1.896	1.58E-03
Gprc5a	1.495	1.773	2.294	0.333	1.085	0.860	1.893	2.15E-02
Nuak2	3.016	3.503	4.159	1.463	2.079	2.396	1.891	2.94E-03
Itpripl1	1.825	2.396	2.622	0.764	1.156	1.190	1.889	1.79E-03
Dnaaf3	1.660	2.171	2.032	0.256	1.240	0.884	1.889	2.79E-02
Whrn	2.427	2.613	3.301	1.116	1.362	1.701	1.886	3.69E-04
Lrrc38	2.582	2.688	2.725	1.121	1.526	1.196	1.883	1.09E-04
Rn60_20_0054.3	6.398	7.451	7.815	3.784	6.172	5.648	1.882	2.38E-02
C6	0.731	0.856	1.000	0.306	0.194	0.349	1.874	2.15E-03
Ar	1.109	0.979	1.573	0.478	0.317	0.534	1.874	3.98E-03
Accsl	0.465	0.547	0.708	0.149	0.233	0.151	1.873	6.55E-03
Lhx3	1.319	1.824	2.137	0.325	0.809	1.008	1.863	1.76E-02
Cilp2	0.778	1.199	1.640	0.311	0.362	0.662	1.861	5.75E-03
Fibin	0.588	1.008	1.198	0.152	0.445	0.382	1.858	4.78E-03
Mboat7l1	6.502	6.629	7.260	4.900	4.973	5.092	1.855	2.44E-06

Herc6	4.678	4.898	5.466	2.548	3.583	3.650	1.851	8.59E-04
Tmem252	0.393	0.780	0.927	0.113	0.198	0.368	1.850	2.35E-02
Cd164l2	3.699	3.735	4.390	2.542	1.886	2.434	1.848	7.45E-04
Pik3r5	0.847	1.153	1.503	0.395	0.446	0.459	1.844	2.15E-03
Chst8	3.183	3.226	3.587	1.212	1.641	2.414	1.841	4.50E-04
Scn10a	3.071	3.464	3.935	1.222	1.909	2.555	1.841	5.80E-03
Ociad2	6.900	6.663	6.943	5.023	5.221	4.872	1.829	1.79E-05
Rbm46	2.542	3.125	3.677	1.147	1.786	2.094	1.826	1.45E-02
Troap	1.565	1.818	2.264	0.576	0.965	0.915	1.824	8.45E-03
Cyb5rl	1.652	1.957	2.108	0.463	1.169	0.918	1.820	8.77E-03
Bst2	8.336	8.860	9.140	5.723	7.306	7.549	1.812	1.28E-02
Lrrc46	2.917	2.969	3.550	1.873	1.640	1.562	1.805	1.69E-04
PVR	3.708	4.353	4.849	2.026	2.997	2.997	1.803	2.85E-03
AABR07040840.1	4.122	5.068	4.496	2.853	3.244	2.703	1.800	1.03E-03
Hspa1b	6.078	6.161	6.988	3.862	4.419	5.440	1.799	8.85E-03
Stom	3.946	4.632	5.252	2.352	3.076	3.501	1.783	2.67E-03
Zmynd15	0.769	1.301	1.432	0.236	0.640	0.469	1.779	1.13E-02
Etv4	3.917	4.076	5.007	2.263	2.745	3.136	1.778	1.21E-03
Micb	2.048	2.497	2.828	0.889	1.540	1.191	1.773	3.99E-03
Fam65b	2.552	2.475	3.318	1.531	1.527	1.316	1.772	3.62E-04
Vgll2	1.081	1.202	1.465	0.451	0.408	0.585	1.767	8.81E-03
Dmrta1	0.145	0.256	0.268	0.031	0.112	0.063	1.760	4.80E-02
Pla2g4e	1.846	2.149	2.961	0.926	1.319	1.233	1.759	8.58E-04
Itgb7	0.487	0.691	1.093	0.134	0.267	0.402	1.759	3.05E-02
Cpq	3.834	3.845	4.445	1.958	2.880	2.562	1.757	1.15E-03
Cyp27b1	0.753	1.116	1.412	0.334	0.463	0.464	1.753	7.21E-03
Rel	2.408	2.852	3.467	1.582	1.576	1.597	1.751	1.15E-03
Rhpn1	4.966	5.175	5.804	3.509	4.153	3.398	1.743	4.53E-05
Cfap161	1.465	1.369	2.324	0.490	0.797	1.023	1.741	1.09E-02

RGD1562726	1.526	1.841	1.910	0.507	1.092	0.716	1.740	2.94E-02
Npr1	3.698	3.635	4.296	1.685	2.377	2.870	1.740	2.79E-04
Arhgap8	1.303	1.728	2.160	0.520	0.953	0.840	1.738	1.09E-02
Fgd5	1.001	1.206	1.510	0.338	0.758	0.368	1.736	8.79E-03
LOC108348293	3.514	4.322	4.159	1.673	2.760	2.874	1.734	8.30E-03
Cpeb1	2.264	2.520	3.298	1.006	1.602	1.697	1.733	2.13E-03
Mbnl3	0.891	1.147	0.870	0.192	0.551	0.365	1.733	3.58E-02
Slc18a3	0.258	0.445	0.567	0.058	0.102	0.246	1.729	1.77E-02
Adamts14	2.782	3.679	3.913	1.625	2.318	2.115	1.729	9.19E-03
Anks4b	2.178	1.981	2.134	0.757	1.180	1.051	1.727	4.64E-03
Capn3	0.748	0.810	0.903	0.087	0.542	0.250	1.724	2.23E-02
Dnah2	0.427	0.560	0.741	0.131	0.261	0.210	1.722	6.66E-03
Scn3a	4.681	4.897	4.691	2.773	3.449	3.309	1.721	3.69E-04
Lypd5	0.887	0.956	1.319	0.278	0.592	0.339	1.720	1.85E-02
Mns1	4.527	4.590	5.019	2.882	3.529	2.936	1.718	4.97E-04
B3galnt2	4.190	4.271	4.889	2.619	2.966	3.061	1.717	3.24E-04
LOC100910725	2.199	2.252	3.108	0.687	1.343	1.712	1.713	2.88E-02
Duox1	1.478	2.304	2.100	0.349	1.184	1.175	1.711	2.93E-02
Zdhhc11	0.413	0.482	0.622	0.081	0.188	0.233	1.710	4.18E-02
Tcaf2	1.705	1.772	1.670	0.460	0.894	0.939	1.705	3.47E-03
Sstr1	1.804	2.334	2.558	0.996	1.285	1.022	1.702	9.10E-05
Bfsp2	0.970	1.283	1.302	0.504	0.325	0.566	1.698	2.34E-03
Gck	1.718	1.996	2.425	0.978	0.952	1.041	1.698	2.94E-03
Ccdc159	3.582	3.832	4.099	2.002	2.579	2.592	1.696	1.05E-04
Mesp1	1.026	1.015	1.193	0.209	0.750	0.273	1.693	4.34E-02
Eps8l2	3.109	3.355	3.871	1.743	2.112	2.232	1.691	1.90E-05
Htr5b	0.232	0.484	0.645	0.044	0.243	0.179	1.687	1.83E-02
LOC501421	3.997	4.131	4.428	2.498	2.745	2.772	1.682	4.90E-03
Cav3	2.552	2.841	2.890	1.111	1.819	1.489	1.675	9.80E-04

Zfp575	1.925	2.245	3.336	1.222	1.404	1.460	1.673	1.19E-02
Scimp	0.694	0.989	0.945	0.282	0.428	0.285	1.670	1.37E-02
Cartpt	1.750	2.661	2.751	1.496	1.441	0.609	1.669	4.58E-02
Lap3	6.268	6.341	6.952	4.650	4.976	5.095	1.667	1.83E-06
Palm2	2.023	2.184	2.320	0.829	1.286	1.113	1.667	9.38E-04
Gal	3.766	4.590	4.029	1.921	3.058	2.848	1.664	2.23E-03
Eras	1.409	1.625	1.625	0.551	0.638	0.842	1.663	2.56E-03
Trip10	5.636	5.547	6.046	3.825	4.249	4.354	1.662	2.04E-06
Sftpc	0.714	1.421	1.224	0.564	0.296	0.471	1.662	3.05E-02
Lgals9	8.627	8.528	8.978	6.839	6.802	7.409	1.662	5.85E-05
Gimap8	0.971	1.373	1.780	0.375	0.737	0.701	1.654	2.03E-02
Cdt1	3.627	4.179	4.769	2.025	2.803	3.214	1.653	8.97E-04
Nr1h4	1.580	1.538	2.101	0.547	1.050	0.831	1.649	3.13E-03
Intu	3.558	3.894	4.163	2.185	2.355	2.625	1.648	8.74E-03
Dnajb1	7.525	7.602	8.004	6.029	5.895	6.284	1.646	8.66E-06
Mgam	0.403	0.693	0.525	0.152	0.237	0.203	1.642	1.80E-02
Calr3	2.677	2.822	2.730	1.278	1.659	1.563	1.641	7.17E-05
Klf1	1.819	1.773	1.976	0.683	0.782	1.140	1.636	1.24E-03
Rn60_17_0453.2	1.960	2.202	2.021	0.817	1.122	1.126	1.634	1.47E-03
Serpinh1	6.311	6.814	7.407	3.791	5.654	5.886	1.632	3.84E-02
Wnt5b	1.222	1.415	1.692	0.341	0.851	0.722	1.631	1.49E-02
Stc2	5.025	5.310	4.584	2.941	3.733	3.657	1.631	1.59E-03
Lingo3	4.237	4.124	4.726	2.361	2.834	3.342	1.627	2.59E-04
Bpifb1	3.298	4.694	4.993	2.657	2.625	3.461	1.626	5.24E-03
Rhoc	5.990	6.715	6.924	4.062	5.142	5.464	1.625	3.65E-03
Kcnb1	2.902	3.396	3.838	1.581	2.259	2.234	1.621	7.59E-03
Elmo3	1.690	1.992	2.531	0.790	1.074	1.256	1.621	2.32E-03
C2cd2	4.445	4.392	4.592	3.028	3.165	2.658	1.615	4.90E-05
Mrgprf	3.420	3.580	4.110	1.295	2.239	3.016	1.602	2.15E-02

Pygm	3.466	3.737	3.585	2.077	2.737	1.608	1.600	1.14E-02
Calhm2	3.768	4.106	4.157	2.330	2.516	2.857	1.599	1.09E-04
LOC498265	2.939	3.071	3.359	1.726	1.934	1.877	1.598	2.49E-04
Padi2	2.107	2.205	2.958	1.257	1.142	1.503	1.594	2.32E-03
Sgsh	3.720	3.896	4.429	2.152	2.698	2.910	1.593	2.71E-03
Gstk1	4.951	4.692	5.229	3.345	3.495	3.546	1.589	2.46E-05
AABR07042611.1	7.065	7.356	7.678	5.657	5.775	6.008	1.583	8.58E-04
Ltk	2.951	3.025	3.141	1.530	1.948	1.819	1.582	3.42E-05
Myo7b	0.188	0.312	0.456	0.128	0.119	0.088	1.577	1.90E-02
Sspo	0.152	0.175	0.260	0.046	0.072	0.087	1.567	3.10E-02
Rph3al	2.017	2.157	2.527	0.975	1.073	1.431	1.567	8.64E-04
Heatr5a	4.053	4.193	4.780	2.579	3.005	3.187	1.566	1.83E-03
Ldb3	0.747	0.885	0.874	0.340	0.471	0.270	1.565	2.89E-03
Mnd1	2.177	2.254	2.023	1.397	0.800	1.024	1.563	4.06E-02
RT1-CE4	6.090	6.865	7.291	4.399	5.601	5.567	1.561	3.08E-03
Prrt1	6.628	6.756	6.462	5.326	5.402	4.085	1.560	1.89E-02
Cep135	2.880	3.106	3.541	1.625	2.021	2.108	1.560	8.97E-04
Rasa4	5.269	5.624	5.047	3.924	4.043	3.437	1.559	1.15E-03
Ccdc189	1.762	2.410	2.731	1.224	1.459	1.011	1.558	7.03E-03
AABR07062033.1	3.694	3.348	4.231	2.241	2.705	2.406	1.554	2.00E-03
Ddn	1.551	1.800	2.626	0.620	1.097	1.366	1.553	2.15E-02
Zkscan2	3.658	3.662	3.886	2.312	2.622	2.182	1.552	8.11E-05
Plscr1	2.889	2.982	3.496	1.498	1.993	1.905	1.549	9.03E-04
Col5a1	3.951	4.836	4.504	2.194	3.273	3.416	1.545	2.18E-02
Pgf	2.110	2.475	2.100	0.964	1.423	1.151	1.543	4.30E-03
Plod2	4.378	4.430	4.883	2.763	3.226	3.476	1.534	1.65E-04
LOC108348065	7.107	7.151	7.390	5.744	5.806	5.493	1.530	1.51E-05
Slc4a11	2.027	2.388	2.907	1.081	1.440	1.543	1.528	2.17E-03
Mid1	0.838	1.269	1.757	0.375	0.623	0.842	1.511	2.61E-02

1700007B14Rik	1.639	2.044	1.970	0.513	1.131	1.223	1.511	2.75E-02
Mpdz	5.444	5.303	5.749	3.918	3.952	4.297	1.506	1.71E-04
Pus7	3.625	4.021	4.620	2.310	2.750	3.180	1.505	4.45E-03
Jak3	3.478	3.807	4.145	2.342	2.726	2.361	1.504	6.65E-05
Hap1	5.329	5.954	6.365	3.824	4.852	4.644	1.503	7.45E-04
Hn1l	4.724	5.110	5.861	3.255	3.878	4.332	1.503	1.59E-03
Slc27a2	1.105	0.758	1.607	0.565	0.533	0.508	1.500	2.56E-02
AABR07013288.4	2.689	3.219	3.352	2.006	2.096	1.438	1.498	9.81E-03
Tspan6	7.358	7.409	7.756	5.803	6.141	6.187	1.496	1.84E-07
Slc37a2	1.257	1.625	1.851	0.913	0.686	1.090	1.496	5.86E-04
Sipa1	1.936	2.443	2.525	0.616	1.545	1.523	1.495	6.50E-03
Setdb2	3.545	3.333	3.745	2.160	2.199	2.432	1.493	5.56E-05
Gramd2	0.759	0.870	0.940	0.356	0.486	0.226	1.491	2.12E-02
LOC100364500	3.971	4.744	5.336	2.547	3.592	3.767	1.489	3.16E-02
Tbx18	0.276	0.484	0.521	0.117	0.220	0.164	1.488	4.70E-02
Nfatc2	1.107	1.303	1.734	0.572	0.622	0.770	1.485	8.29E-03
Lca5	3.563	3.843	4.190	2.371	2.634	2.693	1.480	1.76E-04
Fignl1	1.354	1.845	2.012	0.812	0.885	0.990	1.466	8.91E-04
Crybg3	1.540	1.853	2.273	0.737	0.983	1.254	1.465	1.53E-02
Ulk3	3.721	3.595	4.088	2.306	2.478	2.748	1.457	4.56E-05
Gpr3	2.624	2.632	2.936	1.623	1.492	1.622	1.456	3.64E-04
Lcp2	0.609	1.027	0.941	0.206	0.318	0.591	1.451	1.89E-02
Zfp853	3.012	3.124	3.563	1.867	2.338	1.876	1.449	8.78E-04
Dclk3	0.904	1.582	1.600	0.383	0.705	0.903	1.447	2.67E-02
Msh5	0.764	1.157	1.446	0.426	0.623	0.491	1.445	6.07E-03
Tmem130	8.622	8.540	8.448	7.386	7.088	6.635	1.444	5.56E-03
Asl	5.784	5.897	6.073	4.086	4.781	4.617	1.442	5.08E-06
Hhat	2.222	2.350	3.173	1.193	1.395	1.925	1.442	3.86E-03
Slc12a5	5.417	5.210	5.811	4.106	4.330	3.765	1.438	5.39E-05

Hspa1l	1.084	1.421	1.268	0.408	0.693	0.695	1.438	2.90E-02
Lmo7	4.517	4.738	5.311	3.139	3.505	3.741	1.436	1.21E-03
Ift27	6.064	5.723	6.012	4.640	4.557	4.400	1.433	1.93E-04
Gstm1	6.226	6.756	7.142	4.775	5.531	5.580	1.432	4.48E-04
Dcbld1	4.325	4.290	5.156	3.252	3.243	3.402	1.421	5.14E-04
Echdc1	5.633	5.647	5.960	4.210	4.402	4.545	1.416	3.05E-06
Hck	2.499	2.374	2.757	1.360	1.224	1.832	1.415	4.94E-03
Scin	0.693	0.948	1.143	0.395	0.310	0.535	1.413	1.73E-02
Mis18a	1.791	1.974	2.808	0.897	1.562	1.286	1.411	4.15E-02
Mroh7	1.290	1.912	2.075	0.448	1.351	0.926	1.410	4.98E-02
Gprasp2	8.081	7.944	8.157	6.666	6.716	6.576	1.409	5.46E-05
Mtrf1l	5.391	5.496	5.920	4.103	4.346	4.361	1.408	4.05E-05
Carns1	1.297	1.447	2.148	0.634	0.936	0.989	1.408	1.14E-02
Dnaaf1	0.272	0.650	0.780	0.240	0.232	0.242	1.406	2.41E-02
Plin2	6.240	6.460	6.768	4.353	5.422	5.451	1.406	1.55E-03
Mtmr11	3.324	3.805	3.929	1.993	2.722	2.626	1.405	2.09E-03
Syt5	7.447	7.536	7.924	6.391	6.037	6.226	1.404	2.00E-03
Ethe1	5.948	5.954	6.514	4.690	4.897	4.754	1.399	1.14E-04
Anxa4	4.615	4.950	4.760	3.004	3.822	3.586	1.396	1.74E-04
Zcchc12	6.405	6.272	6.941	5.126	5.183	5.278	1.386	6.31E-05
Klhl10	0.862	1.054	1.563	0.386	0.849	0.446	1.380	3.11E-02
Lxn	5.288	5.792	5.633	4.048	4.591	4.078	1.378	3.64E-04
LOC102551435	1.976	2.137	2.640	1.228	1.130	1.497	1.378	2.69E-03
LOC619574	5.230	5.451	6.022	4.001	4.276	4.484	1.378	3.45E-05
Nphs2	1.074	1.766	1.565	0.575	0.925	0.768	1.374	1.64E-02
Mtus2	3.439	4.183	3.939	2.447	3.231	3.238	1.369	1.09E-04
Lacc1	2.745	3.075	3.403	1.886	1.986	2.038	1.367	9.04E-03
Prim2	5.041	5.008	5.130	3.910	3.704	3.650	1.362	3.24E-04
Des	3.671	4.527	4.633	1.965	3.403	3.513	1.354	2.65E-02

Arhgef28	2.108	2.553	2.408	1.115	1.617	1.438	1.350	1.70E-03
Ramp2	3.268	3.562	4.680	2.460	2.470	3.151	1.348	2.15E-02
Il17re	2.441	2.816	2.696	1.429	1.809	1.609	1.347	2.15E-03
Doc2a	4.892	4.934	6.035	3.560	3.862	4.633	1.347	4.15E-03
Vstm2a	3.859	4.027	4.714	2.927	3.205	2.858	1.340	2.33E-03
Lman2l	7.335	7.240	7.402	6.094	6.073	5.725	1.338	1.05E-04
Sdk2	2.019	2.455	2.703	1.170	1.627	1.554	1.337	3.25E-02
Zfp503	2.555	2.629	3.029	1.552	1.717	1.827	1.334	4.85E-06
Myrip	2.110	2.523	3.089	1.144	1.705	1.888	1.332	1.16E-02
Ttc39a	2.054	2.119	2.518	1.004	1.263	1.628	1.329	8.64E-04
Cenpn	4.195	4.358	5.043	3.130	3.116	3.684	1.327	3.56E-04
Vwa3b	1.585	2.103	1.910	0.598	1.185	1.081	1.327	2.37E-02
Dtnb	5.243	5.441	5.920	4.246	4.226	4.335	1.326	6.84E-05
Kank2	2.400	2.761	2.980	0.899	1.862	2.091	1.324	2.85E-02
Rec114	1.636	1.125	1.721	0.728	0.616	1.004	1.324	2.51E-02
Timp3	3.842	3.965	4.429	2.727	2.770	3.141	1.323	8.00E-05
Nfkbid	2.323	2.493	2.938	1.461	1.665	1.638	1.313	9.59E-04
Olr287	1.770	1.602	1.648	0.763	1.293	0.611	1.311	1.43E-02
Xkr8	2.461	2.788	3.192	1.445	1.890	2.004	1.306	6.87E-04
Bbx	5.159	5.063	5.840	3.980	4.087	4.352	1.302	3.43E-02
Col2a1	1.157	1.293	1.475	0.594	0.706	0.716	1.296	1.94E-04
Stk3	3.762	4.073	4.605	2.761	3.253	2.963	1.294	3.04E-04
P4ha1	5.033	5.194	5.622	3.640	4.142	4.352	1.293	8.97E-04
Golm1	4.352	4.754	5.121	3.432	3.447	3.757	1.293	2.78E-03
Btg3	5.110	5.529	5.970	4.009	4.397	4.544	1.293	6.32E-04
Cib2	5.039	4.931	5.071	4.040	3.857	3.267	1.292	5.97E-03
Vegfb	5.149	5.230	5.487	3.796	4.128	4.210	1.292	5.19E-06
Rgn	3.485	3.591	3.516	1.986	2.925	2.239	1.284	9.16E-03
RT1-CE5	6.374	7.268	7.662	4.637	6.282	6.362	1.283	4.64E-02

Cage1	1.500	1.204	1.757	0.689	1.036	0.702	1.282	1.22E-02
Plekha7	3.374	3.512	3.921	2.320	2.542	2.589	1.274	2.64E-03
Hsd17b1	1.701	2.269	2.674	1.128	1.349	1.561	1.268	6.95E-03
Pacsin2	6.948	6.920	7.391	5.681	5.698	6.085	1.264	3.27E-04
Lrrc17	3.771	4.425	4.107	2.301	2.967	3.491	1.263	2.41E-03
Dct	2.276	2.257	2.966	1.479	1.419	1.784	1.262	8.90E-03
Akap2	3.628	3.936	4.370	2.560	3.094	3.126	1.261	2.78E-02
Sertad3	2.975	3.158	3.766	1.835	2.435	2.447	1.257	2.50E-03
Tacr3	1.663	1.630	2.073	1.339	0.948	0.628	1.256	5.00E-02
Plekhs1	1.134	1.576	1.735	0.583	0.813	1.034	1.256	1.89E-02
Il1r2	2.075	2.187	2.035	0.882	1.338	1.497	1.255	1.70E-02
Anxa11	5.183	5.489	5.975	3.622	4.656	4.648	1.252	1.07E-03
Psmb9	5.335	5.363	6.083	3.732	4.667	4.691	1.252	2.15E-03
Fam210b	3.920	3.513	4.341	2.646	2.796	3.027	1.251	8.98E-04
Npepl1	4.928	5.159	5.434	3.580	4.075	4.243	1.250	2.70E-05
Apba3	3.486	3.910	4.356	2.418	2.920	3.069	1.244	4.60E-04
Nwd2	0.943	1.330	1.260	0.481	0.854	0.526	1.236	3.11E-02
Serpinb6	4.175	4.698	4.997	2.944	3.787	3.680	1.231	7.59E-03
Fsbp	2.045	1.698	2.387	0.853	1.493	1.332	1.230	4.54E-02
Uba7	3.596	3.644	3.961	2.703	2.566	2.603	1.229	4.53E-05
Lama3	4.826	5.621	5.126	3.402	4.376	4.343	1.221	5.55E-03
Lims2	0.980	1.020	1.274	0.490	0.333	0.733	1.214	1.36E-02
Hirip3	6.141	5.961	6.718	4.870	5.038	5.423	1.212	3.32E-04
LOC654482	2.625	3.224	3.262	1.642	2.238	2.264	1.211	2.48E-02
Psrc1	2.216	2.783	3.269	1.381	2.005	2.075	1.209	4.37E-03
Bmp2	1.078	1.826	1.374	0.593	0.934	0.907	1.208	1.33E-02
Rbpjl	0.538	1.126	0.860	0.383	0.447	0.460	1.207	2.17E-02
Mthfr	3.694	4.077	4.200	2.428	3.116	3.106	1.206	2.32E-03
Tmem35	7.471	7.267	7.526	6.327	6.390	5.854	1.205	9.33E-03

Gusb	3.859	3.967	4.406	2.699	3.052	3.188	1.205	4.08E-04
Epha8	3.407	3.709	4.368	2.210	3.026	3.018	1.204	3.33E-03
Rcan3	6.043	6.351	6.802	5.014	5.361	5.326	1.202	3.73E-04
Pgm3	4.324	4.484	5.114	3.115	3.479	3.962	1.202	1.74E-03
Ahsa2	6.889	6.748	6.947	5.617	5.659	5.796	1.201	4.42E-06
Erp29	7.533	7.580	8.096	6.357	6.497	6.855	1.199	3.23E-05
Rnf213	4.178	4.195	4.708	2.756	3.397	3.571	1.199	1.18E-02
Prkab2	4.035	4.063	4.636	2.960	3.170	3.423	1.199	3.60E-04
Tmem44	3.267	3.524	4.077	2.429	2.697	2.643	1.196	8.37E-04
Pgm2	5.274	5.383	5.885	4.277	4.434	4.445	1.191	1.74E-05
Fam131c	4.630	4.116	5.154	3.669	3.387	3.544	1.190	1.34E-02
Slc26a8	2.712	2.947	3.422	1.709	2.249	2.248	1.188	1.59E-03
Aard	3.861	4.316	4.436	2.743	3.464	3.149	1.183	1.20E-03
Tifa	3.657	4.051	4.485	2.579	3.279	3.272	1.182	5.11E-03
Unc5d	3.759	3.852	4.135	2.868	2.935	2.715	1.181	3.24E-03
Hsp90ab1	11.636	11.605	11.790	10.547	10.254	10.623	1.178	3.82E-04
Cadm1	5.812	5.861	6.515	4.540	5.053	5.171	1.167	1.15E-03
Aldh1l2	4.064	4.772	5.050	2.717	4.114	3.659	1.166	2.18E-02
Bahd1	5.036	5.186	5.541	4.035	4.204	4.155	1.166	8.66E-06
Tmem98	2.891	3.424	3.721	1.980	2.529	2.571	1.161	2.72E-02
Gfra1	1.327	1.735	2.105	0.745	1.064	1.275	1.160	2.63E-02
Klhl40	2.558	2.568	3.248	1.513	1.649	2.374	1.159	3.60E-02
Kcnd1	3.633	3.846	3.915	2.712	3.059	2.407	1.157	4.25E-03
Zfp772	2.140	2.244	2.462	1.344	1.741	1.215	1.156	3.21E-02
Dnaaf2	3.873	4.065	4.554	2.770	3.196	3.368	1.156	9.30E-05
Kcne2	2.633	2.741	2.943	1.302	1.908	2.279	1.153	1.64E-02
AABR07065031.1	0.789	1.067	1.094	0.302	0.510	0.755	1.153	2.75E-02
Gprasp1	8.357	8.242	8.360	7.202	7.188	7.122	1.146	6.33E-05
RGD1304624	4.068	4.487	4.814	3.204	3.349	3.662	1.140	3.28E-04

Grid2ip	2.556	2.920	3.100	1.340	2.369	2.009	1.134	3.31E-02
Relt	2.073	2.180	2.785	1.500	1.500	1.778	1.132	7.77E-04
Ndufaf2	4.066	3.541	4.401	2.829	3.190	3.036	1.131	5.58E-03
Crb3	1.082	1.426	1.234	0.741	0.865	0.573	1.128	8.14E-03
Cyb561	4.615	4.451	4.816	3.487	3.560	3.512	1.124	5.14E-04
Epha6	3.742	3.152	3.381	2.620	2.201	2.560	1.124	7.38E-03
Plek2	0.811	0.848	0.910	0.369	0.437	0.560	1.123	9.63E-03
Pnma3	3.999	4.206	4.526	3.079	3.345	3.169	1.123	1.09E-04
Agpat5	5.432	5.375	5.869	4.234	4.478	4.747	1.122	2.85E-05
Pnpla7	4.230	4.749	4.422	3.015	3.757	3.466	1.120	8.27E-03
Cacna2d2	6.102	5.885	6.510	5.092	5.205	4.866	1.117	2.56E-04
Tmem14a	6.032	6.002	6.207	4.756	5.191	5.008	1.117	4.73E-04
Matk	3.890	4.011	4.006	2.921	3.070	2.838	1.115	2.85E-03
Slc44a3	2.063	1.968	1.768	1.176	1.387	1.044	1.115	9.77E-03
Krt80	0.499	0.844	1.128	0.327	0.471	0.526	1.114	3.39E-02
Snx3	8.927	9.049	9.195	7.955	7.902	8.007	1.113	1.71E-04
Tlcd1	1.027	1.188	1.583	0.624	0.857	0.700	1.112	7.32E-03
Msl3l2	3.851	4.158	4.594	2.891	3.130	3.517	1.112	6.97E-04
AABR07034750.1	0.991	0.924	1.083	0.565	0.461	0.607	1.111	2.11E-02
Sec23b	4.442	4.777	5.083	3.277	3.882	3.996	1.109	1.14E-03
Ulk4	1.575	1.551	1.629	0.630	1.097	1.099	1.109	3.40E-03
Cacng8	3.572	3.505	3.970	2.702	2.628	2.747	1.106	1.35E-03
Pygl	1.593	2.115	2.051	0.864	1.118	1.582	1.106	3.45E-02
Hspa4	8.340	8.122	8.573	7.177	7.176	7.418	1.105	1.90E-05
Mx2	5.487	5.617	6.437	4.085	4.827	5.361	1.104	1.89E-02
Cdk18	1.511	1.883	2.251	1.093	0.986	1.420	1.102	9.37E-03
Ccdc184	3.774	3.856	4.742	3.221	2.973	3.206	1.101	1.14E-02
Wdr66	5.718	5.609	5.857	4.625	4.791	4.547	1.100	1.74E-03
Dhcr7	5.053	4.983	4.968	3.973	3.814	4.051	1.100	7.39E-03

Pnck	5.298	5.463	5.424	4.207	4.546	4.196	1.099	4.41E-04
Tecta	0.540	0.436	0.624	0.176	0.290	0.358	1.098	3.37E-02
Borcs6	4.266	4.522	4.935	3.508	3.416	3.699	1.097	2.26E-04
Fam151b	3.011	2.842	3.409	2.248	2.009	2.273	1.093	8.62E-03
Il17rc	1.782	2.113	2.208	0.927	1.411	1.505	1.092	2.94E-02
Chrm4	1.561	1.889	2.731	1.237	1.219	1.589	1.091	2.14E-02
Tigar	5.121	5.089	5.711	4.101	4.236	4.483	1.089	1.59E-04
Rbm20	0.887	1.038	0.797	0.384	0.675	0.441	1.089	1.55E-02
Parp12	3.606	4.100	4.685	2.463	3.256	3.655	1.089	1.83E-02
Trim65	4.378	4.474	4.898	3.191	3.864	3.602	1.087	2.95E-04
Klf8	2.788	2.798	3.425	1.733	2.311	2.327	1.085	1.44E-02
LOC680254	2.553	2.963	3.517	1.917	2.208	2.284	1.083	2.22E-02
Hpcal4	3.786	3.743	4.540	2.783	3.132	3.261	1.083	1.43E-03
Ifih1	2.702	3.142	3.714	1.594	2.459	2.745	1.083	3.99E-02
Aff1	2.993	3.358	3.932	2.365	2.658	2.549	1.081	3.96E-02
Chrnb1	2.667	2.867	2.867	1.656	2.048	2.091	1.081	4.26E-04
Ampd3	2.547	3.154	3.783	1.659	2.780	2.338	1.076	3.92E-02
Samd15	1.905	2.304	2.609	1.184	1.528	1.818	1.074	3.83E-02
Plcd3	2.531	2.671	3.402	1.595	2.132	2.298	1.074	8.14E-03
Mthfd2	4.018	4.291	4.933	2.844	3.329	4.026	1.070	5.29E-03
Tspan33	3.758	4.007	4.081	2.590	3.262	3.040	1.067	1.50E-04
Pcp4	5.605	6.400	5.587	4.533	5.072	5.001	1.065	1.08E-02
Dynll1	9.908	9.788	10.053	8.914	8.709	8.930	1.063	6.71E-03
Gng7	1.220	1.209	1.558	0.571	0.825	0.952	1.062	3.16E-02
Rai2	2.823	3.175	3.677	1.991	2.468	2.547	1.060	2.95E-03
Ptpn13	3.444	3.695	4.293	2.653	2.927	3.093	1.058	8.24E-03
Derl3	3.161	3.179	3.287	2.008	2.330	2.551	1.058	8.45E-03
LOC103694875	3.161	3.179	3.287	2.008	2.330	2.551	1.058	8.45E-03
Ckmt2	4.213	4.164	4.300	3.133	3.136	3.466	1.057	4.96E-02

Fam167a	3.521	4.056	4.485	2.759	3.359	3.136	1.052	1.83E-03
Hspa2	4.611	4.864	5.409	3.482	4.140	4.275	1.049	5.30E-03
Rn60_12_0305.2	2.014	2.616	2.798	1.737	1.689	1.637	1.047	1.64E-02
Hadh	3.228	3.672	4.122	2.254	3.052	2.945	1.045	2.64E-02
Edrf1	5.264	5.432	5.722	4.303	4.559	4.564	1.041	1.10E-05
Arl15	4.856	4.850	5.566	3.873	4.056	4.431	1.038	1.43E-02
Phyhd1	4.831	4.773	4.972	3.626	4.004	3.971	1.037	2.15E-04
Gtf2f2	4.859	5.006	5.485	4.091	4.027	4.307	1.036	3.24E-04
Coro6	4.626	4.798	5.218	3.642	3.955	4.164	1.036	1.59E-03
Camkk1	3.907	3.960	4.608	2.946	3.305	3.396	1.036	3.69E-04
Nfatc2ip	3.157	3.199	3.953	2.327	2.484	2.843	1.032	4.37E-03
Pomt1	4.486	4.481	4.840	3.520	3.673	3.698	1.028	9.47E-05
Ocln	2.713	2.552	2.935	2.034	1.891	1.819	1.021	7.37E-03
Plekhh1	2.181	2.123	2.937	1.741	1.628	1.620	1.019	4.29E-03
Slc35a3	5.528	5.377	5.981	4.419	4.745	4.835	1.018	4.07E-03
Opa3	5.272	5.441	5.715	4.496	4.398	4.552	1.016	6.65E-05
Atad2	3.938	3.910	4.413	2.869	3.346	3.308	1.015	6.36E-03
Aldh1b1	1.258	1.239	1.349	0.561	0.958	0.793	1.013	3.60E-02
Prpf40b	5.199	5.383	5.571	4.264	4.480	4.476	1.008	2.37E-04
Ghr	3.178	4.040	3.957	2.603	2.865	3.159	1.008	2.48E-02
Gpx8	5.598	5.805	6.540	4.192	5.302	5.460	1.006	4.68E-02
Acer2	1.479	1.457	2.306	1.041	1.049	1.345	1.004	1.84E-02
Dnaja4	5.712	5.445	5.859	4.668	4.604	4.829	1.002	1.71E-04
Mok	3.053	3.252	3.541	2.110	2.089	2.490	0.998	2.48E-02
Sap30	5.870	6.059	6.733	5.208	5.289	5.347	0.998	1.85E-02
Hyal2	4.719	4.844	4.664	3.827	3.828	3.709	0.994	1.53E-03
Abhd2	5.736	5.767	6.423	4.696	5.046	5.296	0.993	6.51E-03
Slc35c1	5.087	5.331	5.762	4.238	4.383	4.665	0.988	3.64E-04
Acss2	6.574	6.717	6.587	5.649	5.764	5.513	0.987	1.16E-03

Nmrk1	3.333	3.043	3.225	2.448	2.452	2.195	0.987	9.61E-03
Muc13	1.778	2.116	2.209	1.236	1.420	1.447	0.983	1.18E-02
Tapbpl	4.698	5.026	5.378	3.617	4.354	4.289	0.980	2.34E-03
Bcap31	7.744	7.792	7.873	6.743	6.842	6.930	0.977	8.01E-05
Zfp365	4.287	4.308	4.539	3.539	3.556	3.275	0.973	8.97E-04
Znf354b	2.311	2.244	2.791	1.786	1.557	1.837	0.973	1.26E-02
Ccdc14	2.242	2.271	2.531	1.447	1.853	1.624	0.952	5.74E-04
Dock10	3.592	3.597	4.046	2.574	3.052	3.201	0.951	8.86E-03
Adra2c	4.760	4.848	5.140	3.451	3.955	4.498	0.945	6.46E-03
Baiap2l2	1.370	1.673	1.829	1.174	1.039	0.914	0.942	4.15E-02
Ogfod1	3.808	4.058	4.711	3.100	3.400	3.558	0.941	5.34E-03
Akip1	3.813	4.124	4.452	3.010	3.494	3.294	0.941	1.00E-03
Polk	2.516	2.297	2.901	1.781	1.858	1.793	0.941	3.93E-03
AABR07043564.1	2.999	3.385	3.741	2.316	2.475	2.906	0.937	1.28E-02
B3glct	3.197	3.545	4.170	2.387	2.868	3.194	0.937	4.17E-02
Gnpda1	4.508	4.858	5.276	3.745	4.192	4.070	0.935	3.24E-03
Nsun5	5.312	5.249	5.497	4.396	4.402	4.536	0.932	1.59E-04
Mycbpap	2.012	2.433	2.312	1.470	1.814	1.358	0.932	5.93E-03
Trim7	1.719	1.928	1.899	0.906	1.394	1.392	0.931	4.75E-02
Twf2	4.619	4.709	5.239	3.640	3.803	4.417	0.927	7.37E-03
Slc39a11	3.153	3.272	3.714	2.227	2.475	2.975	0.923	3.86E-03
Pusl1	4.525	4.946	5.104	3.706	4.098	4.138	0.922	2.97E-04
Cct8	8.786	8.570	8.870	7.821	7.726	7.953	0.922	1.09E-03
Rom1	2.876	3.482	3.575	1.886	2.819	2.774	0.914	2.52E-02
Fkbp4	7.687	7.597	7.782	6.805	6.610	6.873	0.913	1.56E-03
Nnat	9.047	8.967	9.136	8.300	8.082	7.882	0.913	4.98E-02
Parp9	4.529	4.743	5.085	3.500	4.150	4.119	0.911	2.00E-03
Tmem159	3.556	3.199	3.593	2.848	2.713	2.287	0.910	3.80E-02
Ubxn2a	5.110	5.380	5.835	4.313	4.715	4.742	0.907	7.48E-03

Kcnf1	4.562	4.334	4.622	3.520	3.662	3.773	0.906	5.39E-05
Zfp593	3.904	3.954	4.383	3.185	3.229	3.326	0.902	1.14E-03
Stk36	2.735	3.036	3.453	2.008	2.555	2.397	0.899	1.62E-02
Sesn2	4.866	5.303	5.583	4.036	4.514	4.615	0.895	2.42E-04
Kcnh8	1.759	1.856	2.357	1.050	1.286	1.795	0.889	2.36E-02
Polr2h	5.406	5.448	5.972	4.708	4.692	4.843	0.888	2.81E-03
St14	3.132	3.272	3.709	2.473	2.661	2.667	0.885	4.11E-03
Ces5a	1.406	1.935	2.020	1.202	1.256	1.218	0.878	1.87E-02
LOC100911516	9.592	9.488	9.246	8.472	8.749	8.490	0.877	7.40E-03
Osbpl3	3.673	3.696	3.875	3.038	2.905	2.921	0.872	1.61E-02
Fgd1	5.146	5.189	5.792	4.512	4.507	4.613	0.872	5.50E-04
Dock9	4.779	4.878	5.305	4.015	4.317	4.123	0.870	6.23E-04
Prkcdbp	5.864	5.909	6.399	4.970	4.977	5.623	0.870	6.22E-03
Mrfap1	9.824	9.742	9.936	8.802	9.010	9.012	0.868	2.97E-04
Rab39a	5.105	5.094	5.390	4.225	4.418	4.470	0.868	8.00E-05
Hmgn1	7.443	7.413	7.923	6.342	6.561	7.237	0.867	6.20E-03
Gaa	6.280	6.705	6.814	5.313	6.009	5.859	0.866	1.15E-03
Actr5	3.239	3.384	3.791	2.468	2.787	2.889	0.865	3.97E-03
Cenpk	2.505	2.721	3.244	1.987	1.969	2.455	0.864	3.47E-02
Zbed5	5.108	4.949	4.930	4.208	4.076	4.253	0.858	1.23E-03
Adcy3	4.199	4.221	4.912	3.384	3.696	3.883	0.857	2.41E-03
Klhl25	3.504	3.576	4.048	2.737	3.057	3.017	0.857	1.86E-02
Ltbp3	5.020	5.028	5.482	4.097	4.321	4.619	0.855	4.51E-04
Ccdc134	3.227	3.580	3.895	2.404	3.070	2.945	0.855	5.22E-03
Cenpl	3.587	3.781	4.052	3.058	2.979	2.998	0.853	7.23E-03
Ppidl1	4.573	4.423	4.896	3.759	3.656	4.077	0.852	2.30E-02
Cbfb	5.697	5.854	6.481	4.727	5.426	5.479	0.851	1.38E-02
AABR07043445.1	4.195	4.564	4.684	3.571	3.719	3.758	0.850	2.48E-02
Galnt13	5.718	5.353	5.241	4.854	4.606	4.406	0.849	4.05E-02

Heg1	4.330	4.553	5.107	3.537	3.995	4.083	0.848	3.00E-02
Khnyn	5.124	4.991	5.306	4.178	4.589	4.143	0.847	4.15E-03
Dbf4	2.403	2.367	2.469	1.561	1.865	1.904	0.845	4.94E-03
Hltf	6.801	6.673	6.749	5.877	5.941	5.936	0.845	4.19E-04
Cnbd2	1.848	1.728	2.292	1.368	1.341	1.438	0.844	7.74E-03
Cercam	2.191	2.508	2.619	1.493	1.945	1.913	0.843	2.50E-02
Dnajb3	2.938	2.918	3.654	2.111	2.493	2.783	0.841	2.98E-02
Cct5	8.972	8.872	9.083	8.079	8.036	8.303	0.833	1.33E-03
Ppme1	7.588	7.583	7.844	6.841	6.794	6.883	0.829	6.25E-04
LOC362863	0.968	1.173	0.944	0.547	0.970	0.770	0.828	2.04E-02
Mast3	3.990	4.175	4.840	3.077	3.791	3.816	0.826	1.14E-02
Ntan1	7.972	7.966	7.987	7.502	7.166	7.026	0.824	6.46E-03
Haus8	4.600	4.597	5.052	3.715	3.918	4.273	0.822	1.25E-03
Xylb	4.340	4.639	5.142	3.422	3.968	4.356	0.821	3.47E-02
Sdhaf4	7.094	7.234	7.371	6.385	6.201	6.653	0.820	2.69E-02
AABR07058884.1	0.695	0.775	0.831	0.418	0.503	0.522	0.820	3.16E-02
Mphosph9	3.703	3.818	4.117	2.895	3.171	3.408	0.819	6.47E-03
Iqcb1	5.598	5.712	5.972	4.936	4.995	4.992	0.819	8.18E-04
Cept1	5.813	5.740	6.102	4.883	5.240	5.178	0.816	1.12E-04
Ppm1m	2.137	2.283	2.605	1.323	1.698	2.119	0.814	4.58E-02
Ncln	5.922	6.130	6.263	5.138	5.291	5.463	0.812	7.09E-05
Nfatc3	4.256	4.353	4.823	3.629	3.715	3.909	0.812	2.50E-03
Sync	5.021	4.919	5.209	4.023	4.308	4.496	0.811	4.12E-02
Wfs1	2.988	3.247	3.615	2.523	2.642	2.566	0.811	1.93E-03
Tmem206	5.031	5.040	5.563	4.115	4.457	4.723	0.809	2.87E-03
LOC103689949	3.193	3.432	3.868	2.728	2.592	3.018	0.805	1.48E-02
Nucb1	7.658	7.752	7.853	6.881	7.067	6.899	0.805	2.20E-04
Cct6a	8.604	8.499	8.737	7.733	7.752	7.980	0.800	5.41E-04
Rhof	3.888	3.681	4.313	3.125	3.121	3.457	0.799	2.14E-02

Casp7	4.478	4.992	5.301	3.815	4.277	4.435	0.799	1.31E-02
Wdr54	5.478	5.617	5.676	4.709	4.785	4.913	0.798	3.66E-03
Tmem268	2.235	2.707	2.845	1.741	2.037	2.075	0.798	2.83E-03
Arl6ip4	5.543	5.789	6.081	5.050	4.958	5.040	0.796	5.02E-03
Inpp5a	5.321	5.381	5.821	4.632	4.638	4.936	0.795	4.87E-03
Hacd3	7.280	7.274	7.607	6.472	6.592	6.754	0.795	2.79E-04
Gorasp2	6.526	6.656	7.204	5.794	5.973	6.291	0.794	1.05E-03
Orai3	3.042	3.400	3.526	2.299	2.853	2.719	0.794	9.60E-03
Usp18	4.682	4.761	5.461	3.955	4.290	4.462	0.794	2.42E-02
Ap5b1	2.858	3.315	3.620	2.382	2.612	2.760	0.790	6.30E-03
Rasgef1a	3.955	4.020	4.634	3.250	3.475	3.730	0.790	6.74E-03
Emc8	5.883	6.116	6.348	5.182	5.287	5.539	0.789	1.41E-04
Atg3	7.485	7.422	7.750	6.617	6.917	6.892	0.789	1.50E-04
Prkar1b	8.263	8.208	8.085	7.397	7.616	7.121	0.786	2.28E-02
Ctr9	6.413	6.451	6.706	5.665	5.801	5.803	0.785	6.90E-05
Popdc2	1.127	1.210	1.628	0.740	1.021	0.949	0.785	4.33E-02
Smoc1	2.722	2.997	3.518	2.148	2.623	2.508	0.783	1.50E-02
RGD1308106	5.456	5.619	6.066	4.944	4.952	5.106	0.782	6.26E-04
Cers5	6.439	6.577	6.932	5.766	5.837	6.059	0.782	4.34E-04
Socs1	3.080	2.977	3.308	2.086	2.320	2.864	0.781	2.83E-02
Sin3b	7.381	7.665	7.901	6.810	6.910	6.890	0.772	1.13E-04
Naa16	4.669	4.798	4.841	3.927	4.205	4.022	0.771	1.51E-04
Clcn1	2.174	2.493	2.406	1.644	2.030	1.593	0.771	3.37E-02
Nat6	4.567	4.940	4.763	3.894	4.159	3.993	0.770	4.91E-04
Hs2st1	5.534	5.726	6.073	4.780	5.188	5.146	0.770	1.93E-04
Me2	5.075	5.201	5.348	4.285	4.508	4.557	0.768	1.14E-04
Wdr46	4.603	4.929	5.313	3.958	4.301	4.404	0.768	2.09E-03
LOC100910854	3.495	3.703	4.171	2.840	3.007	3.443	0.768	4.61E-03
Kcnc3	5.032	5.140	5.427	4.383	4.544	4.389	0.766	1.93E-04

Robo1	2.718	2.681	3.427	2.112	2.387	2.484	0.765	4.28E-02
Herc4	4.344	4.552	5.030	3.772	3.987	4.057	0.765	2.25E-03
LOC102548695	3.971	4.046	4.400	3.402	3.522	3.375	0.765	1.35E-03
Tmem51	3.603	3.629	3.915	2.832	3.148	3.116	0.765	2.53E-02
Tut1	4.203	4.316	4.591	3.520	3.625	3.800	0.764	1.07E-03
Tcp1	8.533	8.393	8.700	7.804	7.639	7.920	0.762	5.02E-03
Parm1	3.856	4.150	4.183	3.293	3.417	3.366	0.762	5.96E-03
Armcx6	4.222	4.659	4.594	3.800	3.957	3.483	0.761	3.25E-02
Apip	3.866	3.993	4.472	3.105	3.619	3.544	0.761	6.07E-03
Itpk1	5.720	5.663	5.905	4.703	4.901	5.391	0.760	7.80E-04
Prox1	3.939	4.087	4.604	3.326	3.465	3.760	0.758	8.71E-03
Grm1	3.792	3.804	4.283	3.172	3.381	3.252	0.758	8.64E-04
Cand2	2.507	2.856	3.002	1.983	2.382	2.166	0.757	1.25E-02
Sirt7	3.678	3.931	4.555	3.152	3.359	3.623	0.753	1.27E-02
Stat1	6.333	6.229	6.330	5.453	5.732	5.478	0.752	1.22E-02
Pir	5.200	5.361	5.505	4.409	4.733	4.771	0.749	3.91E-03
Dalrd3	4.197	4.389	4.527	3.531	3.564	3.878	0.748	1.85E-03
Plcd1	3.937	4.452	4.600	3.317	3.764	3.820	0.744	9.59E-03
Lsm2	5.423	5.656	5.774	4.682	5.096	4.854	0.743	1.27E-03
Zfp516	3.180	3.425	3.867	2.678	2.987	2.853	0.742	3.76E-02
Celf6	3.997	4.255	4.692	3.555	3.745	3.591	0.741	6.30E-03
Elob	9.900	9.985	10.150	9.277	9.286	9.252	0.741	3.47E-02
Dnajb2	6.255	6.237	6.443	5.580	5.533	5.585	0.740	5.30E-03
Cct2	8.464	8.360	8.522	7.717	7.651	7.777	0.740	4.30E-03
Ift43	4.653	4.614	5.122	3.993	4.145	4.189	0.740	1.41E-02
Spred3	4.593	4.758	4.873	3.653	3.786	4.239	0.739	2.02E-02
Prkx	5.064	5.009	5.262	4.125	4.431	4.315	0.738	3.42E-05
Maff	4.056	4.156	4.806	3.411	3.700	3.870	0.738	1.12E-02
Stx2	4.379	4.777	4.930	3.624	4.297	4.160	0.736	1.86E-02

Ccne2	4.763	4.788	5.219	4.072	4.441	4.227	0.735	1.90E-03
Slc31a1	5.614	5.843	6.277	5.037	5.293	5.291	0.734	7.02E-04
Cnpy4	4.515	4.674	5.122	3.786	4.035	4.389	0.734	6.49E-03
Dhx29	4.353	4.291	4.742	3.670	3.655	4.009	0.734	1.77E-03
Tmx1	8.371	8.216	8.555	7.615	7.640	7.749	0.733	8.47E-04
Celf5	4.252	4.413	4.924	3.650	3.849	4.044	0.732	2.50E-03
Fam118a	5.284	5.199	5.591	4.435	4.666	4.850	0.732	8.98E-04
Slc25a29	4.517	4.860	5.188	3.765	4.005	4.637	0.729	1.79E-02
Ptov1	7.863	7.908	8.006	7.232	7.156	7.159	0.729	8.64E-03
Tmem33	5.912	5.763	6.046	5.033	5.342	5.245	0.728	3.40E-03
LOC103692166	1.827	1.975	2.105	1.476	1.529	1.333	0.727	1.60E-02
Eaf1	4.333	4.452	4.895	3.637	3.950	4.047	0.726	4.84E-04
Lmnb2	4.239	4.290	4.722	3.593	3.730	3.872	0.726	3.59E-03
Mafk	3.494	3.850	4.433	2.919	3.327	3.601	0.722	3.47E-02
Baiap2	5.769	5.873	5.844	4.869	5.384	5.058	0.721	2.83E-04
Dus2	4.046	4.299	4.574	3.514	3.836	3.545	0.720	6.09E-03
Polr1c	5.790	5.919	6.270	5.229	5.135	5.426	0.719	1.72E-02
Fam110a	2.720	2.478	2.876	1.957	2.146	2.220	0.718	9.53E-03
Gas6	7.888	8.098	8.157	7.122	7.409	7.433	0.717	3.24E-04
Nutf2	6.027	5.977	6.304	5.305	5.383	5.502	0.717	2.89E-03
Paox	3.995	4.044	4.470	3.163	3.611	3.731	0.715	2.48E-03
Tmem165	5.389	5.474	5.818	4.666	4.927	5.051	0.712	4.11E-03
Umps	6.114	6.026	6.538	5.514	5.454	5.611	0.711	4.03E-03
Nmt2	7.417	7.234	7.537	6.739	6.698	6.637	0.709	1.82E-03
Rcc2	6.297	6.541	6.984	5.553	5.748	6.360	0.708	3.58E-02
Gpr45	1.672	1.957	2.221	1.456	1.443	1.479	0.708	4.47E-02
Vipr2	0.942	1.112	1.078	0.743	0.680	0.728	0.705	2.36E-02
Eml2	5.500	5.481	5.665	4.839	4.892	4.794	0.704	5.04E-04
RT1-CE6	3.217	3.726	3.837	2.591	3.310	2.935	0.703	3.71E-02

Gcsh	6.076	6.164	6.347	5.512	5.294	5.676	0.701	1.45E-02
Tbc1d10a	4.177	4.240	4.750	3.509	3.631	4.033	0.700	3.86E-03
Tmem120a	4.916	5.326	5.714	4.396	4.592	4.945	0.698	7.54E-03
Ngdn	6.462	6.649	6.967	5.884	6.105	6.066	0.695	1.65E-03
St6galnac6	5.244	5.253	5.727	4.649	4.669	4.872	0.695	2.14E-03
Fut10	3.350	3.311	3.317	2.609	2.777	2.773	0.694	1.28E-02
Rbm43	2.909	3.120	3.309	2.345	2.472	2.747	0.691	4.07E-03
Tp53bp1	7.112	7.003	7.232	6.483	6.478	6.299	0.690	3.31E-03
LOC498154	3.025	3.306	3.582	2.514	2.653	2.931	0.689	4.03E-03
Zfp64	3.641	3.790	4.316	3.297	3.151	3.432	0.688	4.11E-02
Gas8	3.439	3.363	3.833	2.627	3.006	3.165	0.686	2.47E-02
Mknk2	5.683	5.827	6.254	4.905	5.247	5.563	0.685	5.29E-03
Zfp717	5.812	5.983	6.215	5.201	5.447	5.404	0.684	7.17E-05
Larp7	5.919	5.732	5.943	5.248	5.106	5.265	0.683	1.32E-02
Ubl3	6.426	6.428	6.867	5.813	5.866	6.046	0.682	2.03E-03
Naa50	5.422	5.817	6.414	5.002	5.577	5.579	0.681	3.98E-03
Ttll5	4.238	4.154	4.290	3.517	3.731	3.545	0.680	2.18E-04
Pigh	3.894	3.872	4.185	3.247	3.364	3.478	0.679	1.05E-02
Phactr4	3.683	3.750	4.221	3.196	3.310	3.307	0.677	2.77E-02
Recql4	1.125	1.062	1.455	0.749	0.892	0.967	0.677	1.60E-02
RGD1310819	2.037	2.528	2.592	1.725	1.992	1.897	0.676	4.54E-02
Med21	6.452	6.545	6.722	5.624	6.030	6.123	0.671	6.51E-03
Cdc42ep4	4.317	4.362	4.695	3.588	4.032	3.826	0.671	1.44E-02
Srp54a	7.017	6.870	7.337	6.352	6.418	6.516	0.670	4.18E-04
Ntmt1	5.066	5.229	5.610	4.491	4.618	4.830	0.669	4.80E-03
Mettl17	4.274	4.714	4.778	3.695	3.982	4.191	0.668	5.43E-03
LOC361646	3.196	3.332	3.746	2.604	2.957	2.992	0.666	1.51E-02
Shisa5	6.170	6.442	6.988	5.648	6.028	5.965	0.666	1.06E-02
Suv39h2	3.091	3.257	3.433	2.700	2.679	2.518	0.666	1.09E-02

Rnf144b	1.937	2.231	2.480	1.514	1.897	1.864	0.666	2.07E-02
Ccdc32	5.368	5.401	5.756	4.794	4.906	4.896	0.665	8.88E-03
Chp1	6.416	6.445	6.813	5.933	5.862	5.911	0.664	4.09E-03
Ubxn4	6.605	6.687	6.951	5.982	6.135	6.205	0.660	1.17E-04
Zfyve26	3.532	3.653	4.003	2.992	3.283	3.123	0.657	8.29E-03
Napepld	2.590	2.771	3.170	1.914	2.498	2.452	0.657	4.70E-02
Sema4b	3.116	3.331	3.931	2.640	2.755	3.246	0.656	3.80E-02
Myt1l	5.571	5.486	6.021	4.813	5.066	5.310	0.654	4.66E-02
Stip1	8.172	7.929	8.119	7.393	7.307	7.583	0.654	2.01E-02
Lpgat1	5.493	5.371	5.828	4.807	4.909	5.105	0.653	2.31E-02
Mcm9	3.030	3.105	3.276	2.471	2.654	2.603	0.652	2.97E-04
Ino80e	5.112	5.201	5.643	4.607	4.604	4.838	0.651	1.13E-02
Spats2l	5.429	5.677	5.905	4.767	5.140	5.219	0.650	1.08E-03
Fchsd2	5.327	5.436	5.882	4.705	5.076	5.025	0.649	6.26E-04
Ppox	3.918	4.196	4.467	3.567	3.676	3.517	0.648	2.21E-02
Hyal3	4.112	4.379	4.325	3.611	3.692	3.684	0.648	2.06E-03
LOC500077	3.503	3.467	3.775	2.991	2.868	3.117	0.647	2.48E-02
Uba1	8.066	8.045	8.319	7.442	7.422	7.613	0.647	1.22E-03
Idnk	3.230	3.598	3.360	2.642	2.925	2.920	0.645	1.49E-02
Mrto4	5.320	5.556	5.781	4.654	4.849	5.241	0.644	1.12E-03
Xpo1	6.955	7.068	7.399	6.241	6.447	6.850	0.643	5.74E-03
Pnmal2	7.171	6.991	7.267	6.505	6.556	6.412	0.643	3.05E-03
Pdxk	6.106	6.327	6.238	5.582	5.738	5.371	0.643	2.37E-02
Gemin6	4.464	4.614	4.724	3.871	4.059	4.070	0.642	5.02E-03
Tspyl2	6.121	6.393	6.234	5.414	5.848	5.616	0.639	9.97E-03
Clk2	6.850	7.018	7.203	6.250	6.322	6.343	0.639	4.03E-03
Pigl	3.453	3.838	3.738	2.822	3.199	3.269	0.638	7.85E-03
Fam193a	5.385	5.482	5.846	4.863	5.011	4.999	0.638	1.91E-04
Wdr35	4.205	4.366	4.630	3.390	3.981	4.037	0.635	6.21E-03

Camk2g	6.023	6.061	6.117	5.482	5.578	5.131	0.635	1.82E-02
Lpin2	4.638	4.589	4.855	4.028	4.003	4.207	0.633	1.85E-03
Qsox1	4.136	4.243	4.638	3.638	3.871	3.695	0.633	1.85E-02
Adgrl2	5.267	5.739	5.936	4.768	5.048	5.318	0.633	8.32E-03
Hspa4l	5.879	5.866	5.915	5.291	5.304	5.189	0.632	5.30E-03
Dedd2	5.366	5.460	5.548	4.736	4.907	4.866	0.632	1.10E-03
Paip1	7.391	7.390	7.781	6.761	6.874	7.050	0.630	5.76E-04
Zfp174	1.832	1.878	2.200	1.419	1.583	1.592	0.630	4.60E-02
Adcy6	5.170	5.099	5.485	4.570	4.675	4.648	0.630	1.93E-04
Lsm1	5.456	5.423	5.770	4.810	4.974	5.045	0.629	2.73E-03
Akr1a1	9.504	9.508	9.733	9.006	8.834	8.981	0.629	2.19E-02
H2afx	5.673	5.733	6.154	5.052	5.218	5.438	0.628	1.53E-02
Ybx3	6.148	6.342	6.999	5.607	5.887	6.293	0.628	4.77E-02
Ero1a	5.243	5.128	5.523	4.544	4.674	4.891	0.628	1.49E-02
Prkra	6.262	6.408	6.763	5.788	5.882	5.941	0.627	9.11E-03
Prelid1	7.695	7.803	8.053	7.136	7.040	7.421	0.627	1.14E-02
Fbxl19	5.330	5.511	5.712	4.882	4.921	4.866	0.626	2.32E-03
Sorcs3	3.600	3.645	3.986	3.092	3.216	3.230	0.625	2.71E-03
Zswim8	5.083	4.931	5.307	4.406	4.371	4.711	0.624	9.12E-04
Syt6	4.118	4.279	4.323	3.294	3.919	3.763	0.623	1.74E-02
Atp2c1	7.333	7.502	7.532	6.731	6.962	6.833	0.623	7.45E-04
Mcm4	6.811	6.779	6.887	6.079	6.339	6.238	0.621	2.57E-04
Ppid	7.994	7.750	8.094	7.237	7.270	7.513	0.619	1.41E-03
Stim1	5.206	5.396	5.613	4.666	4.959	4.794	0.617	3.67E-04
Naglu	3.599	4.018	4.206	3.169	3.357	3.601	0.613	6.17E-03
Tiam2	3.348	3.584	4.057	3.054	2.992	3.321	0.611	2.28E-02
Zfp511	4.526	4.629	5.076	4.023	4.055	4.406	0.610	3.76E-02
Tube1	3.882	4.025	4.260	3.186	3.788	3.480	0.610	1.90E-02
Tmem200a	3.131	2.868	3.155	2.423	2.538	2.657	0.609	3.80E-02

Sufu	3.855	4.047	4.332	3.408	3.654	3.467	0.609	1.48E-02
Zmynd19	6.191	6.022	6.501	5.566	5.444	5.900	0.608	1.17E-02
Gpr68	5.285	5.415	5.953	5.005	4.876	4.997	0.606	1.96E-02
Maneal	5.299	5.338	5.795	4.836	4.875	4.951	0.603	7.04E-03
LOC102550456	4.437	4.399	4.640	3.529	3.734	3.828	0.602	3.46E-02
RGD1309995	6.315	6.354	6.576	5.628	5.975	5.901	0.601	3.07E-02
Cdan1	3.980	4.089	4.403	3.494	3.649	3.641	0.601	6.66E-03
Stk11ip	5.015	5.146	5.273	4.438	4.563	4.674	0.600	5.07E-03
Atg9a	6.529	6.604	6.786	6.015	6.163	5.915	0.600	8.90E-03
Nit1	6.500	6.690	6.714	6.038	6.191	5.856	0.599	2.85E-02
Ino80c	5.162	5.306	5.559	4.575	4.793	4.942	0.599	4.48E-04
Mfsd14b	5.012	5.044	5.409	4.365	4.684	4.713	0.599	5.33E-04
Cpped1	4.684	4.758	4.599	4.057	4.312	3.945	0.598	8.17E-03
Skp1	10.028	9.947	9.937	9.414	9.363	9.337	0.598	2.68E-02
Fndc4	5.472	5.744	6.147	5.207	5.224	5.201	0.596	1.96E-02
AABR07071395.1	4.275	4.194	3.996	3.327	3.664	3.805	0.594	3.36E-02
Dnttip1	6.052	6.286	6.348	5.549	5.741	5.689	0.594	8.88E-03
Creb3	6.535	6.739	7.076	6.210	6.159	6.205	0.592	1.61E-02
Dnaja1	9.203	9.055	9.201	8.498	8.524	8.704	0.592	1.43E-03
Kat7	6.515	6.624	6.925	6.064	6.103	6.154	0.592	8.59E-04
Tpm3	7.389	7.507	7.923	6.863	7.011	7.244	0.591	8.13E-03
Fam217b	2.724	2.857	3.409	2.332	2.767	2.561	0.591	1.16E-02
LOC100912481	7.769	7.955	8.082	7.157	7.412	7.449	0.591	1.56E-04
Ccdc25	5.661	5.489	5.967	4.980	5.059	5.376	0.590	1.57E-02
Gon4l	4.709	4.736	5.174	4.223	4.328	4.572	0.589	1.05E-02
Rnf41	6.900	6.981	7.375	6.445	6.514	6.575	0.589	3.36E-03
Gpn1	5.913	6.065	6.315	5.462	5.482	5.626	0.589	8.79E-04
Lysmd2	5.914	5.797	6.201	5.273	5.442	5.529	0.589	1.53E-02
Syt4	7.090	7.130	7.300	6.494	6.543	6.735	0.588	7.54E-03

Canx	8.926	8.829	9.133	8.203	8.389	8.568	0.588	4.19E-03
Bhlhe22	4.703	4.592	4.925	3.997	4.166	4.382	0.588	2.72E-02
Efhd2	5.692	5.946	6.208	5.104	5.447	5.549	0.587	1.24E-02
Sp110	5.021	5.295	5.489	4.591	4.851	4.658	0.587	4.09E-02
Cep83	3.828	3.770	3.722	4.352	4.333	4.370	-0.588	2.07E-02
LOC100911660	2.658	2.507	2.332	2.593	3.389	3.139	-0.591	3.91E-02
Reep1	5.509	5.556	5.433	6.095	6.049	6.113	-0.598	8.88E-03
Kndc1	2.143	1.974	2.213	2.472	2.640	2.663	-0.599	1.85E-02
Rad51d	3.220	3.440	3.356	3.774	4.016	3.855	-0.600	1.26E-02
Ppp1r14c	3.234	3.162	3.442	3.558	3.935	4.011	-0.603	4.30E-03
Rftn1	3.917	3.888	4.103	4.325	4.518	4.772	-0.604	4.19E-03
Sfrp2	5.126	4.987	5.015	5.602	5.555	5.758	-0.607	2.27E-02
Lmo4	4.637	4.946	4.994	5.167	5.530	5.586	-0.608	1.11E-03
Nrp2	4.346	4.471	4.103	4.798	4.978	4.930	-0.608	8.45E-03
Zfp608	3.051	3.028	3.044	3.595	3.550	3.630	-0.614	5.07E-03
St6galnac3	1.335	1.262	1.390	1.541	1.824	1.827	-0.617	4.27E-02
Ndrg1	3.909	4.222	4.108	4.677	4.669	4.804	-0.617	1.35E-02
Adcy5	2.592	2.617	2.636	2.953	3.283	3.202	-0.624	1.65E-03
B3gat1	4.227	4.227	4.217	4.777	4.775	4.897	-0.624	6.53E-03
St6gal1	3.209	3.194	3.576	3.663	3.840	4.221	-0.627	3.14E-03
Mtus1	6.753	6.609	6.489	7.453	7.324	7.256	-0.636	2.47E-02
Lrrk2	1.336	1.482	1.306	1.629	2.050	1.722	-0.636	4.65E-02
Sh3d19	3.362	3.719	3.955	4.210	4.548	4.506	-0.646	9.56E-04
Tspan2	3.145	2.804	3.250	3.646	3.566	3.799	-0.649	1.42E-02
Bub1	1.798	1.630	1.899	2.039	2.356	2.454	-0.652	2.75E-02
Grhpr	2.628	2.819	2.933	2.901	3.730	3.438	-0.654	2.70E-02
Dhrs1	3.210	3.702	3.194	3.793	4.161	3.981	-0.654	2.22E-02
Fhl2	1.736	1.952	2.143	2.149	2.392	2.796	-0.657	4.35E-02
Zfp40	4.579	4.463	4.338	4.967	5.203	5.229	-0.658	6.13E-03

Manba	3.154	3.291	3.057	3.674	4.026	3.608	-0.664	1.61E-02
Ifngr1	3.510	3.942	4.150	4.120	4.490	4.882	-0.675	7.00E-03
Ece2	2.198	2.241	2.274	2.588	2.926	2.857	-0.676	1.56E-03
Nacc2	2.209	2.358	2.368	2.550	3.040	3.094	-0.677	4.22E-02
Cdc25b	2.688	2.917	2.777	3.055	3.469	3.636	-0.680	5.90E-03
Tmem35b	2.004	2.007	2.393	2.453	2.739	2.902	-0.682	3.88E-02
Plppr3	3.416	3.893	4.257	4.098	4.525	4.885	-0.690	2.30E-02
Slc16a2	1.178	1.897	1.589	1.761	2.219	2.208	-0.692	4.27E-02
Mycn	1.289	1.460	1.517	1.630	1.893	2.133	-0.694	4.04E-02
Bmp1	3.437	3.808	3.833	3.832	4.595	4.534	-0.694	9.27E-03
Sybu	3.086	2.580	2.634	3.453	3.324	3.430	-0.695	4.35E-02
Zfp78	2.848	2.592	2.527	3.247	3.364	3.239	-0.697	1.27E-02
Slc35b2	3.815	4.008	3.923	4.411	4.591	4.710	-0.698	3.67E-04
Nrbp2	5.720	5.935	5.469	6.403	6.549	6.256	-0.698	1.89E-02
Slc25a27	4.974	4.620	4.624	5.537	5.445	5.323	-0.710	2.68E-02
Cgnl1	3.818	3.557	3.495	4.373	4.438	4.030	-0.711	3.16E-02
Impa2	2.984	3.670	3.469	3.639	4.187	3.901	-0.713	1.18E-02
Cryl1	2.495	2.683	2.303	2.805	3.450	3.057	-0.713	4.30E-02
Adgrb2	3.577	3.478	3.844	3.871	4.204	4.741	-0.714	2.15E-02
Adamts10	3.059	3.308	2.801	3.391	3.885	3.587	-0.728	1.56E-02
C1qtnf3	3.484	3.384	3.129	3.970	4.077	4.012	-0.729	1.03E-02
Anapc16	5.056	5.019	5.036	5.654	5.794	5.818	-0.731	8.58E-04
Nim1k	1.241	1.873	1.291	1.689	2.184	2.179	-0.744	4.93E-02
Sertm1	1.878	2.090	1.797	2.180	2.731	2.622	-0.746	1.08E-02
Sncaip	3.142	3.029	3.190	3.611	3.740	4.070	-0.750	2.32E-03
Sertad4	3.099	2.820	3.041	3.548	3.693	3.724	-0.755	1.09E-03
Gadd45a	5.042	5.384	5.097	5.451	6.262	6.030	-0.759	4.87E-03
Elmod1	4.172	4.129	3.972	4.872	4.916	4.698	-0.760	6.19E-03
Osbpl5	1.622	1.612	2.015	1.928	2.399	2.663	-0.765	2.23E-02

Gas2l1	2.137	1.950	2.264	2.512	2.729	2.962	-0.766	1.09E-02
Myo1b	2.657	3.036	3.154	3.427	3.741	3.783	-0.769	3.46E-03
Itgad	1.499	1.944	1.830	2.141	2.471	2.429	-0.770	4.84E-02
Prkar2b	4.682	4.779	4.844	5.364	5.244	5.936	-0.774	2.65E-03
Glrx	5.426	5.418	5.204	6.019	6.373	5.982	-0.782	8.74E-03
Tex14	2.536	2.309	2.480	3.181	3.057	3.107	-0.784	2.32E-02
Rgs10	4.173	4.651	4.226	5.103	5.218	5.046	-0.789	3.80E-02
Fbxo44	4.144	4.331	3.921	4.806	5.119	4.736	-0.791	3.11E-02
Cdh20	3.133	3.207	3.179	3.748	3.828	4.116	-0.793	1.56E-03
Nxph4	2.612	3.008	3.220	3.269	3.755	3.912	-0.794	3.48E-02
Fam163a	1.586	1.494	1.455	2.096	2.216	1.863	-0.796	9.51E-03
Gls2	2.756	2.912	2.917	3.653	3.657	3.328	-0.798	2.44E-03
Cttnbp2	2.715	2.946	3.022	3.204	3.843	3.768	-0.802	9.28E-03
Scn1a	3.738	3.759	3.225	4.167	4.620	4.276	-0.808	4.22E-02
LOC497940	2.613	2.959	2.520	3.290	3.687	3.258	-0.809	4.98E-02
Cldn11	3.135	3.009	3.192	3.632	3.856	4.055	-0.811	1.84E-02
Serpini1	4.520	4.709	4.415	5.250	5.569	5.230	-0.814	1.76E-03
Chrd	1.727	1.825	1.741	2.370	2.405	2.356	-0.819	2.81E-03
Ctgf	1.998	2.435	1.787	2.410	2.830	3.042	-0.820	4.67E-02
LOC103692165	1.631	1.538	1.856	2.044	2.340	2.473	-0.826	8.86E-03
Bcas1	4.168	4.197	4.242	4.636	4.966	5.354	-0.830	5.59E-04
Trpv2	2.059	2.204	2.634	2.798	2.949	3.237	-0.830	9.81E-03
Camk1g	0.915	1.308	1.143	1.294	1.797	1.656	-0.838	3.92E-02
Nfkbie	2.924	3.185	3.428	3.637	3.943	4.254	-0.848	1.18E-03
Smad9	1.213	1.428	1.480	1.637	2.056	2.157	-0.850	9.51E-03
LOC100359539	1.772	1.921	2.045	2.176	2.682	2.877	-0.852	1.74E-02
Tspan12	3.430	3.886	3.834	4.040	4.560	4.970	-0.863	7.66E-03
Dlc1	2.769	3.043	2.923	3.355	3.849	3.991	-0.865	1.19E-03
RGD1311575	2.566	2.661	3.158	3.044	3.647	3.994	-0.874	2.92E-02

Slc8a1	3.048	2.898	3.291	3.765	3.828	4.047	-0.876	2.93E-02
Olig1	2.962	2.829	3.144	3.398	3.868	4.015	-0.879	1.62E-02
Mfsd4	2.763	2.988	2.625	3.584	3.825	3.317	-0.882	1.23E-02
Rarb	2.191	1.565	1.827	2.671	2.222	2.832	-0.895	3.38E-02
Bmp4	0.891	0.947	1.084	1.493	1.296	1.634	-0.896	4.50E-02
Slitrk3	1.273	1.402	1.492	2.118	2.045	1.804	-0.898	3.65E-02
Podxl	1.076	1.734	1.587	1.736	2.134	2.175	-0.899	8.29E-03
Tmem200c	1.866	1.989	1.976	2.326	2.706	2.903	-0.900	1.48E-03
Nrep	6.952	7.230	6.744	7.838	8.157	7.725	-0.924	2.35E-02
Adamtsl4	0.713	1.110	0.939	1.109	1.707	1.458	-0.931	3.35E-02
Ccdc177	1.054	1.006	0.967	1.253	1.754	1.653	-0.932	2.92E-02
43170	1.275	1.166	1.139	1.788	1.824	1.788	-0.939	2.78E-02
Stmn4	6.589	6.248	6.522	7.451	7.286	7.378	-0.941	2.77E-02
Tmem229a	1.267	1.402	0.874	1.846	1.792	1.823	-0.950	2.41E-02
Kif22	1.452	1.776	2.016	2.043	2.415	2.928	-0.952	1.17E-02
Sytl2	0.953	1.470	1.128	1.592	2.104	1.811	-0.958	3.22E-02
Thsd7b	0.848	1.330	0.954	1.299	1.639	1.967	-0.975	3.38E-02
Mdga2	2.021	2.480	2.233	2.708	3.264	3.260	-0.980	3.23E-02
Samd11	2.063	2.548	2.003	2.806	3.289	3.037	-0.992	9.47E-03
AABR07036331.2	1.465	1.859	1.497	2.268	2.334	2.456	-0.992	1.52E-02
Rasgrf2	1.291	1.147	1.376	1.825	2.012	2.019	-1.011	6.55E-03
Tshr	0.781	0.869	0.883	1.383	1.323	1.434	-1.018	1.34E-03
Rfx8	0.515	0.531	0.614	0.710	1.279	0.900	-1.032	4.62E-02
Gpr22	1.070	0.979	1.063	1.435	1.992	1.579	-1.035	1.48E-02
Sema3g	1.220	1.450	1.189	1.569	2.526	1.701	-1.037	4.35E-02
Cpne7	2.503	2.091	2.152	2.800	2.938	3.595	-1.042	8.78E-03
LOC100910438	2.397	2.491	1.906	3.169	3.136	3.331	-1.077	9.42E-03
Slc6a7	1.426	1.432	1.679	2.010	2.439	2.340	-1.095	1.86E-02
P2rx2	1.502	1.936	1.807	2.016	2.672	3.258	-1.103	1.22E-02

Spc25	0.980	1.391	1.649	1.674	2.384	2.305	-1.109	3.39E-02
Hey1	1.650	1.572	1.741	2.111	2.660	2.773	-1.150	6.45E-03
Cdkn2a	1.362	2.318	1.876	2.550	2.941	3.087	-1.211	1.46E-02
NEWGENE_621438	0.263	0.552	0.457	0.569	0.924	1.044	-1.213	4.20E-02
Cdca3	0.971	0.903	1.330	1.655	1.843	2.037	-1.232	3.22E-02
Ldb2	0.251	0.208	0.413	0.599	0.704	0.684	-1.289	4.77E-02
Spag6l	0.594	0.332	0.518	0.708	1.151	1.132	-1.295	4.68E-02
Trib3	1.198	1.701	1.998	2.240	2.811	3.057	-1.390	8.18E-04
Akr1b10	3.363	3.928	2.763	4.423	5.261	4.908	-1.395	3.36E-02
Iqcd	0.611	0.900	0.708	1.238	1.582	1.663	-1.410	3.15E-02
Tagln	0.670	1.671	1.736	1.973	2.502	3.059	-1.547	3.10E-02
Grip2	0.511	0.636	0.467	1.261	1.158	1.150	-1.607	4.41E-04
Myo1f	0.231	0.210	0.383	0.546	0.745	1.070	-1.804	8.39E-03
Etfbkmt	0.615	0.823	0.769	1.101	2.016	2.111	-1.845	7.28E-03
Depdc1	0.142	0.099	0.463	0.694	0.864	0.639	-1.860	4.68E-02
Cyp1a1	0.234	0.646	0.581	1.023	1.718	1.519	-2.048	1.46E-02
Scn5a	0.098	0.088	0.294	0.252	0.637	0.620	-2.128	3.58E-02
RGD1309870	0.384	0.559	0.456	0.566	1.983	2.552	-2.248	4.72E-02
Mmp13	0.198	0.337	0.290	0.690	1.453	0.893	-2.282	3.40E-03
Slc40a1	0.300	0.304	0.576	0.555	1.449	1.647	-2.354	2.14E-02
Mmp3	0.727	0.884	2.035	2.603	3.840	2.786	-2.447	1.88E-02
Hist1h2an	0.742	1.189	1.998	2.040	3.613	3.843	-2.448	8.02E-03
Mmp10	0.138	0.000	0.318	0.518	1.073	0.848	-2.722	1.60E-02
AABR07057333.1	0.000	0.000	0.000	1.183	0.000	2.490	-7.122	3.06E-02

Table S2. HSF1-regulated genes in apoptotic CGNs

Expression level (log2-transormed) of the HSF1 targets under LK condition in HSF1-expressing and GFP-expressing CGNs is listed along with log2 [fold change] and FDR. Novel targets of HSF1 are in blue.

Table S2. HSF1-regulated genes in apoptotic CGNs

Genes	HSF1 LK-1	HSF1 LK-2	HSF1 LK-3	GFP LK-1	GFP LK-2	GFP LK-3	Log2[fold change]	FDR
AABR07069837.1	4.126	4.148	4.427	0.274	0.000	0.000	7.155	4.27E-05
Rbm12	2.689	3.843	3.793	0.142	0.081	0.140	6.748	3.05E-02
AABR07016950.1	5.559	5.754	6.668	0.000	1.241	1.068	6.335	1.40E-03
AABR07069913.1	3.659	3.022	3.685	0.000	0.000	0.485	6.245	1.41E-03
AABR07069821.1	3.263	3.520	4.327	0.157	0.411	0.000	6.217	1.40E-04
Dmrt1	1.410	1.275	2.791	0.114	0.000	0.000	5.998	2.24E-03
RGD1560554	2.776	2.809	2.706	0.182	0.252	0.000	5.501	1.58E-03
LOC102546495	2.879	3.044	3.709	0.254	0.375	0.000	5.376	4.34E-03
LOC100360908	1.452	2.153	2.995	0.072	0.000	0.281	5.321	7.30E-03
AABR07016976.1	6.045	6.156	7.013	1.500	1.608	2.105	5.122	2.54E-04
Acan	1.009	1.109	1.595	0.036	0.067	0.066	5.117	1.20E-04
Slc34a2	1.263	1.463	2.521	0.111	0.154	0.026	5.060	3.14E-04
LOC102546376	5.195	5.555	6.129	1.669	0.797	1.241	4.940	8.31E-03
Zfp93	2.526	2.216	3.682	0.188	0.397	0.347	4.730	4.23E-05
Alx4	2.596	2.763	3.674	0.089	0.488	0.482	4.705	1.15E-03
Pax7	2.954	2.643	3.979	0.209	0.470	0.572	4.644	2.54E-04
AABR07060481.1	2.797	2.874	3.262	0.148	0.000	0.801	4.601	2.23E-02
LOC102550797	3.392	3.047	3.990	0.856	0.426	0.000	4.463	7.92E-03
Apob	0.535	0.343	0.600	0.007	0.067	0.010	4.359	1.83E-02
AABR07016919.1	4.318	4.213	5.037	0.813	1.446	1.313	4.080	1.15E-03
Fbxw10	0.939	0.852	1.386	0.058	0.122	0.082	4.054	1.15E-03
RGD1562660	5.902	5.525	6.864	2.543	0.000	3.021	4.035	4.34E-03
Nmb	4.949	5.334	5.745	1.738	1.502	1.981	4.012	3.22E-07
Igfbpl1	4.269	4.195	5.171	0.994	1.308	1.500	3.989	8.04E-05
Plac9	1.958	3.121	3.217	0.221	0.766	0.429	3.903	1.12E-03
AABR07068094.2	2.287	2.871	3.582	0.342	0.870	0.465	3.828	8.09E-03
Batf	1.722	1.479	1.866	0.105	0.280	0.276	3.648	4.30E-03

Crhr2	1.868	2.219	3.539	0.430	1.161	0.571	3.569	8.55E-03
AABR07016992.1	4.372	3.803	4.466	1.398	1.509	0.936	3.501	4.34E-03
Cryab	7.213	7.308	7.342	3.718	3.992	3.717	3.497	2.38E-06
Sctr	1.857	1.842	2.315	0.244	0.384	0.425	3.389	1.20E-04
Spetex-2F	4.128	4.174	5.262	1.162	1.842	1.669	3.380	1.06E-03
Sox15	2.558	2.314	3.495	0.388	0.781	0.766	3.373	2.08E-03
Ccdc180	0.565	0.709	1.120	0.020	0.186	0.108	3.333	1.03E-02
Amz1	3.162	3.234	4.044	0.836	1.192	1.000	3.329	2.38E-05
LOC102551549	1.652	1.744	2.409	0.109	0.533	0.524	3.194	1.54E-02
Sash3	1.043	0.730	1.604	0.099	0.267	0.181	3.157	1.28E-02
Anxa3	2.136	2.368	2.746	0.518	0.405	0.788	3.097	1.06E-03
Rfx6	1.133	1.250	2.704	0.245	0.446	0.440	3.093	7.74E-03
Gli1	0.586	0.859	1.266	0.090	0.126	0.193	3.092	4.33E-03
Chrnd	0.717	0.990	1.225	0.051	0.263	0.136	3.085	1.03E-02
Ntf4	0.712	0.971	1.613	0.062	0.170	0.318	3.068	2.77E-02
Kank4	3.521	3.433	4.208	1.118	1.617	1.054	3.067	8.06E-05
Esr1	2.822	2.808	3.573	0.609	1.247	0.655	3.063	4.48E-03
AABR07016947.1	2.143	1.859	2.713	0.000	1.106	0.389	3.056	4.69E-02
Nipal1	0.238	0.888	0.825	0.048	0.198	0.101	3.053	4.30E-02
Fezf2	1.416	1.948	3.052	0.377	0.706	0.497	3.041	9.75E-03
LOC100912034	1.401	1.036	2.298	0.244	0.194	0.514	3.031	3.92E-02
Nodal	1.333	1.373	1.860	0.220	0.514	0.106	3.031	2.79E-02
Ovol2	1.989	1.978	3.434	0.456	0.915	0.687	3.024	1.65E-02
Cyp2s1	2.558	2.873	3.723	0.659	1.063	1.090	3.009	5.10E-04
Adgrg2	2.965	2.937	3.792	0.816	1.141	1.172	3.004	2.63E-05
Muc5b	0.259	0.454	0.541	0.031	0.068	0.075	2.992	5.08E-03
Madcam1	3.429	4.151	3.944	1.178	1.772	1.323	2.914	4.27E-05
Taf7l	1.492	1.599	2.032	0.200	0.192	0.710	2.913	3.56E-02
Fam83a	2.251	2.261	3.219	0.484	1.015	0.728	2.897	1.15E-03

Prss16	0.548	0.844	1.468	0.114	0.301	0.106	2.851	9.75E-03
Ccdc169	0.718	1.350	1.343	0.106	0.222	0.354	2.808	3.21E-02
Hspb1	6.497	6.744	7.601	3.842	4.179	4.499	2.798	6.45E-04
Cilp	0.325	0.314	0.778	0.040	0.163	0.028	2.787	3.49E-02
Vwf	2.010	2.141	2.933	0.399	0.805	0.862	2.776	1.97E-04
Myo1h	1.596	1.318	1.867	0.184	0.647	0.282	2.774	2.32E-03
Melk	3.086	3.333	4.280	1.153	1.340	1.713	2.759	4.39E-04
RT1-M4	1.787	2.722	2.891	0.368	1.178	0.588	2.751	1.82E-02
AABR07043453.1	6.825	6.579	7.504	4.570	4.377	3.522	2.743	8.96E-04
Slco1c1	1.142	1.210	1.571	0.068	0.227	0.556	2.740	4.57E-03
AABR07072112.1	2.042	2.199	3.133	0.538	1.024	0.715	2.732	1.09E-02
Bmpr1b	1.782	1.655	2.316	0.196	0.873	0.442	2.706	1.97E-02
Slc6a13	0.960	1.164	1.432	0.193	0.276	0.262	2.692	4.15E-03
Baiap2l1	2.383	2.498	3.339	0.540	1.010	1.157	2.688	5.00E-04
AABR07005779.3	1.323	1.624	2.178	0.421	0.423	0.417	2.687	9.91E-03
Slc46a2	0.825	1.062	1.610	0.079	0.396	0.303	2.681	3.33E-02
Cntnap2	6.254	6.332	6.523	4.608	3.878	3.859	2.622	5.08E-03
Pik3ap1	1.654	1.743	2.675	0.354	0.855	0.575	2.599	3.14E-03
AABR07060473.1	1.632	1.796	2.147	0.402	0.685	0.451	2.597	2.15E-02
Adcy7	2.885	2.948	3.726	0.864	1.355	1.466	2.592	6.24E-04
Popdc3	3.266	2.925	3.898	1.306	1.310	1.445	2.558	8.96E-04
Tmem106a	2.685	3.059	3.745	0.688	1.486	1.508	2.555	5.45E-03
Vav1	1.600	1.967	2.422	0.204	0.689	0.890	2.549	2.04E-02
Tchh	4.298	4.636	5.449	1.897	2.998	2.566	2.543	3.16E-04
Scd	3.524	3.720	4.600	1.282	2.043	2.110	2.533	2.40E-04
Gsdmd	4.291	4.705	5.266	1.830	2.854	2.662	2.525	1.56E-03
Crabp1	5.094	5.165	5.533	3.122	2.904	2.370	2.503	1.06E-03
Tmem37	1.032	1.182	1.878	0.383	0.384	0.238	2.490	5.08E-03
Edaradd	2.369	2.570	3.644	0.444	1.496	1.358	2.485	1.72E-02

AC139642.1	2.146	1.688	2.852	0.478	0.642	1.136	2.461	2.44E-02
Ddx43	2.589	2.642	3.360	1.001	1.333	0.968	2.459	9.65E-03
Bag3	5.557	5.640	6.232	3.077	3.498	3.680	2.449	4.39E-04
Tmem102	0.701	0.811	1.290	0.095	0.254	0.306	2.439	5.87E-03
AABR07044980.1	1.012	1.036	1.623	0.125	0.511	0.323	2.418	3.81E-02
Matn4	2.599	3.099	3.785	1.334	1.226	1.425	2.395	1.02E-02
Acp5	2.898	3.239	3.800	1.156	1.816	1.340	2.389	1.60E-03
Ikzf3	1.206	0.921	1.742	0.188	0.352	0.551	2.343	9.91E-03
Krt13	2.346	2.118	3.183	0.605	1.252	1.089	2.335	2.19E-02
Plod1	4.736	5.298	5.793	2.393	3.408	3.379	2.321	2.00E-03
LOC102547811	4.638	4.673	5.397	2.932	2.878	2.280	2.313	3.45E-03
Zfp185	3.675	3.613	4.456	1.517	2.335	1.905	2.307	8.96E-04
Mcoln2	0.582	0.890	1.371	0.210	0.414	0.102	2.295	4.22E-02
Kdelr3	5.702	5.600	6.434	3.498	3.720	4.055	2.294	1.06E-03
Pde4c	1.071	0.944	1.380	0.265	0.237	0.396	2.287	3.69E-02
Zfp663	2.225	2.461	3.355	0.657	1.661	0.907	2.276	8.70E-03
Egfem1	1.032	1.276	1.555	0.342	0.285	0.463	2.267	1.03E-02
Fosb	3.571	3.781	4.378	1.811	1.832	2.124	2.266	6.24E-04
Plekhg3	6.149	6.480	6.948	4.018	4.554	4.270	2.258	9.14E-07
Trim16	2.534	2.643	3.402	1.074	1.387	1.224	2.215	3.14E-04
Mum1l1	2.401	2.042	2.905	0.722	1.123	1.130	2.207	2.54E-04
Esr2	2.429	2.192	3.206	0.431	1.336	1.289	2.202	2.82E-02
AABR07000740.1	9.303	8.992	10.064	7.502	7.171	7.010	2.190	3.53E-04
Casq2	0.885	0.732	1.394	0.350	0.258	0.207	2.189	3.67E-02
RT1-M5	2.736	3.068	3.575	1.144	1.839	1.145	2.188	1.78E-02
LOC363337	7.412	7.350	8.113	5.592	5.474	5.267	2.182	2.60E-04
Lad1	2.692	2.594	3.533	1.125	1.113	1.619	2.181	1.64E-02
Dnai1	1.441	1.920	2.051	0.581	0.772	0.473	2.181	1.94E-02
Gjb2	2.021	1.983	2.614	0.660	0.957	0.889	2.181	2.24E-03

Krt42	1.162	1.196	1.975	0.330	0.630	0.444	2.176	3.32E-02
AABR07043276.1	6.427	6.295	7.007	4.811	4.399	3.658	2.176	4.08E-03
Lyve1	1.134	1.486	1.744	0.185	0.663	0.565	2.163	2.44E-02
Btn1a1	1.002	1.061	1.534	0.316	0.463	0.297	2.153	8.10E-03
Kcnk4	2.228	2.100	2.787	0.656	1.091	1.084	2.135	8.76E-03
Lyzl4	3.178	3.158	4.310	1.612	1.876	1.947	2.126	2.39E-02
RGD1311892	1.327	1.417	2.388	0.470	0.626	0.748	2.122	9.60E-03
Comp	3.146	3.439	3.826	1.344	1.733	1.654	2.109	2.21E-03
Smpdl3b	2.742	3.139	3.509	1.296	1.728	1.352	2.093	3.14E-03
Rn60_20_0054.5	4.146	4.342	5.443	2.149	3.130	2.938	2.078	6.30E-03
Cyp2d4	1.450	1.739	2.124	0.390	0.773	0.770	2.066	1.59E-03
Glt8d2	2.655	2.661	3.052	0.665	1.572	1.451	2.061	8.09E-03
Dsg2	2.141	2.114	2.991	0.719	1.138	1.337	2.013	8.30E-03
Mro	3.123	2.897	3.779	1.402	1.722	1.656	2.012	3.16E-02
Nlrp6	4.228	4.296	5.347	2.004	2.943	3.335	2.011	4.82E-02
Eya4	1.611	1.592	2.320	0.309	1.050	0.784	1.994	2.16E-02
Lmod3	1.093	1.158	1.576	0.544	0.480	0.177	1.988	4.16E-02
Aox1	3.725	3.765	4.533	1.125	2.728	3.151	1.985	1.63E-02
Mypn	0.284	0.738	0.893	0.116	0.211	0.246	1.976	4.82E-02
Bik	2.489	2.481	3.680	1.096	1.664	1.567	1.959	1.00E-02
Tacr2	1.834	1.675	2.129	0.475	1.034	0.700	1.957	2.91E-02
Il25	2.341	2.966	3.509	1.216	1.608	1.510	1.955	1.02E-02
Fam110d	3.016	3.018	4.106	1.676	1.733	1.944	1.952	1.89E-02
Rp1l1	0.801	0.702	1.305	0.278	0.323	0.305	1.951	4.83E-02
Ly6l	1.677	1.939	3.595	1.318	1.124	1.850	1.938	2.31E-02
Greb1	2.584	3.241	2.996	0.951	1.832	1.492	1.922	2.33E-03
Nckap1l	1.030	1.350	1.756	0.365	0.557	0.564	1.921	1.05E-02
Arhgef26	1.839	2.045	2.725	0.754	0.908	1.227	1.913	3.49E-02
Btc	2.898	3.014	3.823	1.310	1.799	2.048	1.911	1.57E-03

Plxdc1	3.087	3.228	3.582	1.710	1.887	1.466	1.905	8.96E-04
Ptpro	5.087	4.852	5.599	3.359	3.014	3.669	1.874	1.93E-03
AABR07069816.1	2.265	2.219	2.651	0.983	1.125	1.171	1.872	2.84E-03
Hspb2	2.234	2.625	2.427	1.089	1.484	0.528	1.849	3.00E-02
Aldh1a2	3.544	3.162	3.608	2.078	1.752	1.647	1.847	1.97E-02
Prokr2	1.793	2.335	1.557	0.675	0.638	1.065	1.843	3.92E-02
Acvrl1	1.304	1.707	2.329	0.712	0.814	0.735	1.825	2.72E-02
Prom2	1.409	1.442	1.940	0.563	0.622	0.718	1.824	6.45E-04
Cdh23	1.160	1.230	1.666	0.244	0.750	0.589	1.801	1.72E-02
LOC108348293	5.982	5.790	6.661	4.486	4.783	3.703	1.792	9.75E-03
Sypl2	5.091	5.169	5.451	3.490	3.878	2.918	1.773	3.96E-03
Calcr	0.805	0.788	1.159	0.217	0.428	0.359	1.771	1.78E-02
AABR07043456.1	5.237	4.823	5.686	3.623	3.110	3.781	1.763	1.46E-02
Kcns3	1.453	1.819	2.508	0.668	1.159	0.803	1.755	4.31E-02
Myh7	0.810	0.964	1.393	0.370	0.364	0.454	1.740	2.05E-02
Sec14l5	1.093	1.103	1.820	0.403	0.471	0.761	1.726	3.81E-02
Myoz1	1.435	2.009	1.630	0.587	0.700	0.919	1.715	3.63E-02
Otop3	1.817	1.761	2.361	1.036	0.890	0.698	1.713	1.69E-02
Troap	1.964	1.970	2.638	0.555	1.054	1.473	1.710	2.72E-02
C1ql2	3.277	3.283	4.399	2.108	2.178	2.355	1.704	2.18E-02
Slc9a2	1.604	1.656	2.273	0.750	1.041	0.706	1.702	1.18E-02
Morc4	3.945	4.031	4.860	2.399	2.989	3.129	1.697	2.08E-03
Card14	2.062	2.175	2.994	0.864	1.383	1.432	1.693	1.19E-02
Calr4	2.545	2.413	3.606	1.265	1.691	1.929	1.689	4.84E-02
Pmaip1	6.093	5.994	6.912	4.403	4.883	4.935	1.667	6.12E-03
Ociad2	6.720	6.490	6.908	4.994	5.191	4.893	1.659	2.40E-04
Cib2	4.002	3.787	4.414	2.558	2.578	2.492	1.651	4.57E-03
Bst2	8.419	8.887	9.353	6.280	7.611	7.707	1.650	4.75E-02
LOC102551901	4.798	4.238	3.691	2.796	2.877	2.650	1.641	2.36E-02

Lrrc38	2.827	2.995	3.389	1.675	1.863	1.544	1.641	1.15E-03
Pygm	3.233	3.703	3.427	2.083	2.233	1.676	1.636	2.58E-02
Ccdc158	3.767	3.739	4.381	2.178	2.568	2.737	1.634	6.82E-03
Accsl	1.774	1.906	2.252	0.864	0.870	1.071	1.631	7.30E-03
Alpk2	0.944	0.863	1.307	0.387	0.482	0.375	1.613	9.17E-03
Lamc2	4.570	4.772	4.689	2.739	3.683	2.723	1.608	1.44E-03
Sytl5	2.369	2.440	2.857	0.685	1.879	1.480	1.608	2.60E-02
Zfp575	2.363	2.135	2.663	1.456	0.852	1.078	1.604	3.82E-02
Intu	2.880	3.244	3.985	1.453	2.563	2.293	1.590	2.89E-02
Eras	1.407	1.703	2.131	0.704	0.919	0.844	1.588	8.44E-03
Saxo2	1.024	1.484	1.544	0.394	0.838	0.596	1.582	4.51E-02
Gsdma	3.589	3.776	4.099	1.890	2.757	2.520	1.579	3.14E-04
Gpr132	1.481	1.483	2.558	0.633	0.982	1.167	1.570	4.76E-02
Samd5	1.872	2.116	2.536	0.404	1.383	1.513	1.564	3.21E-02
Dennd2d	1.224	1.329	1.791	0.734	0.532	0.620	1.562	2.30E-02
Gck	1.486	1.765	2.440	0.702	1.035	1.135	1.546	1.65E-02
Phf11	2.970	3.293	3.218	1.495	1.959	2.163	1.541	2.21E-02
Palm2	3.028	2.884	3.518	1.225	2.229	2.124	1.524	4.57E-03
Pik3r5	1.894	2.016	2.493	0.787	1.294	1.251	1.506	1.54E-02
Fgd5	1.124	1.098	1.732	0.333	0.901	0.589	1.498	4.33E-02
Lrrc46	3.505	3.678	4.302	2.383	2.422	2.563	1.497	2.09E-03
Thbs4	1.622	1.664	2.351	0.879	0.914	1.147	1.494	1.22E-02
Scml4	1.906	1.712	2.656	0.659	1.241	1.426	1.490	3.71E-02
Crispld2	5.399	5.785	5.930	3.422	4.571	4.521	1.486	7.30E-03
Dmkn	2.077	2.487	2.823	1.460	1.339	1.560	1.485	1.89E-02
Phf11b	4.451	4.616	5.147	2.973	3.228	3.701	1.471	1.75E-02
Rasa4	5.383	5.760	5.541	4.226	4.229	3.687	1.458	6.23E-03
Angptl1	1.062	1.292	1.735	0.626	0.940	0.481	1.447	3.54E-02
Slco2b1	0.605	0.512	0.822	0.318	0.261	0.207	1.443	4.53E-02

LOC679342	2.902	2.647	3.669	1.884	1.970	1.853	1.425	3.11E-02
Prss56	2.383	2.329	2.673	1.273	1.588	1.307	1.416	9.75E-03
Anxa4	5.084	5.371	5.755	3.425	4.187	4.198	1.412	6.58E-04
Lingo3	4.493	4.664	5.023	3.065	3.500	3.465	1.407	2.84E-03
AABR07043358.1	6.761	6.457	7.290	5.729	5.352	5.130	1.398	1.94E-02
Grb7	2.511	2.723	2.967	1.405	1.912	1.503	1.388	1.04E-02
AABR07042361.1	6.676	6.992	6.843	4.447	5.473	6.155	1.388	1.58E-02
Fam126a	4.227	4.230	5.151	2.243	3.548	3.814	1.378	4.82E-02
Parp3	4.406	4.662	5.200	2.915	3.621	3.743	1.378	1.28E-02
LOC100910996	5.851	5.852	6.312	4.848	4.437	4.459	1.375	3.78E-03
Fignl1	2.872	2.887	3.652	1.731	2.178	2.153	1.369	2.90E-03
Lrrc3c	3.431	3.200	3.506	1.544	2.147	2.747	1.369	3.56E-02
Bfsp2	1.331	1.725	1.913	0.783	0.971	0.828	1.361	1.65E-02
Sec31b	1.444	1.239	1.840	0.500	0.910	0.912	1.358	8.30E-03
Wnk4	1.520	1.554	2.350	0.898	1.082	0.979	1.357	3.33E-02
Mns1	3.864	3.815	4.709	2.362	3.246	3.238	1.345	1.02E-02
Hsph1	7.947	7.922	8.467	6.362	6.741	7.139	1.338	1.90E-03
Epha6	2.755	2.732	2.747	1.126	2.016	1.869	1.338	8.30E-03
RT1-DMa	4.995	5.276	5.674	3.019	4.466	4.307	1.326	8.31E-03
Arhgef28	2.469	3.004	2.804	1.148	1.921	1.984	1.324	5.98E-03
AABR07042611.1	8.690	8.492	9.351	7.781	7.326	7.273	1.324	8.30E-03
Mcf2	2.004	2.240	2.863	1.133	1.609	1.547	1.319	3.11E-02
Heatr5a	3.607	3.654	4.483	2.013	2.998	3.141	1.302	1.86E-02
LOC497899	4.132	4.193	4.884	2.992	3.404	3.144	1.300	2.71E-02
C2cd2	3.533	3.313	3.932	1.998	2.600	2.550	1.298	1.33E-03
Mpdz	4.586	4.544	5.125	2.799	3.669	4.021	1.292	2.30E-03
Krt7	4.591	4.172	4.923	3.446	3.353	2.949	1.274	1.86E-02
AABR07025272.1	3.574	3.195	2.942	1.553	2.671	2.249	1.273	2.38E-02
Gstk1	3.700	3.636	4.204	2.469	2.732	2.785	1.272	1.01E-03

AABR07058884.1	1.757	1.684	1.904	0.749	1.066	1.106	1.264	4.08E-03
Thrb	3.592	3.600	4.260	2.222	2.847	2.863	1.260	1.16E-02
Fhl3	3.798	3.918	4.533	2.718	3.059	2.925	1.252	3.88E-02
Cers3	0.836	1.076	1.031	0.437	0.417	0.831	1.252	4.16E-02
Asic2	4.832	4.778	4.948	3.604	3.501	3.762	1.226	1.99E-03
Osbpl3	3.260	3.246	3.890	1.991	2.397	2.753	1.225	5.55E-03
LOC501421	4.938	5.190	5.698	4.405	3.804	3.745	1.224	4.69E-02
LOC498265	2.881	3.193	3.807	2.012	2.399	2.402	1.196	7.30E-03
Ccdc159	2.571	2.682	3.245	1.669	1.903	1.878	1.180	8.09E-03
Ulk3	3.546	3.577	4.212	2.460	2.831	2.822	1.174	1.12E-03
Lman2l	6.674	6.507	6.951	5.471	5.465	5.529	1.173	1.28E-03
Herc6	4.860	5.115	5.476	3.296	4.402	4.261	1.173	4.16E-02
Gpr3	3.368	3.548	3.913	2.307	2.596	2.520	1.172	5.13E-03
Syt5	7.299	7.181	7.693	6.388	5.759	6.201	1.172	1.93E-02
Tmem130	8.437	8.290	8.286	7.403	7.010	6.739	1.164	4.68E-02
Lin7b	4.662	4.699	5.003	3.830	3.308	3.637	1.150	4.26E-02
Rnf213	4.804	4.691	5.061	2.661	4.097	4.204	1.141	3.63E-02
Lgals9	8.768	8.638	9.028	7.560	7.373	7.790	1.137	4.34E-03
B3galnt2	4.008	4.187	5.066	2.879	3.495	3.740	1.135	1.83E-02
Tap1	5.183	5.467	5.845	4.779	5.553	4.884	1.135	5.08E-03
Lmo7	4.703	4.911	5.636	3.460	4.068	4.429	1.133	1.79E-02
Aloxe3	5.117	5.552	5.837	4.143	4.557	4.350	1.132	5.21E-03
Myo18b	1.858	2.371	2.617	0.938	1.669	1.733	1.131	4.03E-02
Rgn	3.177	3.078	3.179	2.266	2.451	1.719	1.127	4.75E-02
Rcan3	6.001	6.245	6.617	4.449	5.455	5.432	1.123	2.41E-03
Calr3	3.615	3.753	4.005	2.526	3.006	2.735	1.119	3.61E-03
Fam65b	2.780	2.607	3.411	1.708	2.290	2.027	1.114	2.48E-02
Ggh	5.856	5.738	6.176	4.638	4.822	5.004	1.105	6.45E-04
LOC108348065	6.515	6.671	6.907	5.466	5.722	5.483	1.101	1.10E-03

Dnajb1	6.864	6.690	7.362	5.762	5.720	6.028	1.100	1.15E-03
Naa16	4.750	4.860	4.984	3.399	4.060	3.969	1.096	4.11E-05
Echdc1	5.243	5.095	5.643	4.147	4.409	4.362	1.079	2.04E-04
Ethe1	5.581	5.622	6.007	4.732	4.394	4.697	1.074	4.00E-03
Trip10	4.835	4.917	5.405	3.840	4.084	4.103	1.056	6.24E-04
Map3k6	3.038	3.128	3.600	2.249	2.398	2.275	1.053	8.09E-03
Lca5	3.577	3.804	4.277	2.347	3.232	3.088	1.050	8.16E-03
Znf354b	2.860	2.827	3.546	2.054	2.311	2.232	1.046	1.78E-02
Tspan6	6.792	6.944	7.267	5.763	6.120	6.021	1.039	3.37E-05
Relt	2.979	3.123	3.514	2.115	2.331	2.483	1.035	4.39E-03
Unc5d	3.198	3.155	3.668	1.715	2.885	2.595	1.027	2.38E-02
Mboat7l1	6.097	6.187	6.646	5.197	5.148	5.336	1.021	1.67E-03
Scn3a	4.097	4.208	4.289	2.420	3.702	3.424	1.018	3.74E-02
Ccdc14	2.757	2.928	3.345	1.862	2.279	2.297	1.015	1.15E-03
Hn1l	4.698	5.203	5.768	3.755	4.452	4.527	1.005	4.98E-02
P4ha1	4.620	4.830	5.430	3.424	4.131	4.389	1.005	1.65E-02
Golm1	3.846	4.168	4.927	3.178	3.704	3.368	1.005	3.65E-02
Ddx60	5.151	4.923	4.610	3.521	4.416	3.835	1.003	2.60E-02
Sec23b	4.983	5.258	5.584	3.753	4.491	4.574	0.999	7.98E-03
Calhm2	4.326	4.587	5.097	3.133	3.939	3.956	0.996	1.08E-02
Mtrf1l	5.510	5.451	6.070	4.606	4.698	4.809	0.994	2.41E-03
Slc30a2	3.841	3.650	4.028	2.904	2.987	2.847	0.987	2.12E-02
Fam210b	3.296	3.141	3.862	2.373	2.673	2.669	0.986	1.63E-02
Plekha7	3.313	3.772	4.259	2.142	3.173	3.196	0.983	3.47E-02
Sertad3	3.083	3.004	3.663	2.326	2.483	2.326	0.974	3.34E-02
Gprasp2	7.456	7.264	7.615	6.504	6.424	6.393	0.967	4.33E-03
Sgms2	3.070	3.081	3.541	1.725	2.597	2.735	0.952	3.92E-02
Zkscan2	3.098	3.353	3.432	1.953	2.910	2.469	0.940	1.22E-02
Tlcd1	1.728	2.243	2.202	1.200	1.592	1.356	0.939	3.54E-02

Mtus2	3.518	3.904	3.722	2.505	3.125	3.349	0.930	8.02E-03
Zfp853	2.654	2.487	3.293	2.101	1.957	2.099	0.923	4.54E-02
AABR07043445.1	5.924	6.199	6.530	4.427	5.607	5.616	0.921	2.89E-02
Tigar	5.371	5.368	5.944	4.442	4.573	4.931	0.916	2.32E-03
AABR07043564.1	5.255	5.149	5.843	4.471	4.597	4.502	0.910	2.58E-02
Mreg	3.877	3.806	3.923	2.895	3.246	2.787	0.900	7.05E-03
Vegfb	4.737	4.984	5.326	3.966	4.235	4.191	0.899	6.45E-04
Setdb2	3.543	3.845	4.029	2.532	3.326	3.033	0.899	8.44E-03
Nmt2	6.856	6.759	7.147	5.529	6.281	6.190	0.893	1.15E-03
Ptpn13	3.964	4.235	4.670	2.793	3.653	3.758	0.892	4.86E-02
Tmem255a	3.657	3.524	3.463	2.220	3.268	2.599	0.891	4.76E-02
Pomt1	4.788	5.250	5.550	4.023	4.591	4.240	0.889	1.15E-03
Abhd2	6.278	6.240	6.735	4.707	5.765	5.944	0.888	3.17E-02
AABR07043510.1	4.347	4.546	4.869	3.538	4.000	3.694	0.888	4.34E-02
Nfatc3	4.473	4.575	5.010	3.247	4.064	3.977	0.882	4.58E-03
Rhpn1	4.859	5.478	5.277	4.113	4.464	4.327	0.880	2.18E-02
Plod2	4.389	4.247	4.740	3.228	3.687	3.954	0.874	2.76E-02
LOC619574	5.272	5.254	5.835	4.475	4.626	4.612	0.872	5.08E-03
Rmt1	2.799	2.784	3.110	2.017	2.212	2.214	0.871	5.60E-03
Hsp90ab1	11.121	11.128	11.325	10.266	10.105	10.344	0.870	1.16E-02
Prim2	4.610	4.393	4.862	3.845	3.770	3.588	0.869	2.40E-02
Lmo2	5.747	5.659	6.300	4.996	5.139	4.985	0.863	2.72E-02
Zfp516	3.784	4.024	4.327	2.479	3.581	3.490	0.854	3.69E-02
Zfp365	4.311	4.018	4.509	3.151	3.618	3.608	0.852	8.09E-03
Mcam	6.418	6.355	6.652	5.476	5.648	5.698	0.839	2.72E-02
Camkk1	5.006	4.992	5.397	4.187	4.394	4.254	0.834	6.23E-03
Pacsin2	6.328	6.325	6.998	5.310	5.629	6.105	0.833	2.04E-02
Gtf2f2	5.313	5.355	5.693	4.569	4.610	4.712	0.827	5.98E-03
Atad2	4.240	4.452	4.796	2.972	4.046	4.017	0.826	4.76E-02

Ift27	5.366	5.273	5.591	4.681	4.395	4.471	0.825	3.06E-02
Edrf1	5.575	5.743	6.067	4.624	5.225	5.030	0.825	4.24E-04
Hspa4	8.012	7.938	8.289	6.957	7.316	7.489	0.818	1.12E-03
Timp3	4.841	4.672	5.195	3.850	4.310	4.025	0.818	9.24E-03
LOC361646	3.535	3.687	4.194	2.507	3.216	3.360	0.815	1.09E-02
Apba3	3.526	3.748	4.216	2.945	3.075	3.140	0.811	2.72E-02
Cadm1	5.739	5.529	6.277	4.410	5.335	5.410	0.809	3.56E-02
Anxa11	5.233	5.554	5.840	4.388	4.816	4.910	0.806	4.76E-02
Snx3	8.866	8.853	8.964	8.159	7.861	7.994	0.802	7.98E-03
Rab39a	5.145	5.071	5.630	4.166	4.625	4.744	0.797	7.45E-04
Opa3	4.887	4.847	5.211	4.006	4.112	4.360	0.795	2.07E-03
Gprasp1	7.709	7.558	7.750	6.694	7.012	6.877	0.792	4.57E-03
Jak3	3.266	3.710	3.850	2.632	3.154	2.740	0.787	2.45E-02
Ahsa2	6.064	6.088	6.296	5.141	5.469	5.448	0.786	8.96E-04
Zfp449	2.952	2.937	3.619	2.033	2.543	2.834	0.786	1.36E-02
Cacna2d2	5.080	5.053	5.646	4.182	4.496	4.663	0.780	1.24E-02
Lap3	5.754	5.977	6.306	5.094	5.320	5.269	0.779	4.57E-03
Efr3b	6.618	6.703	6.503	5.303	6.093	5.950	0.774	3.69E-02
Inpp5a	5.057	5.058	5.711	4.326	4.368	4.792	0.767	1.72E-02
Slc16a14	5.401	5.500	5.938	4.165	5.028	5.112	0.763	3.25E-02
Dnaja4	5.859	5.786	6.226	5.056	5.210	5.285	0.762	5.17E-03
Hspa4l	5.432	5.531	5.782	4.269	5.128	5.033	0.760	4.70E-03
Cct8	8.269	8.115	8.374	7.470	7.372	7.619	0.753	1.44E-02
Gusb	3.449	3.672	4.357	2.724	3.298	3.388	0.753	3.17E-02
Iqcb1	5.841	5.881	6.297	5.241	5.264	5.208	0.752	5.13E-03
Mphosph9	4.198	4.471	4.840	2.841	3.987	3.951	0.752	2.72E-02
Prdm15	3.022	3.198	3.631	2.187	2.791	2.855	0.749	1.00E-02
Bahd1	5.208	5.148	5.574	4.371	4.595	4.648	0.744	1.60E-03
Ogfod1	4.264	4.357	4.751	3.200	3.903	4.041	0.741	4.96E-02

Actr5	3.172	3.257	3.848	2.622	2.741	2.865	0.741	2.77E-02
Trim65	5.080	5.223	5.680	4.406	4.714	4.612	0.736	1.54E-02
Gnpda1	5.518	5.600	6.283	4.682	5.198	5.302	0.733	3.54E-02
Ngdn	7.080	7.391	7.399	6.522	6.640	6.427	0.729	4.08E-03
Prkab2	4.285	4.335	5.003	3.337	3.897	4.129	0.728	3.24E-02
Pdxk	6.543	6.730	6.591	5.156	6.128	6.187	0.727	2.72E-02
Dnaaf2	3.872	3.941	4.295	3.035	3.357	3.561	0.719	1.16E-02
Pusl1	4.873	4.910	5.022	3.798	4.442	4.411	0.710	7.73E-03
Tp53bp1	6.625	6.707	6.872	5.534	6.296	6.128	0.706	8.55E-03
Nup210	3.958	4.086	4.566	3.036	3.561	3.907	0.699	1.26E-02
Hyal3	4.105	4.485	4.438	3.556	3.779	3.596	0.684	4.69E-03
Khnyn	4.456	4.485	4.860	3.226	4.307	4.135	0.683	4.08E-02
Mrps7	6.341	6.346	6.677	5.806	5.752	5.641	0.677	4.26E-02
Agpat5	5.168	5.217	5.555	4.200	4.715	4.983	0.677	6.25E-03
Cept1	5.801	5.606	6.080	4.941	5.238	5.309	0.675	2.08E-03
Pigh	4.238	3.809	4.516	3.484	3.555	3.656	0.672	2.72E-02
LOC100910854	4.025	4.164	4.530	3.447	3.521	3.824	0.662	2.89E-02
Erp29	6.992	7.196	7.527	6.516	6.597	6.660	0.660	1.18E-02
Zfyve26	3.618	3.680	3.996	2.522	3.431	3.366	0.654	2.21E-02
Uba7	2.961	3.065	3.269	2.503	2.466	2.466	0.654	1.89E-02
Parm1	4.674	4.826	5.014	3.929	4.347	4.208	0.653	3.54E-02
Cdan1	3.668	3.767	4.044	2.624	3.441	3.450	0.653	1.09E-02
Tiam2	3.853	4.119	4.519	3.118	3.763	3.663	0.652	3.49E-02
Unc13a	5.973	6.108	6.449	4.926	5.747	5.761	0.649	1.64E-02
Wipf2	4.375	4.451	4.675	3.272	4.121	3.977	0.641	1.57E-02
Chrnb1	2.214	2.446	2.524	1.604	1.985	2.006	0.638	4.54E-02
Arl6ip4	5.176	5.505	5.841	4.855	4.681	4.885	0.637	4.69E-02
Fam118a	5.288	5.142	5.611	4.542	4.727	4.794	0.635	8.31E-03
Gpr68	5.776	5.905	6.166	5.149	5.377	5.287	0.635	3.47E-02

Ap4e1	4.546	4.623	4.881	3.350	4.304	4.437	0.631	3.27E-02
Borcs6	5.080	5.267	5.605	4.651	4.614	4.706	0.630	3.16E-02
Zfp593	3.901	4.037	4.393	3.425	3.403	3.638	0.630	3.44E-02
Dhx29	4.258	4.249	4.779	3.464	3.884	4.080	0.629	1.58E-02
RGD1563091	6.045	5.769	5.506	4.923	5.383	5.220	0.629	3.75E-02
Lmnb2	4.203	4.234	4.622	3.470	3.682	4.066	0.625	2.58E-02
Slc35c1	5.360	5.371	5.909	4.744	4.817	5.099	0.622	2.77E-02
Ero1a	5.277	5.236	5.809	4.283	5.039	5.157	0.620	3.66E-02
Elovl5	6.454	6.583	6.998	5.663	6.149	6.288	0.616	1.76E-02
RGD1304624	4.944	4.997	5.440	4.378	4.556	4.610	0.615	4.92E-02
Asl	5.160	5.641	5.736	4.497	5.064	5.080	0.613	1.69E-02
Dnaja1	8.752	8.625	8.877	7.986	8.151	8.331	0.612	4.20E-03
Bcap31	7.275	7.034	7.430	6.559	6.611	6.645	0.608	1.06E-02
Akip1	4.083	4.101	4.572	3.563	3.672	3.747	0.600	4.76E-02
Atg9a	6.043	6.186	6.324	5.487	5.590	5.479	0.597	2.38E-02
Rock2	6.430	6.479	6.661	4.961	6.173	6.245	0.597	3.49E-02
Xrcc2	2.850	2.661	3.283	2.135	2.509	2.584	0.592	4.26E-02
Nsun5	5.163	5.368	5.562	4.714	4.690	4.800	0.589	1.63E-02
Cd99	4.939	5.024	5.382	5.484	5.724	5.694	-0.585	4.12E-02
Mfsd10	3.533	4.189	4.086	4.261	4.515	4.538	-0.586	2.12E-02
Gss	2.904	3.294	3.096	3.507	3.681	3.544	-0.592	4.89E-02
Nfkbie	3.920	4.396	4.130	4.448	4.782	4.724	-0.603	2.95E-02
Tmsb4x	9.762	9.700	9.990	10.429	10.227	10.502	-0.605	3.47E-02
Zfyve21	3.105	3.269	3.399	3.504	3.928	3.997	-0.643	1.43E-02
B3gat1	3.779	3.588	3.639	4.129	4.112	4.407	-0.646	1.56E-02
Chrd	2.443	2.424	2.237	2.793	2.849	2.957	-0.650	3.00E-02
Hes6	5.966	6.212	5.936	6.577	6.673	6.552	-0.657	1.69E-02
Gadd45a	5.793	6.010	5.691	6.197	6.756	6.484	-0.669	2.71E-02
Plekhj1	4.735	5.028	4.895	5.485	5.561	5.347	-0.672	1.93E-02

Adamts10	2.717	3.126	2.825	3.334	3.625	3.314	-0.684	4.92E-02
Slc35b2	4.076	4.419	4.141	4.648	4.909	4.914	-0.692	1.51E-03
Tmem200c	2.354	2.064	2.359	2.443	2.970	2.965	-0.700	2.26E-02
Rfxap	4.612	4.679	4.863	5.384	5.224	5.493	-0.713	1.03E-02
Gls2	3.158	3.379	3.354	3.817	3.948	3.993	-0.723	1.36E-02
Bcas1	4.593	4.497	4.568	4.981	5.172	5.507	-0.736	4.98E-03
Tle2	1.304	1.604	1.696	1.715	1.980	2.355	-0.754	4.41E-02
Cdh20	2.699	2.680	2.871	3.069	3.277	3.750	-0.757	8.01E-03
Ifngr1	3.917	4.069	4.192	4.392	4.888	4.934	-0.775	8.09E-03
Nrbp2	4.367	5.016	4.749	5.211	5.701	5.427	-0.806	2.16E-02
Tshr	1.289	1.153	1.136	1.395	1.815	1.855	-0.821	1.60E-02
Trib3	3.193	3.293	3.489	3.690	4.332	4.158	-0.857	3.73E-02
Sfrp2	4.391	4.320	4.691	4.824	5.245	5.640	-0.861	7.64E-03
Rgs10	5.204	5.082	5.094	6.129	5.816	5.736	-0.907	3.54E-02
Nxph4	4.070	4.087	4.096	5.119	4.748	4.686	-0.938	2.72E-02
Ctgf	1.215	1.458	1.576	1.688	2.097	2.578	-1.046	3.47E-02
Cpne7	1.647	2.091	1.985	2.400	2.739	2.972	-1.047	2.45E-02
LOC108351703	6.006	6.079	6.591	7.731	6.764	7.387	-1.220	4.22E-02
Scn4b	0.315	0.291	0.216	0.442	0.718	0.619	-1.283	3.54E-02
AABR07024498.1	3.765	3.692	4.369	5.962	5.775	5.873	-2.037	4.12E-02
Mmp3	0.977	0.896	2.271	3.309	4.345	3.140	-2.709	2.36E-02

Table S3. Gene ontology analysis for the HSF1 target genes in apoptotic CGNs. Gene ontology analysis was performed using DAVID software for the HSF1-regulated genes identified from LK-treated CGNs. "pop total" is the total number of genes included in Biological Process (GO_BP) or Molecular Function (GO_MF) of the Gene ontology database; "pop hit" is the number of genes belong to a pathway and also in the GO_BP or GO_MF; "list hit" is the number of genes belong to our DEGs and also in the GO_BP or GO_MF.

	**Category**	**Term**	**Genes**	**Count**	**List total**	**Pop hits**	**Pop total**	***P* value**
**Top 9 biological processes or molecular functions HSF1 target genes are involved in**	GOTERM_BP_DIRECT	Protein transport	CALCR, KDELR3, ATG9A, LCA5, NUP210, LMAN2L, TAP1, LIN7B, APBA3, SNX3	10	319	257	17535	0.0454
	GOTERM_MF_DIRECT	ATP binding	HSP90AB1, ACVRL1, XRCC2, FIGNL1, UBA7, TRIB3, CAMKK1, GSS, MAP3K6, HSPH1, DDX60, WNK4, TAP1, DNAJA1, HSPA4, DNAJA4, DDX43, NLRP6, PDXK, ROCK2, ALPK2, ATAD2, MYH7, MYO1H, MYO18B, EPHA6, DHX29, GCK, ULK3, GTF2F2, CCT8, HSPA4L, JAK3, BMPR1B, MELK, NRBP2	36	298	1403	16650	0.0269
	GOTERM_MF_DIRECT	Calcium ion binding	TCHH, MATN4, EGFEM1, CALR4, SYT5, MMP3, ANXA4, ANXA3, CDH20, DSG2, COMP, ANXA11, ACAN, RGN, SYTL5, CALR3, CIB2, CASQ2, MELK, THBS4, CDH23	21	298	696	16650	0.0249
	GOTERM_BP_DIRECT	Negative regulation of gene expression	VEGFB, CRHR2, ACVRL1, SFRP2, CTGF, CRYAB, ERP29, ESR1, HSPA4, LGALS9	10	319	212	17535	0.0156
	GOTERM_MF_DIRECT	Iron ion binding	OGFOD1, PLOD1, ALOXE3, P4HA1, CYP2D4, PLOD2, ETHE1, CYP2S1, SCD, AOX1	10	298	202	16650	0.0106
	GOTERM_BP_DIRECT	Intracellular signal transduction	ADCY7, MPDZ, MCF2, ARHGEF28, DMRT1, VAV1, TIAM2, CTGF, WNK4, HSPB1, JAK3, RASA4, UNC13A, MELK, NRBP2	15	319	381	17535	0.0100
	GOTERM_BP_DIRECT	Cell adhesion	VWF, LYVE1, CADM1, DSG2, MPDZ, CTGF, COMP, ACAN, MADCAM1, LAMC2, MCAM, THBS4	12	319	261	17535	0.0083
	KEGG_PATHWAY	Protein processing in endoplasmic reticulum	HSP90AB1, HSPH1, SEC31B, ERO1A, CRYAB, ERP29, HSPA4L, DNAJA1, DNAJB1, SEC23B, BCAP31	11	135	168	7780	0.0006
	GOTERM_BP_DIRECT	Protein folding	HSP90AB1, ERO1A, CALR4, CRYAB, CCT8, ERP29, HSPA4L, DNAJA1, CALR3, DNAJB1	10	319	112	17535	0.0002

**Apoptotic processes regulated by HSF1**	GOTERM_BP_DIRECT	Positive regulation of dendritic cell apoptotic process	JAK3, LGALS9	2	319	4	17535	0.0706
	GOTERM_BP_DIRECT	Positive regulation of cysteine-type endopeptidase activity involved in apoptotic process	CTGF, NODAL, PMAIP1, BCAP31	4	319	54	17535	0.0746
	GOTERM_BP_DIRECT	Negative regulation of apoptotic process	HSP90AB1, PDXK, FIGNL1, BAG3, CRYAB, BTC, ASIC2, NCKAP1L, NAA16, VEGFB, CARD14, COMP, DNAJA1, RGN, HSPB1, HSPA4	16	319	517	17535	0.0893
	GOTERM_BP_DIRECT	Regulation of apoptotic process	GLS2, IKZF3, RELT, SFRP2, NODAL, ESR1, ALX4	7	319	170	17535	0.0897

Table S4. Function annotation for HSF1 target genes in apoptotic CGNs in neurological diseases. Function annotation for HSF1-regulated genes in LK-treated CGNs was conducted using IPA. The function of these genes in neurological diseases, the *P* value, and the genes involved in each subcategory are shown.

**Diseases or function annotation**	***P* value**	**Molecules**
Autosomal recessive deafness type 12	7.61E-04	Cdh23, Gjb2
Familial slow-channel congenital myasthenic syndrome	7.61E-04	Chrnb1, Chrnd
Schizoaffective disorder	9.85E-04	Chrnb1, Chrnd, Esr1, Esr2, Gss, Inpp5a, Scn3a, Scn4b
Hydrocephalus	1.93E-03	Apob, Chrnb1, Chrnd, Dnaaf2, Dnai1, Mns1, Mpdz
Congenital myasthenic syndrome associated with acetylcholine receptor deficiency	3.69E-03	Chrnb1, Chrnd
Usher syndrome type 1B	6.74E-03	Cdh23, Cib2
Cervical spondylotic myelopathy	7.24E-03	Cacna2d2, Scn3a, Scn4b
Apoptosis of neural precursor cells	1.06E-02	Rock2, Xrcc2
Loss of hair cells	1.28E-02	Gjb2, Thrb
Familial prelingual deafness	1.48E-02	Cdh23, Cib2, Dya4, Gjb2
Migraines	1.49E-02	Cacna2d2, Esr1, Esr2, Pde4c, Scn3a, Scn4b, Vwf
Muscle-eye-brain disease	1.52E-02	B3galnt2, Pomt1
Congenital malformation of brain	1.55E-02	Acvrl1, Alx4, Apob, B3galnt2, Bcap31, Cacna2d2, Chrd, Cntnap2, Gadd45a, Lmnb2, Mpdz, Pomt1, Prokr2, Xrcc2

Table S5. The statistics of DEGs found with different stringencies of cutoff. 1.5 and 1 fold-changes were applied together with FDR control of 0.05 and 0.1. The numbers show up-regulated genes found in the condition listed in each row. For example, 969 genes are up-regulated in HK condition comparing HSF1 with HSF1-AB, while 357 genes are up-regulated in HK condition comparing HSF1-AB with HSF1, which means these 357 genes are down-regulated by HSF1 in HK when comparing with HSF1-AB. The up- and down-regulated genes by HSF1-AB comparing with GFP control are shown in bold fonts with gray background and highlighted in red and blue colors respectively.

		Fold change > 1.5							Fold change > 1					
		**HSF1 HK**	**HSF1-AB HK**	**GFP HK**	**HSF1 LK**	**HSF1-AB LK**	**GFP LK**		**HSF1 HK**	**HSF1-AB HK**	**GFP HK**	**HSF1 LK**	**HSF1-AB LK**	**GFP LK**
**HSF1 HK**	**FDR < 0.1**	0	1144	1254	1757	2193	2126		0	1607	1696	3709	4021	3901
**HSF1-AB HK**		514	0	**22**	2007	1411	1466		854	0	**22**	3895	3200	3245
**GFP HK**		233	**1**	0	2106	1749	1504		488	**1**	0	3953	3514	3293
**HSF1 LK**		3280	3742	4034	0	401	517		4205	4731	5062	0	600	732
**HSF1-AB LK**		3340	3166	3693	150	0	**11**		4286	4036	4681	278	0	**11**
**GFP LK**		3118	3027	3344	58	**0**	0		4100	3915	4262	168	**0**	0
**HSF1 HK**	**FDR < 0.05**	0	969	1075	1636	2052	1995		0	1255	1347	3025	3345	3315
**HSF1-AB HK**		357	0	**13**	1873	1301	1327		531	0	**13**	3295	2542	2586
**GFP HK**		136	**0**	0	1977	1616	1372		256	**0**	0	3327	2865	2618
**HSF1 LK**		2899	3471	3762	0	292	393		3513	4177	4489	0	388	506
**HSF1-AB LK**		3002	2852	3351	91	0	**6**		3642	3429	4047	139	0	**6**
**GFP LK**		2745	2708	2977	32	**0**	0		3416	3316	3589	68	**0**	0

Table S6. The DEGs responding to HSF1-AB in HK and LK conditions. Particularly, those DEGs with FDR between 0.05 and 0.1 were shown with blue background. Uba7 is the only DEG found in both HK and LK conditions (shown with yellow background).

HK									
Genes	HSF1-AB HK-1	HSF1-AB HK-2	HSF1-AB HK-3	GFP HK-1	GFP HK-2	GFP HK-3	logFC	*P* Value	FDR
Angptl1	0.66	0.98	1.04	0.00	0.00	0.00	6.71	6.87E-07	0.0028
Llgl2	0.78	1.09	1.47	0.17	0.30	0.30	2.44	5.24E-07	0.0028
Oas1a	4.36	4.81	5.04	3.43	3.67	3.92	1.05	7.08E-07	0.0028
Vdr	0.40	0.66	0.66	0.07	0.08	0.09	2.98	9.17E-07	0.0029
Klc3	1.62	2.16	2.48	0.63	0.81	0.60	2.35	1.68E-06	0.0044
Uba7	3.47	3.63	3.67	2.70	2.57	2.60	1.00	4.09E-06	0.0093
Trpv4	0.39	0.59	0.67	0.06	0.07	0.14	2.72	1.08E-05	0.0172
Kcp	0.43	0.73	0.69	0.07	0.18	0.12	2.46	1.04E-05	0.0172
Cyp2d4	1.08	1.42	1.11	0.26	0.25	0.44	2.27	9.28E-06	0.0172
Slc16a3	1.63	2.30	2.39	0.53	0.89	0.93	2.12	1.33E-05	0.0177
Hck	2.60	2.79	3.53	1.36	1.22	1.83	1.86	1.25E-05	0.0177
Pik3ap1	0.71	1.33	1.14	0.12	0.15	0.26	3.05	1.98E-05	0.0242
Slc44a4	0.71	1.02	1.02	0.17	0.33	0.04	2.60	4.28E-05	0.0485
Alox15	0.92	1.94	1.70	0.23	0.27	0.50	2.75	5.64E-05	0.0596
Fstl3	0.79	1.73	1.68	0.06	0.46	0.40	2.72	8.55E-05	0.0781
Nudt17	2.25	2.18	3.25	1.09	0.97	1.61	1.73	8.03E-05	0.0781
Hpcal4	3.66	3.98	4.52	2.78	3.13	3.26	1.01	8.86E-05	0.0781
Phldb3	0.66	1.20	1.57	0.10	0.44	0.11	2.80	1.03E-04	0.0815
Fads3	4.04	4.32	4.62	3.30	3.55	3.89	0.69	1.02E-04	0.0815
Tmem106a	1.72	2.96	2.86	0.46	1.09	1.10	2.42	1.27E-04	0.0960
Naaa	1.80	2.52	2.42	1.26	1.48	1.55	1.06	1.50E-04	0.0995
Ccdc136	3.99	4.23	4.47	3.53	3.41	3.70	0.65	1.42E-04	0.0995
Fam84a	4.60	4.59	4.70	5.11	5.08	5.69	-0.80	1.47E-04	0.0995

LK									
Genes	HSF1-AB LK-1	HSF1-AB LK-2	HSF1-AB LK-3	GFP LK-1	GFP LK-2	GFP LK-3	logFC	*P* Value	FDR
Nfam1	4.18	5.02	5.98	0.15	0.51	0.69	6.55	1.87E-07	0.0015
Krt13	2.84	3.47	4.86	0.60	1.25	1.09	3.77	3.31E-06	0.0131
Dhcr7	6.40	6.23	6.65	4.94	4.52	4.21	1.76	2.52E-06	0.0131
Apob	0.49	1.23	2.49	0.01	0.07	0.01	6.58	6.44E-06	0.0205
B3gnt7	3.35	3.60	4.32	2.31	2.67	2.35	1.44	9.12E-06	0.0241
Ntf4	1.06	1.69	2.63	0.06	0.17	0.32	4.22	2.11E-05	0.0479
Tmprss9	4.27	5.07	5.91	1.95	2.61	2.80	2.92	2.60E-05	0.0515
Tmem51	4.01	4.25	5.03	3.10	3.34	3.47	1.15	4.31E-05	0.0713
Cnp	6.53	6.66	7.45	5.69	5.87	6.14	0.95	4.49E-05	0.0713
Uba7	3.13	3.23	3.36	2.50	2.47	2.47	0.80	5.01E-05	0.0723
